# NIBAN2/FLII/RREB1 Axis Drives Glioma Stem Cell Malignancy via TLR3 Pathway Activation

**DOI:** 10.1002/advs.202519382

**Published:** 2026-02-25

**Authors:** Liang liang Shi, Xinwei Qiao, Yue Hu, Minjie Wang, Shaojie Yu, Zihan Gong, Yuxin Rao, Hui Zheng, Siting Chen, Lin Li, Rong Fu, Tao Liang, Jin Yao, Xiaobing Jiang, Junjun Li

**Affiliations:** ^1^ Cancer Center Union Hospital, Tongji Medical College Huazhong University of Science and Technology Wuhan Hubei China; ^2^ Department of Thoracic Surgery Union Hospital, Tongji Medical College Huazhong University of Science and Technology Wuhan Hubei China; ^3^ Department of Neurosurgery Union Hospital, Tongji Medical College Huazhong University of Science and Technology Wuhan Hubei China; ^4^ Department of Radiology Union Hospital, Tongji Medical College Huazhong University of Science and Technology Wuhan Hubei China; ^5^ Department of Emergency Union Hospital Tongji Medical College Huazhong University of Science and Technology Wuhan Hubei China; ^6^ Department of Clinical Laboratory Union Hospital Tongji Medical College Huazhong University of Science and Technology Wuhan Hubei China; ^7^ Hubei Key Laboratory of Biological Targeted Therapy Union Hospital Tongji Medical College Huazhong University of Science and Technology Wuhan Hubei China

**Keywords:** glioma stem cells, metabolic reprogramming, NIBAN2/FLII/RREB1, TLR pathway

## Abstract

Glioma stem‐like cells (GSCs), characterized by self‐renewal capacity, therapeutic resistance, and high tumorigenicity, are considered the fundamental drivers of glioma aggressiveness and recurrence. However, the core regulatory mechanisms underlying GSCs stemness maintenance remain unclear. In this study, we identified that the NIBAN2 is highly expressed in glioma tissues and GSCs, and its expression is closely associated with poor patient prognosis. Functional experiments demonstrated that NIBAN2 directly binds to Flightless I (FLII) and enhances its interaction with the transcription factor Ras‐responsive element‐binding protein 1 (RREB1), thereby promoting nuclear translocation. Together, the NIBAN2‐FLII‐RREB1 complex activates the Toll‐like receptor (TLR3) signaling pathway, sustaining the stem‐like phenotype and tumorigenic potential of GSCs. Further investigation revealed that RREB1 transcriptionally upregulates NIBAN2 and CD44, promoting the expression of the key glycolytic enzyme LDHA (Lactate Dehydrogenase A). This establishes a feed‐forward signaling‐transcription‐metabolism axis driven by NIBAN2/FLII/RREB1, facilitating metabolic reprogramming. Multi‐omics and metabolomic analyses confirmed that this loop enhances glycolytic flux to maintain GSCs’ metabolic homeostasis. Drug screening identified the HIV protease inhibitor nelfinavir as a specific disruptor of the NIBAN2‐FLII complex. When combined with the LDHA inhibitor FX11, it induced synergistic anti‐tumor effects in organoid and patient‐derived xenograft models, thereby significantly inhibiting tumor progression and prolonging survival. Clinical samples further confirmed the co‐upregulation of NIBAN2, FLII, and RREB1 in GBM (Glioblastoma) tissues, which correlates with SOX2 and Ki‐67 expression and poor prognosis. Collectively, this study, for the first time, reveals that NIBAN2 maintains GSCs stemness by activating FLII‐RREB1 axis and TLR3 signaling, thereby establishing a feed‐forward signaling‐transcription‐metabolism axis. This finding provides a novel strategy and potential targets for precise therapeutic intervention against glioma stemness.

## Graphical abstract

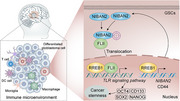

## 1 Introduction

Glioma is one of the most prevalent malignant tumors of the central nervous system, characterized by high aggressiveness and recurrence rates. Among its cellular components, glioma stem‐like cells (GSCs), which possess self‐renewal capability, therapeutic resistance, and tumorigenic potential, are considered key drivers of tumor initiation and progression [1, 2, 3, 4]. However, the development of effective and precise therapeutic strategies is restricted by the incomplete definition of the upstream regulatory mechanisms that maintain GSCs stemness. Recently, multiple studies have reported a complex and intimate interplay between tumor stemness, metabolic reprogramming, immune pathway activation, and transcriptional regulatory networks. However, the intrinsic coordination among these processes within the context of glioma remains poorly understood.

In this study, we systematically identified NIBAN2 as a key regulator of glioma stemness through integrative multi‐omics analyses, clinical specimen validation, and both in vitro and in vivo functional assays. We found that NIBAN2 was significantly upregulated in high‐grade glioma (HGG) tissues and GSCs, and its expression was strongly associated with poor patient prognosis. Functional experiments demonstrated that NIBAN2 promotes the nuclear translocation of its interacting partner Flightless I (FLII), thereby facilitating its complex formation with the transcription factor Ras‐responsive element‐binding protein 1 (RREB1). When combined, this NIBAN2‐FLII‐RREB1 complex activates the Toll‐like receptor (TLR3) signaling pathway, thereby sustaining GSCs stemness and promoting malignant tumor features. Further mechanistic studies revealed that RREB1 can transcriptionally upregulate NIBAN2 and CD44, establishing a stable NIBAN2‐FLII‐RREB1 feed‐forward signaling‐transcription‐metabolism axis. Additionally, this loop transcriptionally activates LDHA, driving aerobic glycolytic flux to maintain GSCs’ metabolic homeostasis. Based on these findings, we performed virtual screening and pharmacological validation, identifying Nelfinavir as an effective inhibitor that disrupts the NIBAN2‐FLII complex. When combined with the LDHA inhibitor FX11, this treatment exhibited a synergistic anti‐tumor effects in organoid and patient‐derived xenograft (PDX) models, significantly suppressing tumor progression and prolonging survival. Clinical tissue microarray analysis further confirmed the co‐expression of NIBAN2, FLII, and RREB1 in HGG. A strong colocalization was observed with stemness and proliferation markers, including SOX2 and Ki‐67. This highlights the critical role of this feed‐forward signaling‐transcription‐metabolism axis in maintaining tumor stemness and driving glioma progression.

In summary, this study is the first to identify that NIBAN2 maintains the stemness of GSCs by activating the FLII‐RREB1 axis and the TLR3 signaling pathway, while promoting malignant progression through metabolic reprogramming. NIBAN2 may maintain stemness in GSCs by modulating TLR3 signaling and enhancing glycolytic activity (via LDHA regulation). This mechanism has not been well established in other tumor types, underscoring the novelty of our findings. We propose a NIBAN2‐centered regulatory model that integrates the coordination of metabolic, transcriptional, and immune signaling. This model offers a novel strategy and potential therapeutic targets for the precise eradication of glioma stemness.

## 2 Results

### 2.1 NIBAN2 is Highly Expressed in Glioma Tissues and is Associated with Poor Patient Prognosis

**FIGURE 1:**
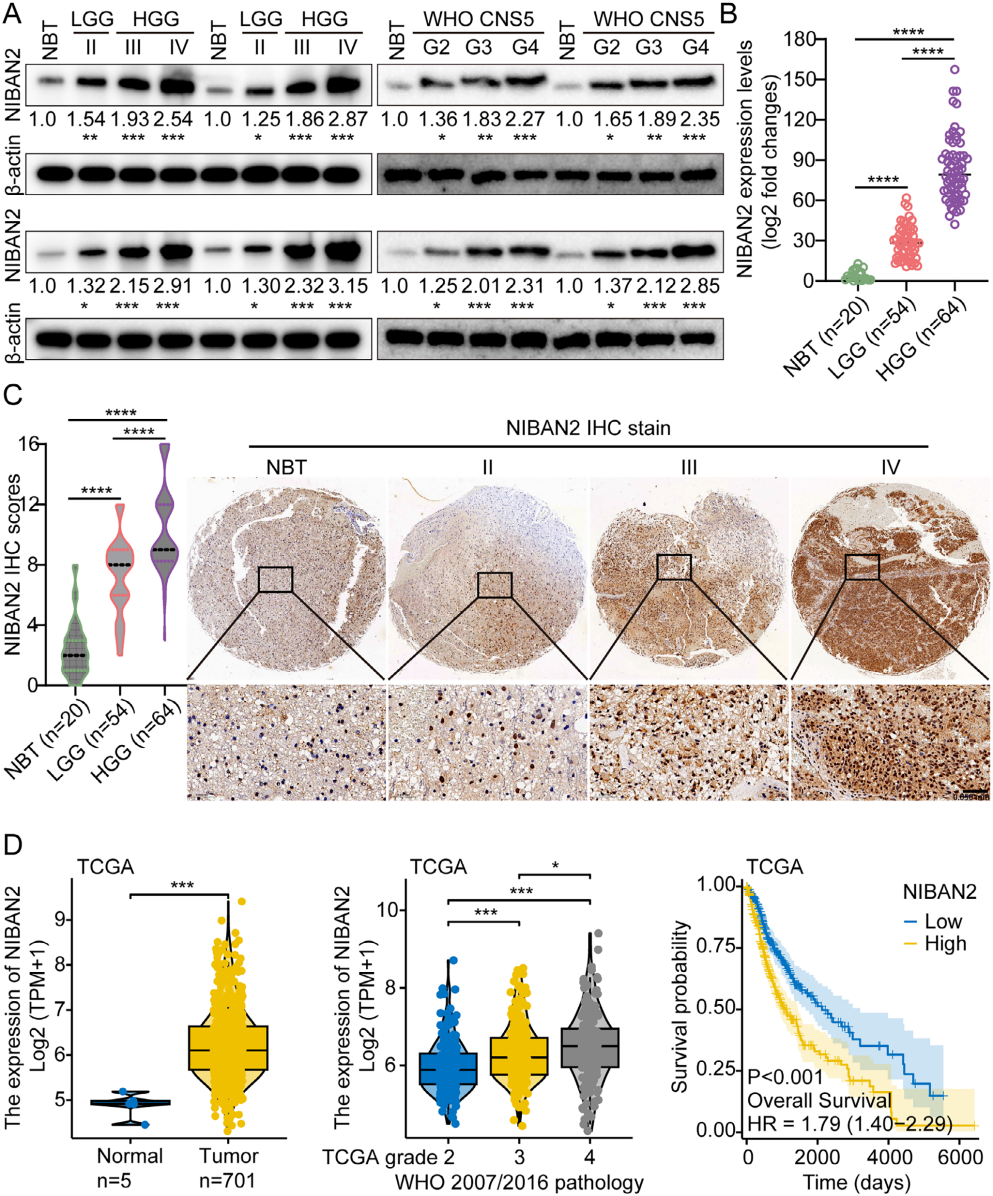
NIBAN2 is upregulated in glioma and correlates with tumor grade and poor prognosis. (A,B) WB (A) and qPCR (B) analyses of NIBAN2 expression in clinical samples of NBT, LGG, and HGG. (C) Representative IHC images exhibiting NIBAN2 expression in NBT, LGG, and HGG tissues. Scale bars, 0.05 mm. (D) Kaplan‐Meier survival analysis of glioma patients from the TCGA dataset stratified by NIBAN2 expression. Data were mean ± SD. Statistical significance was calculated by 1‐way ANOVA for A, B, C, D (middle) and F; 2‐tailed unpaired Student's *t* tests for D (left); Survival analysis of D (right) was performed by the log‐rank test. ^*^ *p* < 0.05, ^**^ *p* < 0.01, ^***^ *p* < 0.001, ^****^ *p* < 0.0001.

To systematically identify the core regulators involved in maintaining the GSCs' stemness, we initially collected three groups of clinical samples: normal brain tissues (NBT), low‐grade glioma (LGG) tissues, and HGG tissues. Protein microarray analysis was performed on these samples (Figure S1A–C; Tables S1 and S2). By intersecting the differentially expressed proteins across groups using a Venn diagram (Figure S1D), we identified NIBAN2 as a potential key candidate. To further validate the expression pattern of NIBAN2, we conducted Western blot (WB), quantitative PCR (qPCR), and immunohistochemistry (IHC) on clinical samples. The results indicated that NIBAN2 expression was significantly upregulated in glioma tissues compared to NBT, and its expression level progressively increased with tumor grade (Figures 1A–C). In addition, we established eight primary GBM cell lines and induced them into GSCs. WB analysis revealed that NIBAN2 expression was relatively low in cell lines 1–4; however, it was significantly upregulated in cell lines 5–8. Compared to the corresponding parental GBM cells, NIBAN2 expression was generally increased in GSCs (Figure S1E), suggesting a strong association with the stem‐like phenotype. Furthermore, data analysis from The Cancer Genome Atlas (TCGA) database corroborated our findings: NIBAN2 is significantly overexpressed in glioma tissues, and its high expression is strongly correlated with poor patient prognosis (Figure 1D; Figure S2A).

### 2.2 NIBAN2 Promotes Malignant Phenotypes of GSCs

**FIGURE 2:**
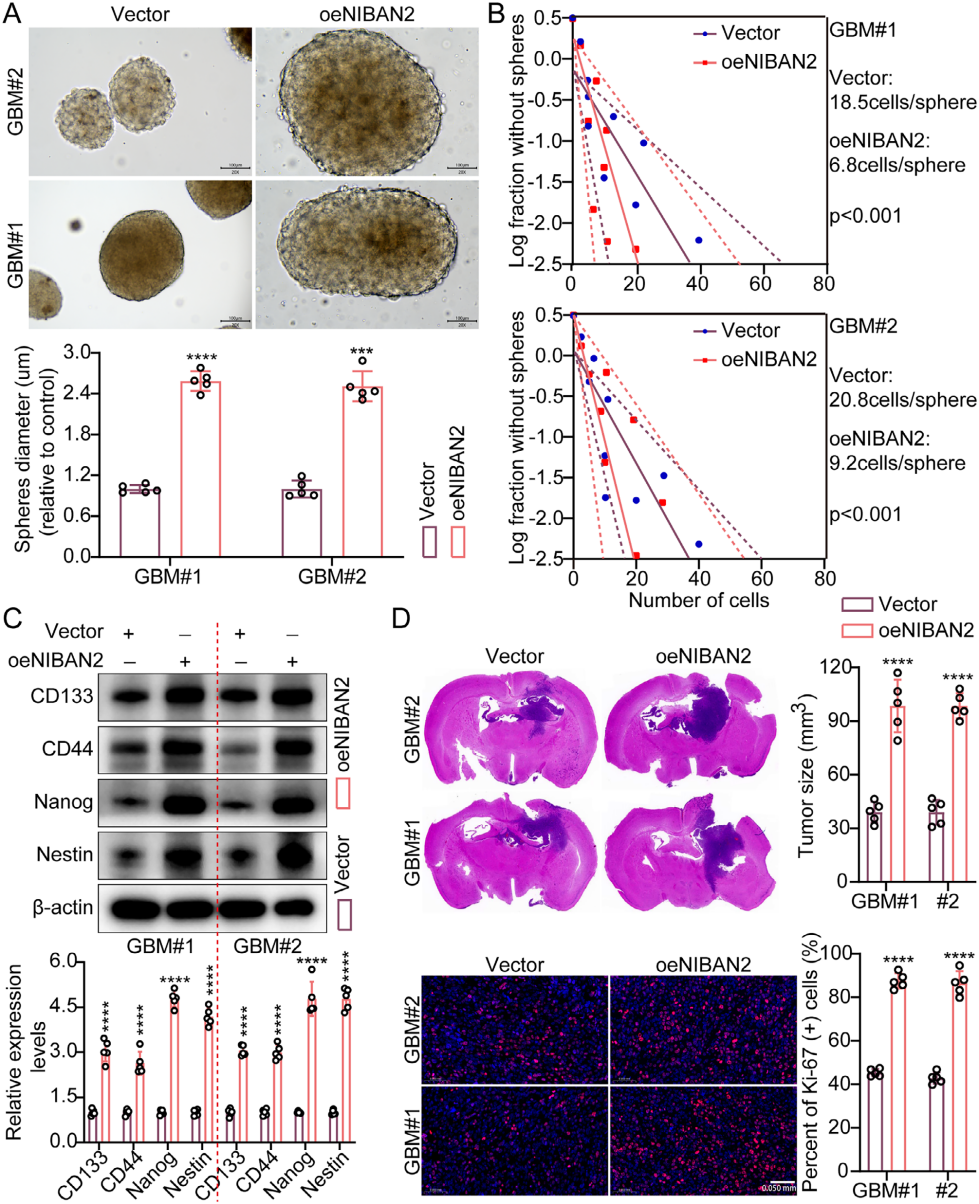
NIBAN2 promotes stemness and malignant phenotypes in GSCs. (A) Representative images and quantification of tumor sphere formation in control and NIBAN2‐overexpressing GSCs cultured under serum‐free, low‐adhesion conditions. Sphere‐forming efficiency and average diameter were significantly increased in the NIBAN2 group. Scale bars, 100 µm. (B) LDA determining the self‐renewal capacity of GSCs with or without NIBAN2 overexpression. The frequency of stem‐like cells was calculated using the ELDA algorithm. (C) WB analysis of stemness‐associated markers (CD133, CD44, NANOG, and Nestin) in control and NIBAN2‐overexpressing GSCs. β‐actin served as the loading control. (D) Representative tumor sections and Ki67 immunofluorescence staining of immunodeficient mice bearing intracranial xenografts of control or NIBAN2‐overexpressing GSCs; the corresponding quantitative bar graphs are shown on the right (*n* = 5). Scale bars, 0.05 mm. Data were mean ± SD. Statistical significance was calculated by 2‐tailed unpaired Student's *t* tests for A–D. ^***^ *p* < 0.001, ^****^ *p* < 0.0001.

To further investigate the critical role of NIBAN2 in maintaining the stemness of GSCs, we performed a series of systematic in vitro and in vivo functional assays. First, in tumor sphere formation assays, NIBAN2 overexpression significantly increased the sphere‐forming ability of GSCs under serum‐free and low‐attachment conditions. Compared with the control group, the NIBAN2‐overexpressing group formed a higher number of spheres, which were larger and more compact in structure. Quantitative analysis revealed that both sphere‐forming efficiency and average sphere diameter increased approximately 2.5‐fold compared to the controls (Figure 2A), indicating that NIBAN2 promotes the self‐renewal capacity and stemness maintenance of GSCs. Second, we conducted limiting dilution assays (LDA) to further evaluate the effect of NIBAN2 on GSCs sphere formation at different seeding densities. NIBAN2 overexpression significantly increased the frequency of sphere‐positive wells across all densities and maintained a high sphere‐forming capacity even under low‐cell conditions. Extreme limiting dilution analysis (ELDA) model analysis revealed that NIBAN2 overexpression significantly increased the frequency of stem‐like cells, from 1 in 19.65 in the control group to 1 in 8.0 (*p* < 0.001) (Figure 2B), further confirming its role in increasing stem‐like properties. Third, we performed soft agar colony formation assays to determine anchorage‐independent growth. NIBAN2 overexpression significantly increased both the number and average diameter of colonies by approximately 2.0‐fold (*p* < 0.01) (Figure S2B). This exhibited its potential to promote the growth and proliferation of GSCs under non‐adherent conditions, thereby supporting its stemness‐promoting function. Next, we determined the expression of key stemness markers. WB analysis revealed that NIBAN2 overexpression significantly upregulated classical stemness‐related factors, including CD133, CD44, NANOG, and Nestin (Figure 2C). This suggested that NIBAN2 can maintain the stemness of GSCs through transcriptional regulation of stemness‐associated programs.

For vivo validation, we established an orthotopic xenograft mouse model. Upon intracranial injection of NIBAN2‐overexpressing GSCs into immunodeficient mice, the experimental group exhibited significantly shorter survival (Figure S2C), higher tumor incidence, and larger tumor volumes. Additionally, IHC and fluorescence analyses confirmed upregulated expression of SOX2 and Ki‐67 in tumor tissues (Figure 2D; Figure S2D). This suggests that NIBAN2 increases the tumorigenic potential and stem‐like characteristics of GSCs in vivo. Consistently, knockdown of NIBAN2 in GSCs resulted in the opposite phenotypes in both in vitro and in vivo experiments, including reduced sphere and colony formation, downregulation of stemness markers, and reduced tumorigenicity (Figures S2E and S3A–D). Of course, we also found that NIBAN2 expression is positively correlated with the GSC stemness markers CD133 and Nestin. After induction into GSCs, cell lines with higher NIBAN2 expression exhibited a more pronounced stem‐like phenotype, including higher levels of CD133 and Nestin, supporting an important role of NIBAN2 in maintaining stemness traits in GBM cells (Figure S4A). In summary, our results indicate that NIBAN2 acts as a key positive regulator, maintaining the stem‐like phenotype and functional state of GSCs.

### 2.3 NIBAN2 Interacts with FLII and Promotes Its Nuclear Translocation in GSCs

**FIGURE 3:**
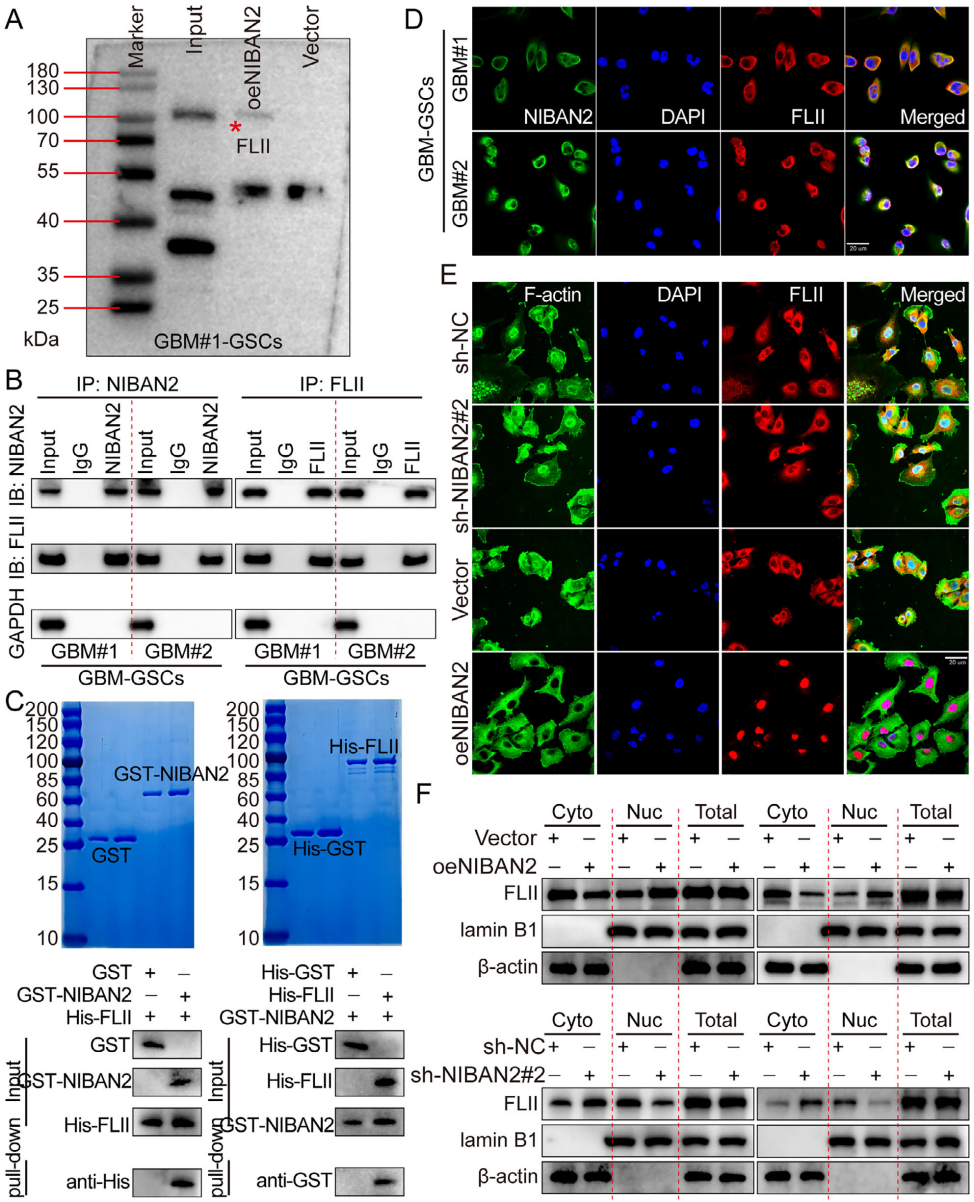
NIBAN2 interacts with FLII and promotes its nuclear translocation in GSCs. (A) MS analysis of proteins co‐immunoprecipitated with endogenous NIBAN2 in GSCs identified FLII as a top candidate interactor. (B) Co‐IP assays confirming the interaction between NIBAN2 and FLII in GSCs. Reciprocal IP using anti‐FLII antibody exhibited consistent results. (C) GST pull‐down assay indicating direct binding between recombinant GST‐tagged NIBAN2 and Flag‐tagged FLII in vitro. GST alone served as a negative control. (D) Representative IF images demonstrating subcellular localization of FLII in control, NIBAN2‐overexpressing, and NIBAN2‐knockdown GSCs. NIBAN2 overexpression promotes FLII nuclear translocation and colocalization (Pearson's coefficient = 0.82). Scale bars, 20 µm. (E) Representative IF images exhibiting the nuclear/cytoplasmic FLII distribution in GSCs with altered NIBAN2 expression. (F) Nuclear and cytoplasmic fractionation assays indicate that NIBAN2 overexpression increases FLII nuclear localization, while NIBAN2 knockdown reduces nuclear FLII levels. Lamin B1 and β‐actin served as markers for the nucleus and cytoplasm, respectively.

To elucidate the molecular mechanisms by which NIBAN2 regulates the stem‐like phenotype of GSCs, we first performed co‐immunoprecipitation (Co‐IP) coupled with mass spectrometry (MS) to identify potential NIBAN2‐interacting proteins (Figure 3A; Figure S4B). The results indicated that FLII was one of the most significantly enriched binding partners (Table S3). FLII is a multifunctional protein composed of gelsolin‐like and SANT domains, and is involved in cytoskeletal remodeling, transcriptional regulation, and chromatin remodeling [5, 6]. Recent studies have implicated FLII as a critical player in the development and progression of various cancers [7, 8, 9].

We first validated the interaction between NIBAN2 and FLII in GSCs using Co‐IP. The results demonstrated that NIBAN2 significantly co‐precipitated endogenous FLII, with this interaction being more stable in the stem‐like state of GSCs. Reciprocal Co‐IP using FLII antibodies exhibited consistent results (Figure 3B). To confirm direct binding, we constructed and purified a GST‐tagged NIBAN2 fusion protein and conducted GST pull‐down assays. In this study, we used an N‐terminal GST‐tagged expression vector (E. coli‐derived, pGEX‐4T). Because GST is fused to the N‐terminus of the NIBAN2 fragment, it is labeled as GST‐NIBAN2 (aa11‐351, N‐terminal GST fusion) in the figure. The recombinant GST‐NIBAN2 protein specifically enriched His‐tagged FLII, while no interaction was observed in the GST control group. This indicates a direct, co‐factor‐independent interaction between NIBAN2 and FLII in vitro (Figure 3C). Next, we examined the subcellular localization of both proteins using immunofluorescence (IF) co‐staining (Figure 3D). In addition, we performed immunofluorescence staining to assess the subcellular localization of FLII under conditions of NIBAN2 knockdown and overexpression. In control GSCs, FLII was predominantly localized to the cytoplasm. However, in NIBAN2‐overexpressing cells, FLII exhibited significant nuclear translocation and strong colocalization with NIBAN2. In contrast, NIBAN2 knockdown significantly impaired FLII nuclear localization, resulting in its cytoplasmic retention (Figure 3E). These findings were further confirmed by nuclear/cytoplasmic fractionation assays: NIBAN2 overexpression significantly increased FLII levels in the nuclear fraction, while NIBAN2 knockdown resulted in a significant reduction in nuclear FLII (Figure 3F). Collectively, these results indicate that NIBAN2 not only established a stable complex with FLII but also promotes its translocation from the cytoplasm to the nucleus‐an effect likely to be contributing to NIBAN2‐mediated maintenance of GSCs stemness.

To map the specific binding regions between NIBAN2 and FLII, we designed a series of deletion mutants. Based on the domain architecture, we constructed N‐terminal deletion (ΔN, aa566‐746), C‐terminal deletion (ΔC, aa1‐565), and full‐length (aa1‐746) constructs of NIBAN2, all of which were tagged with Myc for Co‐IP detection. Co‐transfection of these constructs with Flag‐tagged FLII into 293T cells, followed by Co‐IP, revealed that only NIBAN2‐ΔC (aa1‐565) retained the ability to bind FLII, indicating that the FLII‐binding domain resides in the C‐terminal region (aa1‐565) of NIBAN2. We then constructed a series of FLII truncation mutants based on its domain structure, including FLII‐Δ1 (aa1‐427), FLII‐Δ2 (aa428‐904), FLII‐Δ3 (aa905‐1269), FLII‐Δ4 (aa1‐904), and FLII‐Δ5 (aa428‐1269), each of which was fused with a Flag tag. These constructs were co‐transfected with Myc‐tagged NIBAN2 into 293T cells. Co‐IP experiments demonstrated that only FLII constructs containing the aa905‐1269 region retained binding to NIBAN2, indicating that the NIBAN2‐binding site is located within the C‐terminal region (aa905‐1269) of FLII (Figure S5A,B). We further analyzed the expression pattern and clinical relevance of FLII in glioma. TCGA database analysis indicated that FLII was significantly overexpressed in glioma tissues, and its expression was positively correlated with a poor patient prognosis (Figure S5C). This was validated by WB, qPCR, and IHC analyses on clinical glioma samples of different WHO grades. These experiments confirmed that FLII expression was significantly upregulated in glioma tissues compared to NBT and increased progressively with tumor grade (Figure S6A–C). Furthermore, NIBAN2 and FLII expression levels were positively correlated in both clinical specimens and TCGA datasets (Figure S6D,E), further supporting their potential cooperative role in glioma development and progression.

### 2.4 NIBAN2 Promotes the Malignant Phenotype of Glioma Stem Cells Through FLII

**FIGURE 4:**
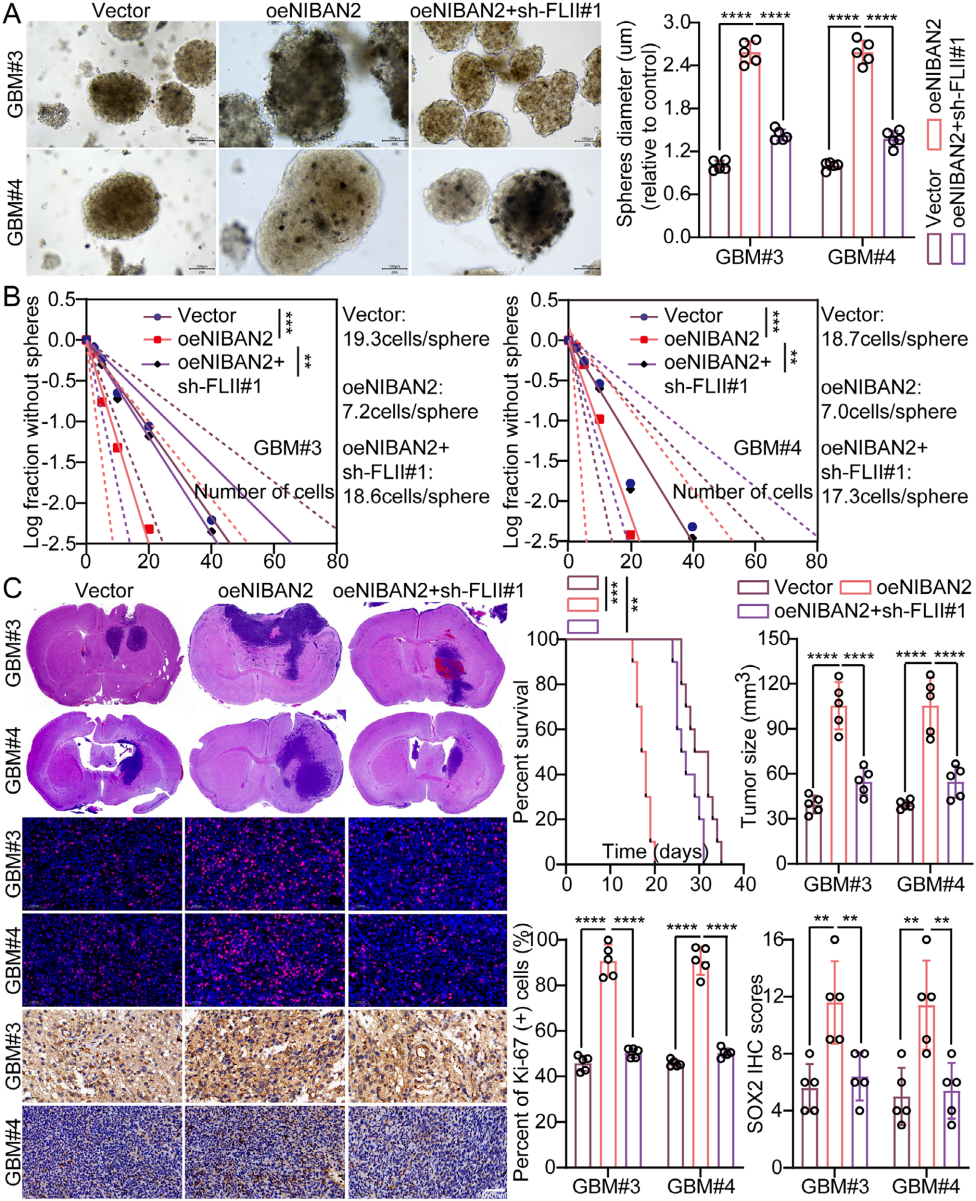
FLII is required for NIBAN2‐mediated stemness and tumorigenic potential of GSCs. (A) Tumorsphere formation assay in control, NIBAN2‐OE, and NIBAN2‐OE + shFLII GSCs. FLII knockdown attenuates the increase in sphere number and size induced by NIBAN2 overexpression. Scale bars, 100 µm. (B) LDA exhibiting stem cell frequency in the indicated groups, analyzed using the ELDA algorithm. FLII knockdown reverses the NIBAN2‐induced increase in stem‐like frequency. (C) Kaplan‐Meier survival curves of immunodeficient mice orthotopically implanted with GSCs expressing control vector, NIBAN2‐OE, or NIBAN2‐OE + sh‐FLII. Median survival was significantly reduced by NIBAN2 overexpression and rescued by FLII knockdown. IHC analysis of tumor tissues exhibiting SOX2 and Ki‐67 expression. Scale bars, 0.05 mm. Data were mean ± SD. Statistical significance was calculated by 1‐way ANOVA for A, B, and C; Survival analysis of C was performed by the log‐rank test. ^**^ *p* < 0.01, ^***^ *p* < 0.001, ^****^ *p* < 0.0001.

Our previous results demonstrated that NIBAN2 can directly bind to FLII and promote its nuclear translocation. To further determine whether the NIBAN2‐mediated enhancement of GSCs’ stemness and malignancy depends on FLII, we performed functional dependency assays. Specifically, we knocked down FLII in the context of NIBAN2 overexpression and determined changes in stemness‐related phenotypes. Tumor sphere formation assays revealed that FLII knockdown significantly attenuated the increased sphere‐forming capacity and size induced by NIBAN2 overexpression (Figure 4A). Besides, LDA indicated that FLII loss reduced the sphere formation frequency from 1/7.1 to 1/17.95 in NIBAN2‐overexpressing cells, approaching control levels (*p* < 0.01) (Figure 4B). In soft agar colony formation assays, NIBAN2 overexpression significantly increased the anchorage‐independent growth of GSCs, as evidenced by increased colony number and size. Conversely, FLII knockdown partially or completely abrogated this effect (∼60% reduction in colony number, *p* < 0.01) (Figure S7A). WB revealed that FLII knockdown suppressed the NIBAN2‐induced upregulation of stemness markers, including CD44, CD133, NANOG and Nestin (Figure S7B), further supporting their cooperative role in regulating the stemness program.

To further verify the in vivo functional role of NIBAN2‐FLII interaction in regulating GSCs’ malignancy, we established an orthotopic glioma xenograft model in immunodeficient mice. GSCs transduced with control vector, NIBAN2 overexpression (NIBAN2‐OE), or NIBAN2 overexpression plus FLII knockdown (NIBAN2‐OE + sh‐FLII) were injected into the mice brains, and tumor development and animal survival were observed. Kaplan‐Meier survival analysis revealed that mice in the NIBAN2‐OE group exhibited significantly reduced survival, with a median survival of 18 days compared to 31 days in the control group (*p* < 0.001). However, FLII knockdown significantly prolonged survival (median survival: 27 days, *p* < 0.01), nearly restoring it to control levels. Histological analysis revealed larger and more invasive tumors in the NIBAN2‐OE group, whereas FLII knockdown significantly suppressed tumor volume and invasion. Additionally, IHC and IF confirmed that SOX2 and Ki‐67 expression levels were significantly upregulated in the NIBAN2‐OE tumors; however, they were significantly downregulated upon FLII knockdown (Figure 4C). Collectively, these data demonstrate that NIBAN2 significantly enhances the in vivo tumorigenicity of GSCs. FLII knockdown can partially or fully reverse this effect, indicating that FLII is a critical downstream effector of NIBAN2's oncogenic function in vivo.

### 2.5 NIBAN2 Promotes FLII‐RREB1 Complex Formation and Enhances their Nuclear Interaction to Regulate the Stemness Program

To further investigate the molecular mechanism by which the NIBAN2‐FLII complex regulates GSCs’ stemness, we performed proteomic analysis of the NIBAN2 immunocomplex. Notably, in the context of FLII enrichment, the transcription factor RREB1 was highly co‐enriched, suggesting that it can act as a key downstream transcriptional effector of the complex. RREB1 is a classical zinc finger transcription factor known to play critical roles in cell fate determination, embryonic development, and stemness maintenance [10, 11, 12, 13].

**FIGURE 5:**
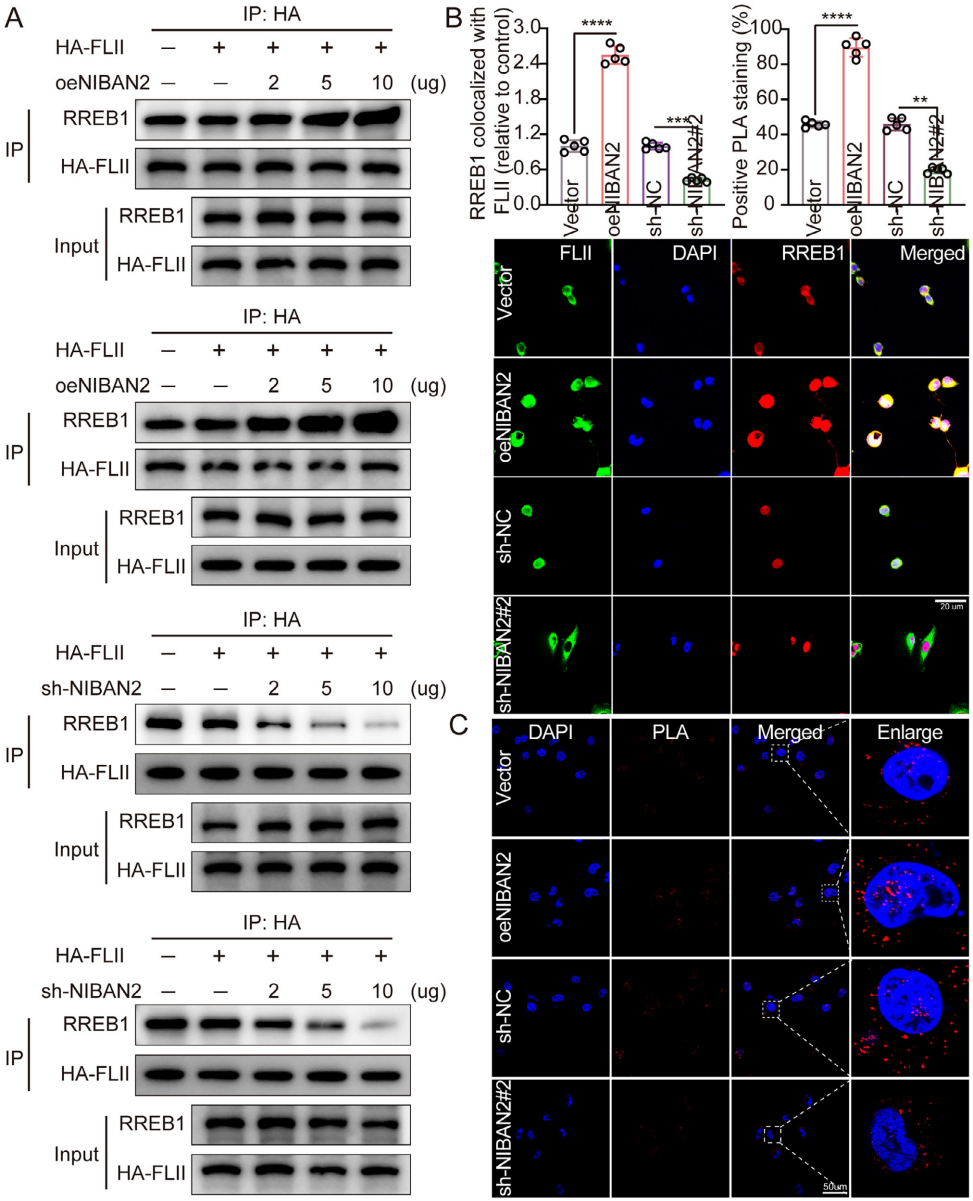
NIBAN2 facilitates FLII‐RREB1 complex formation and enhances their nuclear interaction in GSCs. (A) Co‐IP analysis of FLII‐RREB1 interaction in GSCs with NIBAN2 overexpression or knockdown. NIBAN2 expression positively correlates with FLII‐RREB1 complex formation in a dose‐dependent manner. (B) Representative IF images exhibiting subcellular colocalization of FLII and RREB1 in control, NIBAN2‐overexpressing, and NIBAN2‐knockdown GSCs. Pearson's correlation coefficients quantify nuclear colocalization. Scale bars, 20 µm. (C) PLA indicating enhanced nuclear interaction between FLII and RREB1 in NIBAN2‐overexpressing GSCs. Quantification of PLA signals per nucleus indicates a 2.3‐fold increase upon NIBAN2 overexpression. Scale bars, 50 µm. Data were mean ± SD. Statistical significance was calculated by 2‐way ANOVA for B. ^**^ *p* < 0.01, ^***^ *p* < 0.001, ^****^ *p* < 0.0001.

To investigate whether NIBAN2 modulates the interaction between FLII and RREB1, we generated NIBAN2 overexpression and knockdown models in GSCs and performed Co‐IP assays. The results revealed that NIBAN2 overexpression significantly increased the binding between FLII and RREB1 in a dose‐dependent manner, whereas NIBAN2 knockdown resulted in a dose‐dependent decrease in FLII‐RREB1 complex formation (Figure 5A), indicating that NIBAN2 facilitates their interaction. Furthermore, ternary Co‐IP assays indicated that NIBAN2 could simultaneously bind both FLII and RREB1, suggesting that it acts as a bridging factor to stabilize the tripartite complex. IF co‐staining exhibited significantly increased nuclear colocalization of FLII and RREB1 in NIBAN2‐overexpressing GSCs, whereas colocalization was significantly reduced in the NIBAN2 knockdown group (Figure 5B). To confirm whether NIBAN2 promotes nuclear translocation of FLII and enhances its spatial interaction with RREB1, we performed proximity ligation assay (PLA) at the cellular level. The results indicated that NIBAN2 overexpression significantly increased PLA signals between FLII and RREB1, predominantly localized in the nucleus. In contrast, PLA signals were significantly reduced upon NIBAN2 knockdown. Quantitative analysis of PLA foci indicated that NIBAN2 overexpression resulted in approximately a 2.3‐fold increase in nuclear PLA signals (*p* < 0.01) (Figure 5C). This finding further supported the role of NIBAN2 in facilitating FLII nuclear translocation and increasing its nuclear interaction with RREB1.

Additionally, analysis of TCGA datasets revealed that RREB1 was significantly upregulated in glioma tissues, and its high expression was positively correlated with poor patient prognosis (Figure S7C). To validate its expression profile, we performed WB, qPCR, and IHC on clinical glioma samples of varying grades. The results demonstrated that RREB1 expression was significantly upregulated in tumor tissues compared to NBT and increased progressively with tumor grade (Figure S8A–C). Furthermore, TCGA and clinical sample analyses exhibited a strong positive correlation between NIBAN2 and RREB1 expression (Figure S8D,E), further supporting their functional interplay in glioma progression.

### 2.6 RREB1 Knockdown Attenuates NIBAN2‐Induced Malignant Phenotypes in Glioma Stem Cells

**FIGURE 6:**
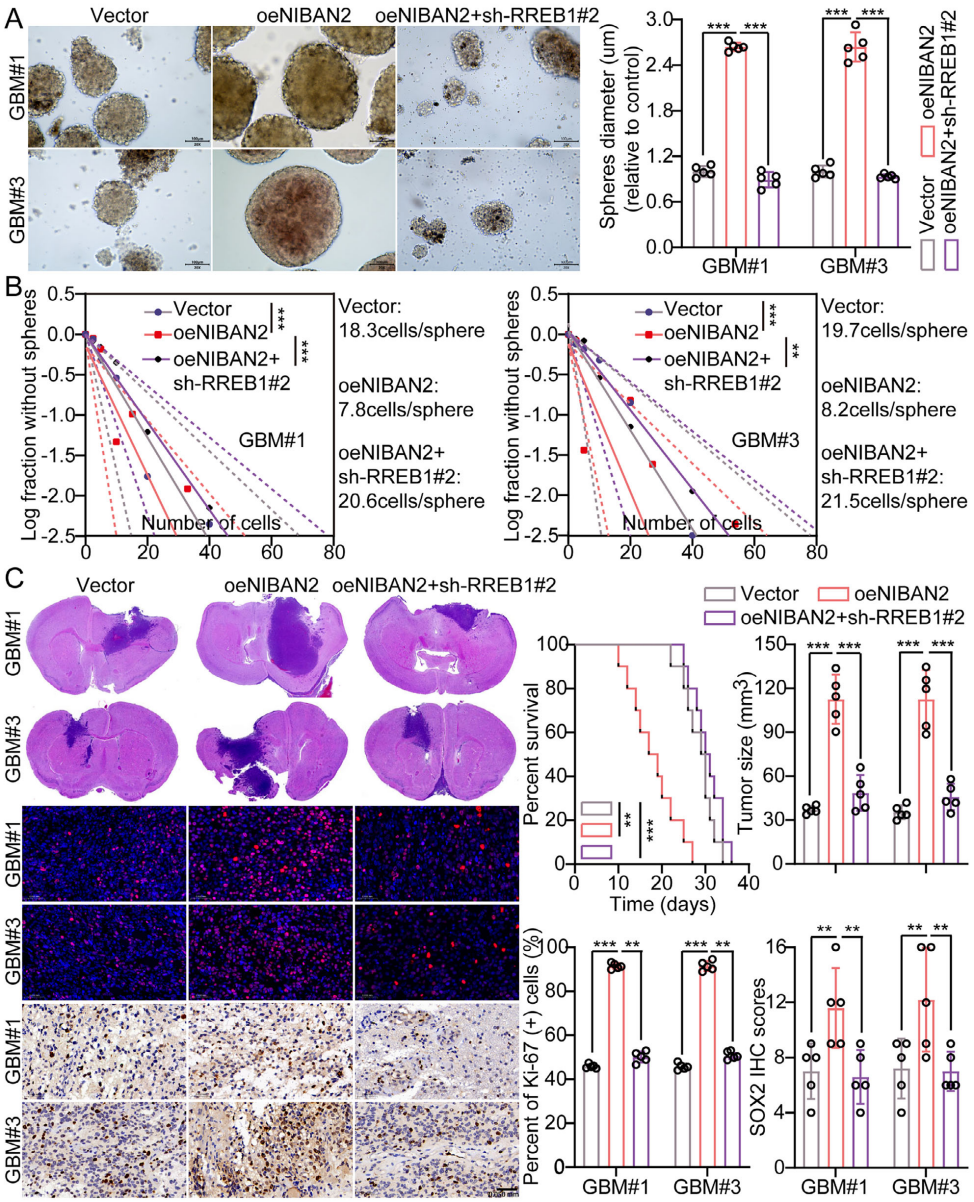
RREB1 is required for NIBAN2‐mediated stemness and tumorigenicity in GSCs. (A) Tumor sphere formation assay in GSCs transduced with control vector, NIBAN2‐OE, or NIBAN2‐OE + RREB1 knockdown (shRREB1). The sphere number and diameter were significantly reduced upon silencing of RREB1. Scale bars, 100 µm. (B) LDA indicating that the stem‐like cell frequency induced by NIBAN2‐OE (1/8.0) is significantly reduced upon RREB1 knockdown (1/21.1), approaching control levels. Stem cell frequency was calculated using the ELDA algorithm. (C) Kaplan‐Meier survival curves of immunodeficient mice orthotopically implanted with GSCs expressing control, NIBAN2‐OE, or NIBAN2‐OE + shRREB1. RREB1 knockdown significantly extended survival in NIBAN2‐overexpressing mice. Representative IHC staining of SOX2 and Ki‐67 expression in tumor tissues, exhibiting attenuation of NIBAN2‐induced stemness and proliferation upon RREB1 silencing. Scale bars, 0.05 mm. Data were mean ± SD. Statistical significance was calculated by 1‐way ANOVA for A, B, and C; Survival analysis of C was performed by the log‐rank test. ^**^ *p* < 0.01, ^***^ *p* < 0.001, ^****^ *p* < 0.0001.

Previous studies indicated that RREB1 interacts with the NIBAN2‐FLII complex to regulate the expression of stemness‐associated genes in GSCs. To determine whether RREB1 functions as a key downstream effector of NIBAN2‐driven malignant phenotypes, we performed functional antagonism experiments by knocking down RREB1 in the context of NIBAN2 overexpression. Tumor sphere formation assays revealed that RREB1 knockdown significantly impaired sphere‐forming capacity, with both sphere number and average diameter reduced by approximately 50% compared to NIBAN2 overexpression alone (Figure 6A). Furthermore, LDA exhibited a significant reduction in stem cell frequency from 1/8.0 to 1/21.1 (*p* < 0.001), approaching the baseline level observed in the control group (Figure 6B), indicating a dependency of NIBAN2‐induced self‐renewal on RREB1. Consistently, soft agar colony formation assays indicated that RREB1 knockdown significantly reduced the anchorage‐independent growth capacity conferred by NIBAN2, as evidenced by reduced colony number and area (Figure S9A). Moreover, WB analysis confirmed that RREB1 knockdown significantly reversed the NIBAN2‐induced upregulation of stemness markers, including CD44, CD133, NANOG, and Nestin (Figure S9B), supporting the hypothesis that RREB1 acts as a key terminal node in regulating the stemness program.

To validate the in vivo role of RREB1 as a downstream mediator of NIBAN2‐driven GSCs malignancy, we established an orthotopic intracranial glioma model in immunodeficient mice. GSCs transduced with control vector, NIBAN2‐OE, or NIBAN2‐OE combined with RREB1 knockdown (sh‐RREB1) were injected into mouse brains, followed by assessment of survival, tumorigenicity, and histopathology. Kaplan–Meier analysis revealed significantly shortened median survival in the NIBAN2‐OE group (17.5 days vs. 28.5 days in the control group, *p* < 0.01). In contrast, RREB1 knockdown significantly extended survival to 30.5 days (*p* < 0.001), approximately restoring it to control levels. Moreover, mice in the NIBAN2‐OE group exhibited higher tumor incidence and larger tumor volumes, which were significantly reduced by RREB1 knockdown. IHC and IF staining revealed that tumors from the NIBAN2‐OE group exhibited significantly elevated expression of stemness and proliferation markers, including SOX2 and Ki‐67. However, RREB1 knockdown significantly suppressed these markers and was accompanied by histological features suggestive of reduced malignancy, including more defined tumor boundaries and more compact cellular arrangement (Figure 6C).

In summary, these results identify RREB1 as not only a critical co‐regulator of NIBAN2‐mediated stemness phenotypes in vitro but also an essential downstream effector of NIBAN2‐driven glioma progression in vivo. Targeting RREB1 can offer a promising therapeutic strategy for suppressing the malignant progression of GSCs.

### 2.7 RREB1 Sustains GSCs Stemness by Transcriptionally Activating NIBAN2 and CD44

To investigate whether RREB1 maintains the stemness of GSCs through transcriptional regulation of NIBAN2 and CD44, we conducted a comprehensive set of experiments examining the regulation of expression, DNA‐binding capability, and functional dependency.

**FIGURE 7:**
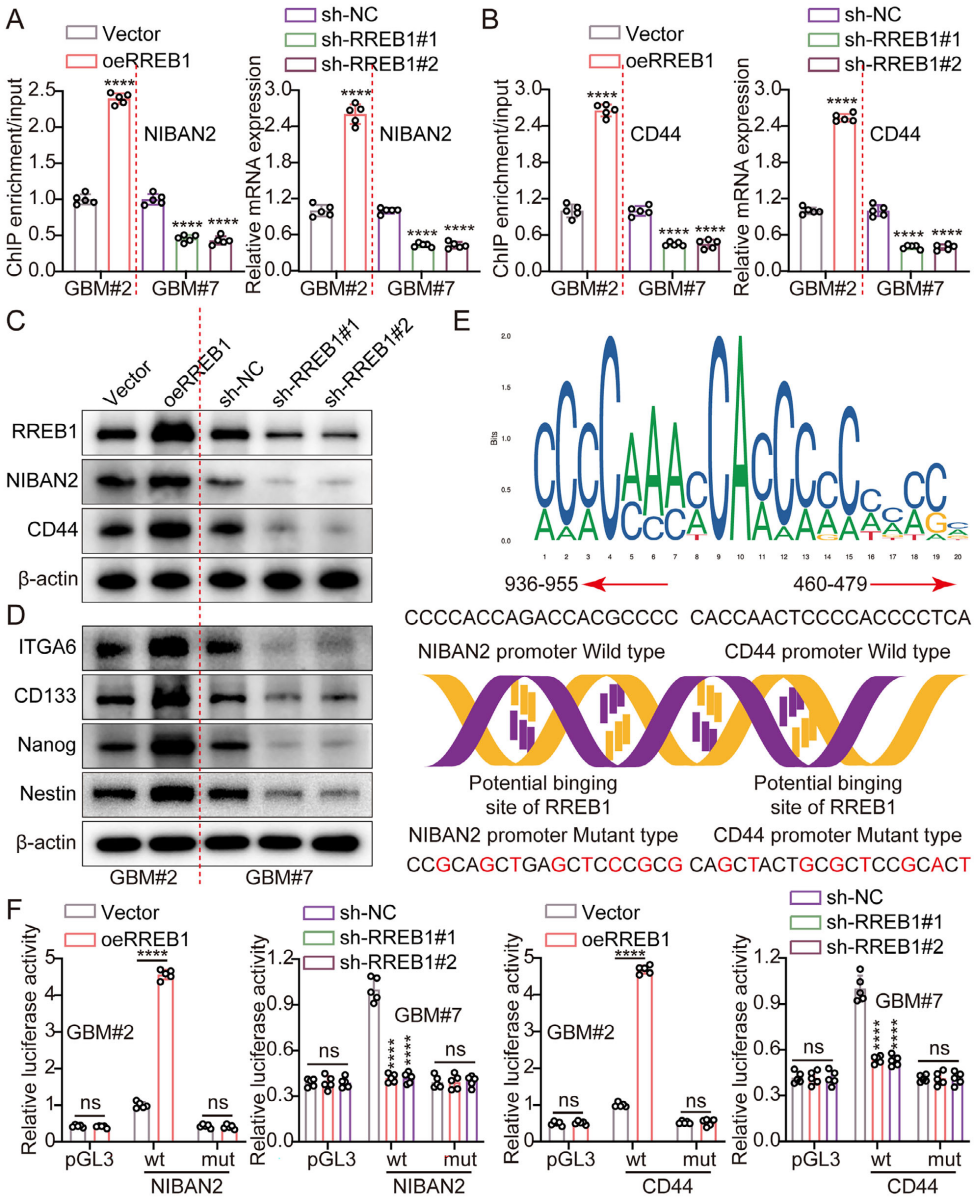
RREB1 transcriptionally activates NIBAN2 and CD44 to sustain stemness in GSCs. (A,B) ChIP‐qPCR exhibiting direct binding of RREB1 to RREs within the promoter regions of NIBAN2 and CD44. RREB1 overexpression increased enrichment at both loci. RT‐qPCR analysis demonstrating that RREB1 knockdown reduces, while overexpression increases, NIBAN2 and CD44 mRNA levels in GSCs. GAPDH was used as a loading control. (C,D) WB analysis indicating that RREB1 knockdown reduces, while overexpression increases, NIBAN2 and CD44 protein levels in GSCs. β‐actin was used as a loading control. (E) Predicted RREB1 binding sites within the −2 kb upstream regions of NIBAN2 and CD44 promoters, identified using JASPAR (MA0073.3) with a relative score threshold of 80%. (F) Dual‐luciferase reporter assay exhibiting RREB1‐induced transcriptional activation of NIBAN2 and CD44 promoters. Mutation of predicted RREB1 binding sites abolished this activation. Data were mean ± SD. Statistical significance was calculated by 2‐tailed unpaired Student's *t* tests and 1‐way ANOVA for A, B and F. ns, not significant, ^****^ *p* < 0.0001.

Chromatin immunoprecipitation (ChIP)‐qPCR analysis revealed that RREB1 directly binds to Ras‐responsive elements (RREs) located within the promoter regions of NIBAN2 and CD44. The binding enrichment of RREB1 at these sites was significantly increased in RREB1‐overexpressing cells, suggesting that RREB1 can directly recognize and activate these gene promoters. In addition, RT‐qPCR and WB analyses indicated that RREB1 knockdown significantly downregulated NIBAN2 and CD44 expression at both the mRNA and protein levels in GSCs, whereas RREB1 overexpression significantly upregulated their expression (Figure 7A–C). This finding supported a positive transcriptional regulatory role of RREB1 on both genes. Furthermore, RREB1 upregulated several other stemness‐associated markers, including Nestin, CD133, and NANOG (Figure 7D), further confirming its essential role in maintaining the stem‐like state of GSCs. To predict potential RREB1‐binding sites within the NIBAN2 and CD44 promoters, we used the JASPAR database [14]. and the human RREB1 binding motif (MA0073.3) to scan 2 kb upstream promoter regions. Using JASPAR's online scanning tool [14] with a relative score threshold of 80%, multiple high‐confidence putative binding sites were identified (Figure 7E). Dual‐luciferase reporters validated RREB1's ability to activate these promoter regions. RREB1 significantly increased luciferase activity driven by NIBAN2 or CD44 promoters, while mutation of the predicted RREB1‐binding sites abrogated this activation (Figure 7F), indicating that its transcriptional effect is sequence‐specific. Functionally, RREB1 overexpression promoted tumor sphere formation. However, knockdown of either NIBAN2 or CD44 partially reversed these stemness‐promoting effects (Figure S9C), suggesting that the stemness maintenance function of RREB1 is at least partly dependent on the expression of these two target genes. Consistently, soft agar colony formation assays revealed that silencing NIBAN2 or CD44 attenuated the clonogenic advantage conferred by RREB1 overexpression (Figure S9D).

In conclusion, RREB1 directly binds to and transcriptionally activates the promoters of NIBAN2 and CD44, thereby promoting their expression and driving the stemness program in GSCs. These findings establish RREB1 as a central transcriptional regulator in the glioma stemness regulatory network.

### 2.8 RREB1 Drives Aerobic Glycolysis and Sustains Metabolic Stemness in GSCs by Transcriptionally Regulating LDHA

RREB1 is a zinc finger transcription factor that mediates Ras signaling responses and plays critical roles in tumorigenesis and metabolic regulation across various cancers [15, 16, 17]. Given that LDHA is a key rate‐limiting enzyme in the aerobic glycolysis pathway [18, 19, 20], we hypothesized that RREB1 can sustain the metabolic stemness of GSCs by transcriptionally activating LDHA expression.

**FIGURE 8:**
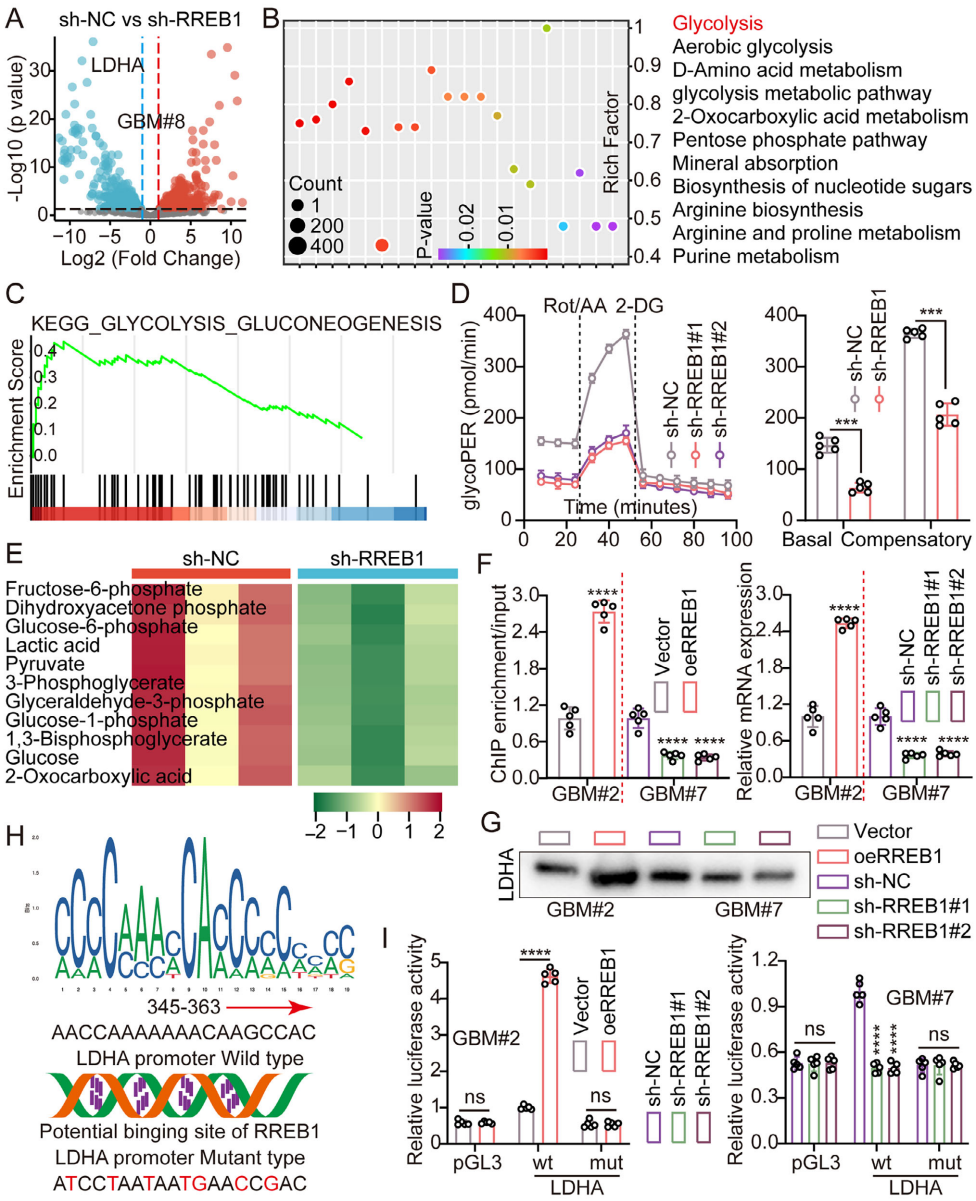
RREB1 transcriptionally activates LDHA to promote aerobic glycolysis and maintain metabolic stemness in GSCs. (A) Volcano plot demonstrating differentially expressed genes in RREB1‐knockdown GSCs based on RNA‐seq analysis. LDHA is among the most significantly downregulated targets (log_2_FC = −1.7, adjusted *p* < 0.001). (B) KEGG pathway enrichment analysis of downregulated genes, highlighting glycolysis/gluconeogenesis as a top‐enriched pathway. (C) GSEA exhibiting significant suppression of glycolysis gene signatures upon RREB1 knockdown. (D) Seahorse ECAR analysis indicating decreased basal and compensatory glycolysis in RREB1‐deficient GSCs. (E) Targeted metabolomics profiling of glycolytic intermediates (including glucose‐1‐phosphate, fructose‐6‐phosphate, 3‐phosphoglycerate, and phosphoenolpyruvate) exhibiting reduced abundance following RREB1 knockdown. (F) ChIP‐qPCR confirming RREB1 binding to the LDHA promoter at RREs. Binding was enriched in cells overexpressing RREB1. (G) RT‐qPCR and WB analyses revealed that RREB1 positively regulates LDHA expression at both mRNA and protein levels. GAPDH served as a loading control. (H) Predicted RREB1 binding motifs within the −2 kb upstream region of the LDHA promoter, identified using the JASPAR database (MA0073.3) with a relative score threshold of 80%. (I) Dual‐luciferase reporter assay demonstrating that RREB1 overexpression increases transcriptional activity of the LDHA promoter. Mutation of predicted RREB1 binding sites significantly attenuated this activation, indicating sequence‐specific regulation. Data were mean ± SD. Statistical significance was calculated by 2‐tailed unpaired Student's *t* tests for D; 2‐tailed unpaired Student's *t* tests and 1‐way ANOVA for F and I. ns, not significant, ^***^ *p* < 0.001, ^****^ *p* < 0.0001.

To confirm this hypothesis, we performed RNA sequencing on GSCs with RREB1 knockdown. The results exhibited global downregulation of glycolysis‐related genes, with LDHA exhibiting the most significant suppression (log_2_FC = −1.7, adjusted *p* < 0.001), ranking in the top 1% of all differentially expressed genes. Volcano plots and heatmaps confirmed LDHA's high sensitivity to RREB1 regulation, suggesting it as a potential direct target (Figure 8A; Figure S10A,B). Kyoto encyclopedia of genes and genomes (KEGG) pathway enrichment analysis of significantly downregulated genes identified glycolysis as one of the top‐ranked pathways, consistently enriched across various cellular contexts (Figure 8B; Figure S10C). Gene set enrichment analysis (GSEA) further demonstrated that the loss of RREB1 resulted in a significant downregulation of hallmark glycolysis gene clusters (Figure 8C). Functionally, seahorse extracellular flux analysis indicated that RREB1 knockdown significantly reduced both basal and compensatory glycolytic rates (Figure 8D). Targeted metabolomics further revealed significant reductions in key glycolytic intermediates, including glucose‐1‐phosphate, fructose‐6‐phosphate, 3‐phosphoglycerate, and phosphoenolpyruvate, in RREB1‐deficient cells (Figure 8E). Additionally, expression of multiple glycolytic enzyme‐coding genes was significantly downregulated at the transcriptional level (Figure S10D), suggesting that RREB1 serves as an upstream transcriptional regulator of the glycolytic program. To determine whether RREB1 directly regulates LDHA transcription, we performed ChIP‐qPCR, which revealed that RREB1 binds to RREs in the LDHA promoter region, with significantly enhanced enrichment under RREB1 overexpression. RT‐qPCR and WB analyses confirmed that RREB1 knockdown significantly suppressed LDHA mRNA and protein levels, whereas overexpression of RREB1 elevated LDHA expression (Figure 8F,G). Sequence scanning of the 2 kb upstream region of the LDHA promoter using the RREB1 motif (MA0073.3) from the JASPAR database [14]. identified several high‐confidence binding sites (Figure 8H). Besides, dual‐luciferase reporter assays validated that RREB1 increases the transcriptional activity of the LDHA promoter, whereas mutation of key binding sites significantly impaired this effect, indicating sequence‐specific activation (Figure 8I). Together, these results indicate that RREB1 transcriptionally activates LDHA by directly binding to its promoter, thereby promoting aerobic glycolytic flux and maintaining the metabolic homeostasis and stemness of GSCs. This identifies RREB1 as a key upstream regulator in the glycolysis‐stemness axis.

TLR signaling has been implicated in maintaining the stemness of cancer cells [21, 22, 23]. To explore potential downstream pathways mediated by NIBAN2 in GSCs, we performed GSEA comparing NIBAN2‐overexpressing and control cells. The results exhibited significant enrichment of the TLR signaling pathway in the NIBAN2‐overexpressing group (NES = 1.86, FDR < 0.01) (Figure S10E), suggesting its potential role as a key downstream axis of NIBAN2. Leading‐edge analysis further identified upregulation of key components in this pathway, including TLR3, MYD88, and IRAK1, in response to NIBAN2 overexpression. WB analysis confirmed these trends, with NIBAN2 overexpression increasing the protein levels of TLR3, MYD88, and IRAK1, as well as p65 phosphorylation, indicating activation of the canonical NF‐κB pathway. Conversely, NIBAN2 knockdown suppressed the expression of these molecules (Figure S10F), confirming NIBAN2 as a key activator of TLR3 signaling.

To evaluate the clinical relevance of these pathway components, we analyzed TCGA glioma RNA‐seq data and associated survival information. TLR3, MYD88, and IRAK1 were significantly upregulated in glioma tissues compared to normal brain (Figure S10G; Figures S11 and S12). Kaplan–Meier survival analysis revealed that high expression of these genes was strongly associated with a poor prognosis (Figures S11A and S12A,C). Pearson's correlation analysis revealed a positive correlation between NIBAN2 expression and TLR3, MYD88, and IRAK1 at the transcriptional level (Figures S11B and S12B,D and S14A), suggesting that NIBAN2 can promote glioma progression by co‐activating the TLR3 signaling cascade. Notably, TLR signaling, particularly through the TLR3/NF‐κB axis, has been reported to transcriptionally regulate stemness‐related transcription factors, including SOX2, OCT4, and NANOG, thereby increasing the stemness of tumor cells. In addition, to further substantiate the functional role of TLR3 signaling in NIBAN2‐mediated maintenance of GSC stemness and malignant progression, we simultaneously knocked down TLR3 in NIBAN2‐overexpressing GSCs. We found that TLR3 inhibition markedly reduced the expression of downstream components MYD88 and IRAK1, as well as the level of p65 phosphorylation. Concomitantly, the sphere‐forming capacity of GSCs and the expression of self‐renewal‐associated stemness markers (including SOX2, NESTIN, and CD133) were significantly impaired. Furthermore, to validate the contribution of TLR3 signaling in NIBAN2‐deficient cells, we re‐expressed TLR3 in NIBAN2‐depleted GSCs. These rescue experiments showed that TLR3 overexpression partially restored GSC self‐renewal capacity, stemness marker expression, and tumorsphere formation in NIBAN2‐deficient cells (Figure S13A–C). In parallel, we performed Western blotting to assess how perturbing individual nodes within this circuitry (e.g., LDHA inhibition or NF‐κB blockade) affects RREB1 and NIBAN2 expression. These analyses showed that suppressing LDHA or blocking NF‐κB conversely reduced the protein levels of both RREB1 and NIBAN2 (Figure S13D). Collectively, these results provide additional evidence supporting a functional role of TLR3 signaling downstream of NIBAN2.

In conclusion, our findings suggest that NIBAN2 can activate the canonical TLR3 pathway to promote the stemness of GSCs and drive malignant glioma progression. This provides new mechanistic insights into the signaling network underlying NIBAN2‐mediated stemness regulation in glioma.

### 2.9 Combined Inhibition of NIBAN2 and LDHA Induces Synergistic Anti‐Tumor Effects in NIBAN2‐Positive Glioma Models

To identify potential small‐molecule inhibitors targeting NIBAN2, we performed structure‐based virtual screening. Using Schrödinger software, we modeled the human NIBAN2 protein (UniProt ID: Q96TA1, 746 amino acids) based on the crystal structure (PDB ID: 7CTP) and predicted potential ligand‐binding pockets using the Sitemap module. The highest‐scoring pocket, site 1, was selected as the docking region. After protein preparation and grid generation, we conducted a three‐tiered molecular docking of 3158 Federal Drug Authority (FDA)‐approved compounds from the L1010 library using HTVS, SP, and XP precision modes, followed by MM‐GBSA rescoring based on binding free energy (ΔG_bind). A total of 59 compounds exhibited ΔG_bind values below −30 kcal/mol, with the top three candidates being demecarium bromide (−68.28 kcal/mol), travoprost (−65.17 kcal/mol), and nelfinavir (−64.52 kcal/mol). Docking results revealed that these compounds stably occupy the site 1 pocket through hydrogen bonding, π‐cation interactions, and aromatic stacking (Figures S14B–D and S15A,B), supporting their potential for functional inhibition.

**FIGURE 9:**
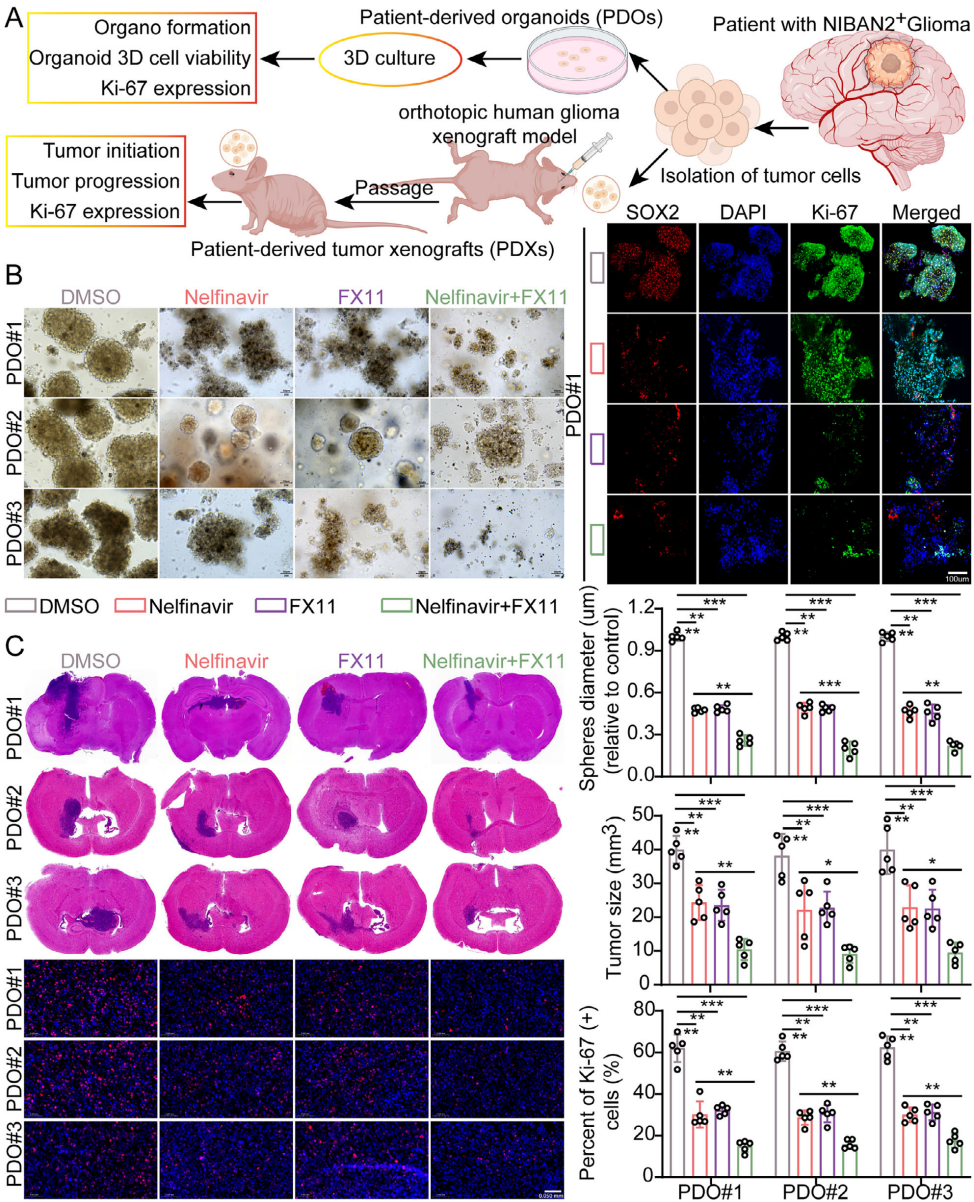
Combined inhibition of NIBAN2 and LDHA induces synthetic lethality in NIBAN2‐high glioblastoma models. (A,B) Viability of PDOs treated with NIBAN2 inhibitor (nelfinavir), LDHA inhibitor (FX11), or their combination. The organoid viability was determined after five days of treatment. Combination therapy significantly reduced PDO viability compared to either monotherapy. Representative IF images of SOX2, Ki‐67, and TUNEL staining in treated PDOs. Combined treatment significantly decreased stemness and proliferation markers, while promoting apoptosis. Scale bars, 100 µm. (C) In vivo efficacy of combined treatment with nelfinavir and FX11 in NIBAN2‐high PDX glioblastoma models. Tumor volume was significantly reduced after dual treatment compared to single agents. Representative IHC images exhibit decreased NIBAN2, LDHA, and Ki‐67 expression, along with increased TUNEL‐positive cells, in tumors from the combination group. Scale bars, 0.05 mm. Data were mean ± SD. Statistical significance was calculated by 2‐way ANOVA for **C**. ^***^ *p* < 0.001, ^****^ *p* < 0.0001.

To compare their interference effects on the NIBAN2‐FLII complex, we treated GSCs with demecarium bromide, travoprost, or nelfinavir and performed Co‐IP assays. NIBAN2‐FLII interaction remained stable in the DMSO, demecarium, and travoprost groups; however, nelfinavir significantly weakened their association, as indicated by significantly reduced band intensity (Figures S15C and S16A). This suggested that nelfinavir selectively disrupts NIBAN2‐FLII binding and can serve as a functional small‐molecule inhibitor. To evaluate the therapeutic potential of dual targeting NIBAN2 and LDHA, we used patient‐derived organoid (PDO) models generated from glioma patients with high NIBAN2 expression. PDOs were treated with nelfinavir (an NIBAN2 inhibitor), FX11 (an LDHA inhibitor), or a combination of both. While monotherapies moderately reduced PDO viability (∼30%–40%), combination treatment reduced viability by over 80% (*p* < 0.001), indicating strong synergistic toxicity (Figure 9A,B). Apoptosis surveys exhibited increased Caspase‐3/7 activity and Annexin V positivity in the combination group (Figure S16B). Additionally, IF staining revealed decreased SOX2 and Ki‐67 expression, as well as increased TUNEL‐positive cell ratios (Figure 9B; S16C). To validate this in vivo, we established PDX models using NIBAN2‐high glioma samples in immunodeficient mice. Combined treatment with nelfinavir and FX11 significantly suppressed tumor growth, reducing tumor volume by over 75% compared to monotherapy (*p* < 0.001) (Figure 9C), without affecting mouse body weight, indicating good tolerability (Figure S16D). Kaplan‐Meier analysis revealed that median survival was extended from 22 to 45 days in the combination group (*p* = 0.0004) (Figure S16E). IF exhibited downregulated NIBAN2 and LDHA expression, decreased Ki‐67 positivity, and increased TUNEL‐positive cells in the combination group (Figure 9C; Figure S16F). In addition, we systematically compared the subcellular localization, complex assembly, and protein abundance of FLII‐RREB1 in paired parental GBM cells and their corresponding GSCs. The results showed that, relative to parental GBM cells, the FLII‐RREB1 complex exhibited a more prominent nuclear enrichment in GSCs: nuclear/cytoplasmic fractionation followed by Western blotting revealed a marked increase in the nuclear complex signal with a concomitant reduction in the cytoplasmic fraction (Figure S17A–C). Quantitatively, both the nuclear FLII‐RREB1 Co‐IP signal and the number of nuclear complexes were significantly higher in GSCs than in parental GBM cells (p < 0.05). Meanwhile, the total protein levels of FLII and RREB1 were only modestly elevated in GSCs (∼1.2–1.5‐fold), a magnitude far smaller than the pronounced upregulation of NIBAN2. Importantly, the changes in nuclear/cytoplasmic distribution and complex assembly were substantially stronger than the changes in total protein abundance. Collectively, these data indicate that GSCs not only upregulate NIBAN2 but also preferentially assemble FLII‐RREB1 into a complex that accumulates in the nucleus, thereby supporting activation of stemness‐associated transcriptional programs. This is consistent with our mechanistic model in which NIBAN2 promotes nuclear enrichment of the FLII‐RREB1 complex to sustain the stem‐like phenotype. In parallel, we performed a proximity ligation assay (PLA) coupled with confocal imaging to interrogate FLII‐RREB1 complex localization (DMSO control vs. nelfinavir treatment; nuclei counterstained with DAPI), followed by quantitative analysis of nuclear vs. cytoplasmic PLA puncta distribution. In the control group, FLII‐RREB1 PLA puncta were predominantly enriched within the nucleus, indicating a pronounced nuclear localization of the complex. Upon nelfinavir treatment, nuclear FLII‐RREB1 PLA signals were markedly reduced, accompanied by an attenuated nuclear enrichment and a redistribution toward the cytoplasm. Consistently, quantification of PLA puncta per nucleus and/or the nuclear‐to‐cytoplasmic PLA ratio revealed a significant decrease in the nelfinavir group compared with controls (*p* < 0.05) (Figure S17D).

To further substantiate the synergistic anti‐tumor efficacy of nelfinavir and FX11 in PDO models with high NIBAN2 expression, we stratified the PDO cohort according to NIBAN2 levels. This analysis revealed that, under a high‐NIBAN2 context, the combination regimen produced a more pronounced synergistic tumor‐suppressive effect (Figure S18). Of course, metabolomic analysis revealed decreased lactate levels and a significant reduction in extracellular acidification rate (ECAR), indicating effective suppression of glycolysis (Figure S19A and Table S4).

In summary, NIBAN2 and LDHA collaboratively regulate metabolic homeostasis in glioma. Their combined inhibition induces potent synthetic lethality in NIBAN2‐positive PDO and PDX models, underscoring the translational potential of targeting metabolic vulnerabilities in glioma therapy.

### 2.10 NIBAN2/FLII/RREB1 Axis is Co‐Expressed in Clinical Glioma Samples and is Significantly Associated with Poor Prognosis

**FIGURE 10:**
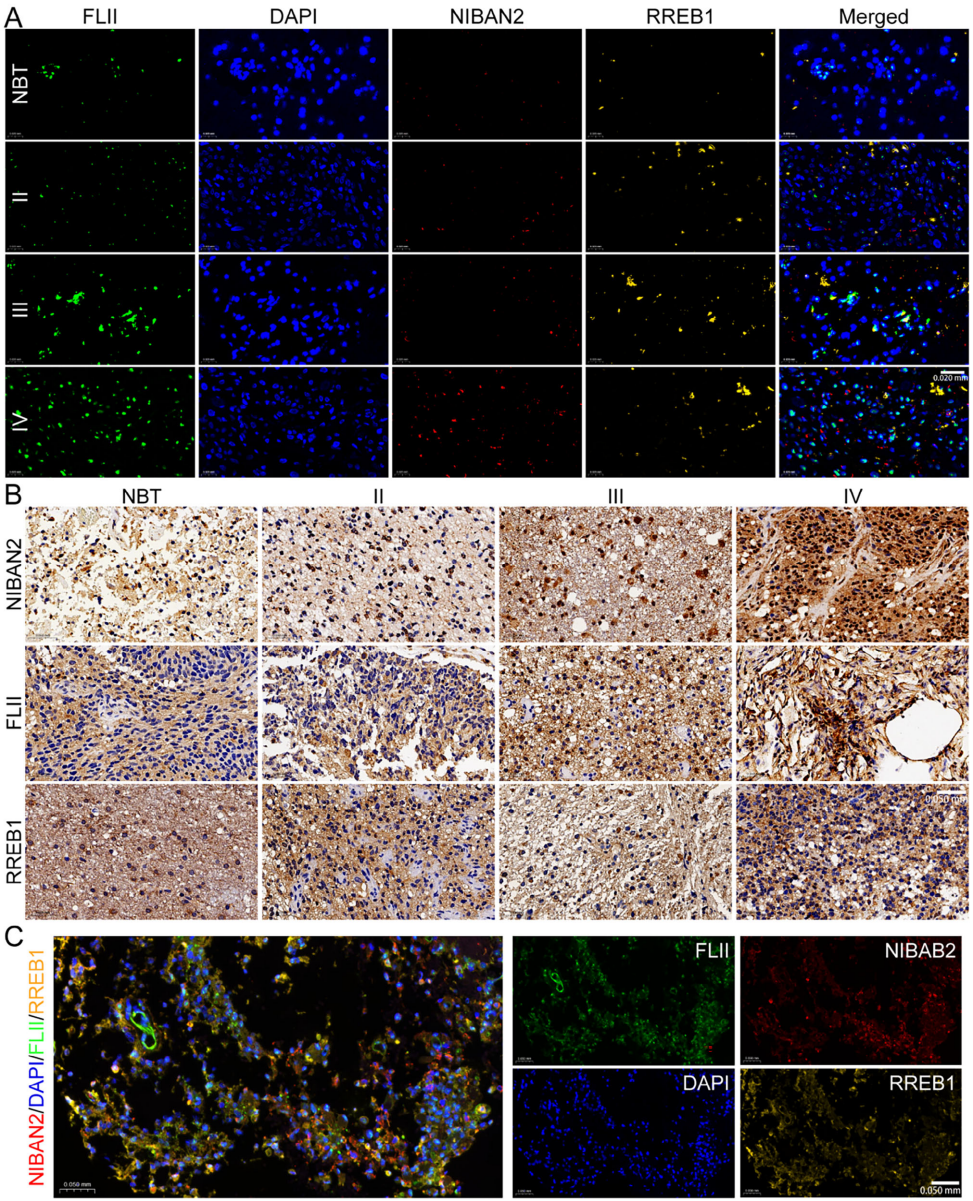
Coordinated expression of the NIBAN2/FLII/RREB1 positive feedback loop in clinical glioma samples correlates with a poor prognosis. (A) IF analysis of NIBAN2, FLII, and RREB1 expression in normal brain, LGG, primary GBM, and recurrent GBM tissues (*n*  =  118). Representative images are presented. Scale bars, 0.02 mm. (B) Spearman's correlation analysis exhibiting strong positive correlations among NIBAN2, FLII, and RREB1 expression across glioma tissues (*r*  =  0.71‐0.84, *p* < 0.001). (C) Representative TMA images indicating co‐expression of NIBAN2, FLII, and RREB1 in GBM samples, with enrichment in regions of high Ki‐67 or SOX2 expression. Scale bars, 0.05 mm.

To determine the expression status of the NIBAN2/FLII/RREB1 axis in clinical glioma tissues, we collected 118 specimens across different WHO grades, including 10 primary GBM, 54 LGG, 10 recurrent GBM, and 20 NBT. IF and IHC analyses exhibited consistently upregulated expression of NIBAN2, FLII, and RREB1 in GBM tissues, significantly exceeding levels observed in LGG and normal brain samples (Figure 10A,B). Multiplex immunofluorescence imaging revealed the co‐expression of NIBAN2, FLII, and RREB1 in GBM specimens (Figure 10C). Stratified statistical analysis revealed that the frequency of triple‐high expression increased with tumor grade: in GBM, NIBAN2, FLII, and RREB1 exhibited positive rates of 82%, 76%, and 78%, respectively, whereas their expression rates in LGG were all below 30%. Molecular subtype analysis revealed that the loop was more highly expressed in patients with unmethylated MGMT promoter and IDH wild‐type status (Figures S19B and S20A). Histopathological examination revealed that patients with co‐expression of the loop components frequently exhibited features of malignancy, including tumor necrosis, increased angiogenesis, and diffusely infiltrative margins (Figure S20B). Kaplan–Meier survival analysis indicated that high expression of all three components was significantly associated with shorter progression‐free survival and overall survival (log‐rank test, *p* < 0.01) (Figure S20C), suggesting that loop activation can serve as a negative prognostic biomarker. Additionally, dual‐channel IF exhibited significant nuclear colocalization of NIBAN2 and FLII in SOX2‐positive stem‐like regions, along with upregulated nuclear RREB1 expression. These markers exhibited a significant spatial overlap in GSCs‐enriched areas (Figure S20D), providing histological evidence of coordinated spatial activation.

In addition, we re‐analyzed the publicly available single‐cell RNA‐seq dataset GSE138794, with a specific focus on wild‐type IDH human glioblastoma samples (GSM4119531, GSM4119532, GSM4119533, and GSM4119534). Following the original authors’ cell‐type annotations and retaining only high‐quality cells for clustering and annotation, we found that FAM129B (also known as NIBAN2) displayed a higher average expression in tumor cells than in immune cells across these patient specimens. Notably, upon isolating tumor cells and performing further subclustering, NIBAN2 expression was elevated in subpopulations exhibiting more pronounced stem‐like features, indirectly supporting a potential functional heterogeneity of NIBAN2 within glioma tissues. Nevertheless, these observations should be interpreted with caution. On the one hand, glioblastoma is intrinsically characterized by marked intra‐ and inter‐tumoral heterogeneity, and our analysis is limited to a small number of publicly available wild‐type IDH samples (*n* = 4), which may not capture the full spectrum of molecular subtypes or clinical diversity. On the other hand, we also observed relatively high NIBAN2 expression in endothelial cells within the same dataset, suggesting that its functional roles may extend beyond tumor‐intrinsic programs and potentially involve vascular‐related processes in the tumor microenvironment. Therefore, while these findings provide preliminary insights into the potential relevance of NIBAN2 in glioma, the generalizability of its expression pattern and its functional significance warrant further validation in larger and more diverse single‐cell and/or spatial transcriptomic cohorts (Figure S21).

Collectively, these findings suggest that the NIBAN2/FLII/RREB1 axis is highly co‐expressed in clinical glioma tissues, with strong correlations to tumor grade, molecular subtype, and poor clinical outcome. These results further support the pathogenic significance of this axis in glioma progression, highlighting its potential as a prognostic biomarker and therapeutic target. (Figuer 11)

**FIGURE 11:**
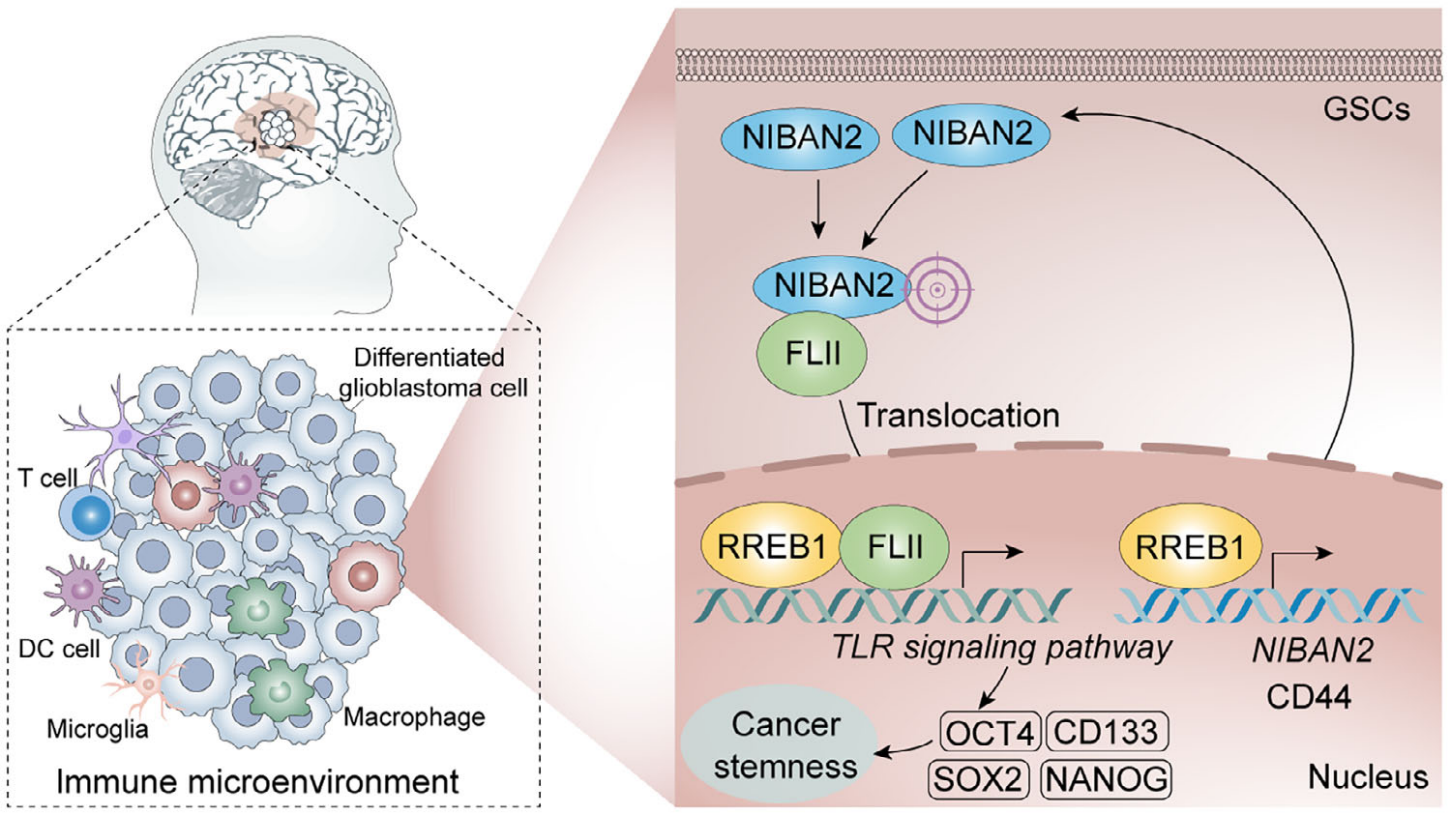
Schematic model illustrating the mechanism by which NIBAN2 promotes GSCs maintenance and glioblastoma progression. NIBAN2, highly expressed in glioma stem‐like cells (GSCs), assembles with FLII and transcription factor RREB1 to form a nuclear complex. This complex transcriptionally activates stemness‐associated genes (e.g., CD44, NANOG) and metabolic enzymes (e.g., LDHA), thereby sustaining both transcriptional and metabolic stemness programs. Moreover, RREB1 further enhances NIBAN2 expression, establishing a positive feedback loop. Concurrently, NIBAN2 activates the TLR4/NF‐κB pathway, promoting inflammatory signaling and glioma malignancy. Pharmacological co‐inhibition of NIBAN2 and LDHA elicits a synthetic lethality effect in NIBAN2‐high glioma models, highlighting a potential therapeutic strategy targeting metabolic vulnerability in glioblastoma.

## 3 Discussion

This study systematically identifies NIBAN2 (also known as FAM129B or MINERVA) as a central regulatory factor in glioma genesis and the maintenance of glioma stemness. NIBAN2 is highly expressed in GSCs, and functional studies reveal that it assembles a FLII‐RREB1 transcriptional complex that cooperatively improves the stem‐like properties and malignant behavior of GSCs. Mechanistically, NIBAN2 not only activates the TLR3 signaling pathway to sustain stemness‐related transcriptional programs but also induces expression of the transcription factor RREB1, which in turn upregulates LDHA. This process drives aerobic glycolysis and establishes a “signaling‐transcription‐metabolism” triadic coupling network that supports the homeostasis of GSCs (Figures 1, 2, 3, 4, 5, 6, 7, 8, 11).

Currently, we have mainly demonstrated, through ChIP‐qPCR and luciferase assays, that RREB1 and FLII co‐occupy the promoter regions of NIBAN2, CD44, and LDHA, enhancing the transcriptional activity of these target genes. However, we have not yet systematically proven the “necessity and sufficiency” of RREB1 binding or the “directness” of FLII's role in RREB1 chromatin occupancy through dual perturbation of RREB1/FLII, DNA‐binding defective mutants, or genome‐wide occupancy profiling. Additionally, other cofactors may be involved in the regulation at different target gene loci. Therefore, I can only state that “the RREB1‐FLII complex is involved in the transcriptional regulation of NIBAN2, CD44, and LDHA, but at specific gene loci, it may rely on additional cofactors”.

The functions of NIBAN2, a member of the FAM129 protein family, a group of cancer‐associated molecules, are not yet fully understood. Early studies implicated NIBAN2 in cellular stress response, apoptosis resistance, and signal transduction [24, 25, 26]. Schmidlin CJ and colleagues reported that FAM129B‐dependent activation of NRF2 promotes an invasive phenotype in BRAF‐mutant melanoma cells. Chen ZM and colleagues reported that NIBAN2 stimulates glioma growth by modulating the JAK2/STAT3/c‐Myc signaling axis. Our findings demonstrate, for the first time, that NIBAN2 robustly promotes the tumor sphere formation and self‐renewal capacity of GSCs. This is primarily achieved by promoting the nuclear translocation of its interacting partner FLII, which in turn strengthens the FLII‐RREB1 interaction and induces the expression of stemness‐related genes, including *CD44*, *SOX2*, and *NANOG*. FLII is a conserved bifunctional protein containing gelsolin‐like domains (involved in actin remodeling) and leucine‐rich repeats (for signal transduction and protein interaction). It is involved in cytoskeletal remodeling, transcriptional regulation, embryonic development, and tissue regeneration [5, 27]. FLII is aberrantly expressed in various cancers, contributes to epithelial‐mesenchymal transition, and is implicated in neurodevelopmental disorders [7, 8, 28]. In our study, the nuclear translocation of FLII was crucial for NIBAN2‐mediated maintenance of stemness. FLII knockdown effectively reversed the stemness phenotype induced by NIBAN2 and extended mouse survival in GSCs orthotopic implantation models, establishing FLII as a critical downstream effector.

RREB1, a zinc‐finger transcription factor that is downstream of the Ras pathway, is essential for stem cell homeostasis, metabolic reprogramming, and immune evasion [10, 15, 29]. In this study, RREB1 emerged as a key node in the NIBAN2 pathway; its knockdown significantly impaired GSCs’ stemness and prolonged animal survival. Moreover, RREB1 transcriptionally activates *NIBAN2* and its downstream target *CD44*, and upregulates *LDHA*, forming a positive feedback loop that promotes glycolytic flux. ChIP and luciferase assays confirmed the direct binding of RREB1 to the promoter regions of these targets. At the metabolic level, RREB1 knockdown resulted in significant downregulation of LDHA expression, ECAR, and lactate production, supporting its central role in GSCs glycolytic reprogramming. LDHA is a critical enzyme in aerobic glycolysis, contributing to energy production and immune suppression in highly proliferative tumor cells [30, 31, 32]. Concurrently, NIBAN2 overexpression significantly upregulated TLR3, MYD88, TRAF6, and phosphorylated p65, indicating activation of the TLR‐NF‐κB axis. Beyond its role in innate immunity [33, 34], TLR3 signaling has been implicated in reinforcing cancer stemness by promoting transcription of stemness genes, suppressing differentiation, and increasing therapy resistance [35, 36, 37]. Moreover, TCGA analysis exhibited high co‐expression of *NIBAN2* with TLR4, RREB1, and LDHA in glioma samples, all of which are associated with poor clinical outcomes (Figures S9–S11).

From a therapeutic perspective, structure‐based virtual screening identified the FDA‐approved drug nelfinavir as a high‐affinity NIBAN2 binder. Co‐IP assays validated its ability to disrupt the NIBAN2‐FLII complex. Notably, combined treatment with nelfinavir and the LDHA inhibitor FX11 in NIBAN2‐high glioma PDOs and PDX models exhibited a synergistic anti‐tumor effects, promoting apoptosis and glycolysis inhibition. This combination demonstrated strong synergy and benefits of survival. Histological analyses revealed downregulated expression of SOX2 and Ki‐67, along with an increase in TUNEL‐positive apoptotic cells, in the combination group, indicating the effective depletion of stem‐like and proliferative GSC populations (Figure 9; Figures S13, and S14).

Nelfinavir has been reported to induce ER stress, inhibit proteasomal function, and regulate the PI3K/AKT signaling pathway. In this study, we can only demonstrate that nelfinavir functionally interferes with the NIBAN2‐FLII complex in our model. Based on the current evidence, nelfinavir can be considered a pharmacological intervention targeting the NIBAN2‐FLII axis, but it should not be referred to as a “specific” or “selective disruptor.” This is because we have not yet conducted biophysical experiments such as SPR, ITC, CETSA, or thermal shift assays to directly prove the binding of nelfinavir to NIBAN2, nor have we systematically evaluated its structural analogs or negative control compounds. Furthermore, the GSC inhibitory effects observed in this study are likely the result of a multi‐target action, with the NIBAN2‐FLII‐LDHA axis being an important target that we have focused on through virtual screening and functional experiments. However, we cannot rule out the synergistic contribution of other pathways.

In summary, this study proposes a novel regulatory model of glioma stemness, wherein NIBAN2 promotes assembly of the FLII‐RREB1 complex, co‐activates TLR3 signaling and LDHA‐mediated glycolysis, and stabilizes both the transcriptional and metabolic programs of GSCs. RREB1 serves as a central hub that not only regulates glycolytic enzymes but also maintains NIBAN2 expression, forming a feed‐forward signaling–transcription–metabolism axis. The co‐targeting strategy against NIBAN2 and LDHA induces synergistic anti‐tumor effects, providing a new therapeutic avenue focused on the “metabolism‐stemness” network. Future studies should further investigate the applicability and specificity of this mechanism across various glioma molecular subtypes, immune microenvironments, and therapeutic resistance landscapes, thereby establishing the foundation for clinical translation.

## 4 Materials and Methods

### 4.1 Clinical Samples

Tumor specimens were collected from glioma patients undergoing surgical resection at Wuhan Union Hospital (Wuhan, China). Clinical and demographic characteristics of the cohort are provided in Table S1. All procedures involving human participants adhered to the Declaration of Helsinki and the International Ethical Guidelines for Biomedical Research Involving Human Subjects (CIOMS) [38]. Written informed consent was obtained from all participants. The study protocol was approved by the Ethics Committee of Wuhan Union Hospital (approval no. S0775‐01). Each specimen was divided into three parts for: (i) formalin fixation and paraffin embedding, (ii) protein and RNA extraction, and (iii) establishment of primary glioma cell cultures.

### 4.2 Cell Culture and Treatment

HEK‐293T cells (human embryonic kidney 293T cells) were obtained from the American Type Culture Collection (ATCC, Manassas, VA, USA) and cultured in Dulbecco's modified Eagle medium (DMEM; Gibco, Waltham, MA, USA) supplemented with 10% fetal bovine serum (FBS; Gibco), glutamine (Gibco), and 1% penicillin‐streptomycin (Gibco), under standard conditions of 5% CO_2_ at 37°C. The isolation, culture, and characterization of all GBM primary cells and GSCs were performed as described previously [39, 40, 41, 42]. Briefly, tumor tissues were collected intraoperatively, dissociated into single‐cell suspensions, and cultured in Iscove's modified Dulbecco's medium (IMDM) supplemented with 20% FBS (Gibco, Waltham, MA, USA), 1% penicillin‐streptomycin (Gibco), and 1% sodium pyruvate. All cell lines underwent short tandem repeat profiling and were routinely tested for mycoplasma contamination.

### 4.3 Plasmids

Full‐length cDNAs of *NIBAN2*, *FLII*, and *RREB1* were obtained from Wuhan Gene Create Biological Engineering Co., Ltd. The sequences were verified before subcloning into either pcDNA3.3 or pLVX‐Puro expression vectors (Clontech). Truncated variants of *NIBAN2* and *FLII* were constructed by PCR using the respective pcDNA3.3 constructs as templates and subsequently cloned into the pcDNA3.3 vector. Besides, wild‐type cDNAs were subcloned into the pGEX‐4T‐1 vector to construct GST‐tagged *FLII* and *RREB1* fusion constructs. Site‐directed mutants of *NIBAN2* and *FLII* were obtained from Wuhan Gene Create Biological Engineering Co., Ltd. Primer sequences are listed in Table S2. Additional details are provided in our previously published studies [39, 40, 43].

### 4.4 shRNA‐Knockdown, Single‐Guide RNA (sgRNA)‐Knockout, and Transfection Assays

shRNA‐mediated knockdown, CRISPR/Cas9‐mediated knockout, and transfection assays were performed as previously described. Single‐guide RNA (sgRNA) sequences targeting *NIBAN2*, *FLII*, and *RREB1* were designed using the MIT CRISPR design tool (http://crispr.mit.edu). shRNA sequences were obtained from Gene Create Biological Engineering Co., Ltd (Wuhan, China). A complete list of siRNA and shRNA sequences is provided in Table S3. Additional experimental details are available in our previously published work [41, 44, 45].

### 4.5 WB and Immunoprecipitation (IP) Essays

WB and IP assays were performed as previously described [41, 43, 46]. Cells were lysed in IP lysis buffer supplemented with a complete protease inhibitor cocktail (Roche) and incubated on ice for 30 min. Lysates were centrifuged and then subjected to SDS‐PAGE, followed by immunoblotting with the indicated antibodies. Detailed protocols are provided in the Supporting Information and our previously published article. A complete list of antibodies is presented in Table S4.

### 4.6 Tumor Sphere Formation Assay

Single‐cell suspensions were prepared by enzymatic dissociation and then passed through a 40 µm cell strainer to ensure a single‐cell state. Cells were seeded into ultra‐low attachment plates (Corning) at a density of 1000–5000 cells/ml in serum‐free DMEM/F12 medium supplemented with B27 (minus vitamin A, Thermo Fisher), 20 ng/ml EGF, 20 ng/ml bFGF, and 2 µg/ml heparin. Cultures were incubated at 37°C in a humidified 5% CO_2_ incubator for 7–10 days without disturbance. Primary spheres (> 50 µm in diameter) were quantified under an inverted microscope. For secondary sphere formation, primary spheres were dissociated into single cells and replated under identical conditions. Sphere‐forming efficiency was calculated as the number of spheres formed divided by the number of cells seeded.

### 4.7 Limiting Dilution Assay (LDA)

For in vitro limiting dilution analysis, single‐cell suspensions were seeded into ultra‐low attachment 96‐well plates (Corning) at graded densities (1, 5, 10, 20, and 50 cells per well), with 12–24 replicate wells per condition. Cells were cultured in serum‐free DMEM/F12 medium supplemented with B27, 20 ng/ml EGF, 20 ng/ml bFGF, and 2 µg/ml heparin. After 7–10 days of incubation under standard sphere‐forming conditions, each well was scored for the presence or absence of sphere formation (≥ 50 µm in diameter). The sphere‐forming frequency was calculated using the ELDA software (http://bioinf.wehi.edu.au/software/elda/).

### 4.8 Soft Agar Colony Formation Assay

Anchorage‐independent growth was determined using a soft agar assay. A base layer of 0.5% low‐melting‐point agarose in complete culture medium was prepared in 6‐well plates and allowed to solidify. Single‐cell suspensions were mixed with 0.3% agarose in complete medium and seeded on top of the base layer (2,000‐5,000 cells per well). Cells were incubated at 37°C in a humidified incubator with medium added every 3–4 days. After 2–3 weeks, colonies were stained with crystal violet, and colonies larger than 50 µm were counted under an inverted microscope. The quantification was performed using ImageJ software.

### 4.9 Immunofluorescence (IF) and Immunohistochemistry (IHC) Staining

IF and IHC staining were performed as previously described [44, 47, 48]. Briefly, human or mouse tissue samples were fixed in 4% paraformaldehyde, embedded in paraffin, and sectioned for subsequent immunostaining. For IF staining, sections were fixed in 4% paraformaldehyde for 15 min at room temperature, permeabilized with 0.5% Triton X‐100 for 10 min, and blocked with 5% bovine serum albumin (BSA) for 1 h to prevent non‐specific binding. Sections were then incubated with fluorophore‐conjugated secondary antibodies (1:200, Thermo Fisher Scientific), and nuclei were counterstained with DAPI (C1002, Beyotime). Fluorescence images were acquired using a Nexcope NE930 fluorescence microscope (Ningbo, China). A complete list of primary antibodies is provided in Table S4.

### 4.10 Dual‐Luciferase Reporter Assay

HEK293T cells were co‐transfected with pGL3 constructs containing wild‐type (WT), mut1 (−700 to −300 bp deletion), or mut2 (−100 bp truncation) promoter regions of NIBAN2, RREB1, or LDHA, together with wild‐type or mutant expression plasmids for NIBAN2, RREB1, or LDHA, or empty vector controls. Primary GBM cells and GSCs with control or NIBAN2/RREB1/LDHA knockdown were transfected with the same pGL3‐promoter constructs (WT, mut1, or mut2). Cells were harvested 48 h post‐transfection. Luciferase activity was measured using the Dual‐Luciferase Reporter Assay Kit (TransGen, FR201‐01‐V2) according to the manufacturer's instructions. Firefly and Renilla luciferase activities were quantified using the SYNERGY microplate reader (BioTek) and normalized to determine promoter activity.

### 4.11 Glycolytic Metabolism Assay

Glycolytic activity was evaluated by measuring glucose consumption, lactate production, and ATP generation. Glucose, lactate, and ATP levels were quantified using the Glucose Assay Kit (ADS‐W‐TDX002, AIDISHENG, Jiangsu, China), Lactate Assay Kit (ADS‐W‐T009‐96, AIDISHENG), and ATP Assay Kit (A095‐2‐1, Nanjing Jiancheng Bioengineering Institute), respectively, according to the manufacturers’ instructions. Briefly, cells were transfected with plasmids for 48 h or treated with DMSO or the indicated compounds, followed by measurement of ATP, glucose, and lactate concentrations in the culture medium.

### 4.12 Extracellular Acidification Rate (ECAR) Measurement

ECAR was determined using the Seahorse XF Glycolysis Stress Test Kit (103020‐100, Agilent) on a Seahorse XFe24 Analyzer (Agilent Technologies) according to the manufacturer's protocol. Briefly, transfected or control human primary glioblastoma cells or GSCs were seeded into Seahorse XFp cell culture microplates, washed, and incubated in Seahorse XF base medium at 37°C for 1 h. Measurements were performed in technical triplicate, and ECAR values were normalized to total protein content.

### 4.13 Chromatin Immunoprecipitation (ChIP)

ChIP assays were performed using the Magna ChIP A/G Chromatin Immunoprecipitation Kit (Millipore) according to the manufacturer's protocol. Briefly, 1 × 10^6^ cells were cross‐linked with 1% formaldehyde at 37°C for 10 min. Nuclei were isolated and digested with micrococcal nuclease, followed by sonication of nuclear lysates to generate chromatin fragments ranging from 150 to 900 bp. Samples were vortexed every 3 min during the procedure to ensure consistent shearing. For immunoprecipitation, 2 µg of the indicated antibodies, RREB1 (Proteintech, 20280‐1‐AP), or RNA polymerase II (Abcam, ab238146) were added to each lysate and incubated at 4°C for 4 h. Chromatin‐protein complexes were captured with protein G magnetic beads, eluted, and reverse cross‐linked. DNA was purified and subjected to qPCR.

### 4.14 Bioinformatic Analysis

FPKM‐normalized RNA‐sequencing data and corresponding clinical information for glioma patients were obtained from the TCGA database (https://cancergenome.nih.gov/). RNA‐sequencing data from NBT were retrieved from the Genotype‐Tissue Expression (GTEx) project. Survival analyses were performed using the Kaplan‐Meier method, with group differences determined by the log‐rank test. Comparisons between two groups were performed using unpaired *t*‐tests, and results were reported as *t*‐values. Correlations between variables were evaluated using Pearson correlation coefficients. Statistical significance was defined as *p* < 0.05, with significance levels denoted as follows: ns (not significant), *p* ≤ 0.05 (^*^), p ≤ 0.01 (^**^), p ≤ 0.001 (^***^).

### 4.15 In Vivo Xenograft Experiments

Pathogen‐free female BALB/c mice (6–8 weeks old) were obtained from Beijing Vital River Laboratory Animal Technology Co., Ltd. Mice were housed in a specific pathogen‐free facility and acclimated for one week before experimentation. For each independent experiment, 5–8 mice were assigned per group. Primary glioblastoma cells or GSCs were suspended in 100 µl PBS at a concentration of 8 × 10^6^ cells per mouse and stereotactically injected into the cerebral cortex. Cells were administered either untreated or after specific pre‐treatments.

For therapeutic intervention, tumor‐bearing mice received intraperitoneal injections of nelfinavir (12 mg/kg), FX11 (15 mg/kg), or a combination of both every other day, starting on day eight after tumor implantation. Drug stock solutions were aliquoted and stored at −80°C. Working solutions were freshly prepared in PBS before each administration. On day 23 of treatment, D‐luciferin was administered, and tumor progression was assessed by bioluminescence imaging (BLI) using the IVIS 100 system (Bruker MS FX Pro, Bruker, USA). Survival was monitored thereafter using independently treated cohorts under identical experimental conditions. Mice were observed daily and euthanized following humane endpoints approved by the Institutional Animal Care and Use Committee at the Tongji Medical College. Survival curves were analyzed using the log‐rank (Mantel‐Cox) test. All experiments were repeated independently with consistent results. Animal procedures were conducted in compliance with protocols approved by the Animal Care and Use Committee of Tongji Medical College.

### 4.16 Statistical Analysis

Statistical analyses were performed using the Statistical Package for Social Sciences software (SPSS version 25.0, SPSS Inc., Chicago, IL, USA). Data normality was determined using the Shapiro‐Wilk test (α = 0.05). For comparisons between two groups, unpaired or paired two‐tailed *t*‐tests were used for normally distributed data with equal variance; otherwise, corrected *t*‐tests or the Mann‐Whitney U test were applied. To compare three or more groups, one‐way analysis of variance (ANOVA), Brown‐Forsythe ANOVA, or the Kruskal‐Wallis test was used as appropriate. Post hoc multiple comparisons were adjusted using the Dunnett T3 or Dunn's test to control the Type I error rate. Data are presented as mean ± standard deviation (SD) or median with interquartile range, as indicated. Statistical significance was defined as a two‐tailed *p* < 0.05. The detailed statistical results for all *p* values and confidence intervals reported in the manuscript are provided in Table S5.

## Author Contributions

M.W., X.Q., Y.H., J.Y., X.J., and J.L. conceived and designed the study. L.S., Y.H., and J.L. performed the experiments. Data analysis was conducted by M.W., X.Q., Y.H., S.Y., Z.G., H.Z., Y.R., L.L., R.F., T.L., and X.J. M.W., X.Q., and Y.H. wrote the manuscript, and J.Y., X.J., and J.L. revised it. All authors read and approved of the final manuscript. J.L. serves as the guarantor and takes responsibility for the integrity of the work.

## Ethics Approval and Consent to participate

All human studies were approved by the Ethics Committee of Wuhan Union Hospital (approval no. S0775‐01), and written informed consent was obtained from all participants. All animal experiments were conducted in accordance with institutional guidelines and approved by the Institutional Animal Care and Use Committee of Tongji Medical College (IACUC no. 4701).

## Consent for Publication

All authors have reviewed and approved the final version of the manuscript for publication.

## Conflicts of Interest

The authors declare no conflicts of interest.

## Supporting information




**Supporting File 1**: advs74572‐sup‐0001‐SuppMat.docx.

**Supporting File 2**: advs74572‐sup‐0002‐Supplementary Tables.zip.

## Data Availability

The datasets generated and/or analyzed during the current study are available from the corresponding author upon reasonable request.
