# Supplementary material for: NIBAN2/FLII/RREB1 Axis Drives Glioma Stem Cell Malignancy via TLR3 Pathway Activation
Source: Adv Sci (Weinh). 2026 Feb 25;13(26):e19382. doi: 10.1002/advs.202519382 (PMC13159147; doi:10.1002/advs.202519382)
Supplement: Supplementary file 1 — Supporting File 1: advs74572‐sup‐0001‐SuppMat.docx. [file ADVS-13-e19382-s001.docx]

**A NIBAN2/FLII/RREB1 Positive Feedback Loop Drives the Malignant Phenotype of Glioma Stem Cells through TLR Pathway Activation**

Liang liang Shi^1*^, Xinwei Qiao^2*^, Yue Hu^1*^, Minjie Wang^3*^,Shaojie Yu^3^, Zihan Gong^3^, Yuxin Rao^3^, Hui Zheng^4^, Siting Chen^5^, Lin Li^3^, Rong Fu^3^, Tao Liang^6^, Jin Yao^1#^, Xiaobing Jiang^3#^, Junjun Li^3,7#^

**Supplementary Methods**

**WB and immunoprecipitation (IP) assays**

Cells were lysed in immunoprecipitation (IP) buffer supplemented with a complete protease inhibitor cocktail. After centrifugation, the supernatants were incubated with the indicated primary antibodies overnight at 4 °C on a rotating shaker. The immune complexes were then captured using Pierce™ Protein A/G Magnetic Beads (Invitrogen) for 1 h at room temperature with gentle agitation. Beads were washed thoroughly five times with wash buffer to remove nonspecifically bound proteins. Bound proteins were eluted by heating in 1× SDS loading buffer and subjected to immunoblotting. To identify NIBAN2-interacting proteins, approximately 6 × 10⁸ GSCs stably overexpressing either NIBAN2 or GFP (control) were harvested and lysed in IP buffer containing protease inhibitors. Equal amounts of lysate were immunoprecipitated overnight at 4 °C with anti-NIBAN2 antibody and Protein A/G Magnetic Beads. After extensive washing, bound proteins were eluted at room temperature for 10 min in elution buffer with continuous mixing. The eluates were separated from the beads magnetically, neutralized, and immediately boiled. Samples were resolved by SDS-PAGE and visualized by silver staining. Gel bands were excised and subjected to LC-MS/MS analysis for protein identification. Detailed antibody information is provided in Supplementary Table 4.

**Supplementary Table S1:** Clinical Characteristics of Primary GBM specimens.

| Specimen | Diagnosis | Age/Sex | Pathology report |
| --- | --- | --- | --- |
| GBM#1 | GBM | 62/F | GFAP(+),S-100(+),Olig2(+), P53(60%+), ATRX(-),PCK(-),IDH-1(R132H)(-),Ki67(Li：20%） |
| GBM#2 | GBM | 58/M | GFAP(+),S-100(+),Olig2(+), P53(<5%),ATRX(+), IDH1(R132H)(-),H3K27Me3(+),H3K27M(-),Ki67(LI:30%） |
| GBM#3 | GBM | 61/M | GFAP(+),S-100(+),Olig2(+), ATRX(+), IDH-1(R132H)(-), P53(15%+),NF(-),CD34(-),Ki67(Li: 15%） |
| GBM#4 | GBM | 69/F | GFAP(+),S-100(+),Olig2(+), NF(-),EMA(+),SOX10(-),ATRX(+),IDH1(R132H)(-),P53(30%+),Ki67(Li:30%） |
| GBM#5 | GBM | 35/F | GFAP(+),S-100(+),ATRX(+),Olig2(-),P53(-),IDH-1(R132H)(-),Ki67(LI: 20%) |
| GBM#6 | GBM | 66/M | GFAP(+),S-100(+),Olig2(-),PCK(-),IDH-1(R132H)(-), ATRX(+), P53(+80%), BRAF(V600E)(-),H3K27M(-),H3K27Me3(+)，Ki67(LI:10%) |
| GBM#7 | GBM | 61/M | GFAP(+),S-100(+),Olig2(+),P53(15%+),ATRX(+),IDH-1(R132H)(-), Ki67(Li:20%),CD34(-) |
| GBM#8 | GBM | 70/F | GFAP(+),S-100(+),Olig2(+), P53(40%),ATRX(+),NF(-)，IDH-1(R132H)(-)，Ki67(Li:30%） |

**Supplementary Table S2:** The used sequences of qRT-PCR.

| **Gene** | **Primer** | **Sequence(5′-3′)** |
| --- | --- | --- |
| NIBAN2 | forward | TCACGTCCGTGGACCAATAC |
|  | reverse | CGCGCATAAGGATGCCAGA |
| FLII | forward | CTCAGGCATTGGCAAGCTCA |
|  | reverse | GACCTCGATCTCCGTCAGG |
| RREB1 | forward | AGGTTCAGACCTATCTTCCATCA |
|  | reverse | CTGCCAATCCGATTTGGTCCT |
| β-actin | forward | ATTGCCGACAGGATGCAGAA |
|  | reverse | GCTGATCCACATCTGCTGGAA |
| GAPDH | forward | GAGTCAACGGATTTGGTCGT |
|  | reverse | TTGATTTTGGAGGGATCTCG |

**Supplementary Table S3:** The used sequences of shRNAs and siRNAs.

| **Gene** | **Primer** | **Sequence(5′-3′)** |
| --- | --- | --- |
| sh-NIBAN2#1 | forward | GCAGAGCTGCTATGAGAAGAT |
| sh-NIBAN2#2 | forward | GTCCCTTCTTTGGATGTCCTT |
| sh-FLII#1 | forward | GACATCATGTTGCTAGACAAT |
| sh-FLII#2 | forward | CCATTTCAAGAGGAAGTTCAT |
| sh-RREB1#1 | forward | AGGAGTTTGTTTGCAAGTATG |
| sh-RREB1#2 | forward | TAGGAAGTTTCCTCGCATTTC |
| sh-CD44#1 | forward | GCCCTATTAGTGATTTCCAAA |
| sh-CD44#2 | forward | CCTCCCAGTATGACACATATT |

**Supplementary Table S4:** The related antibodies involved in this experiment.

| Name | Description |
| --- | --- |
| Anti-FAM129B antibody | 22553-1-AP (Proteintech, Wuhan, China ) |
| Anti-FLII antibody | 67039-1-Ig (Proteintech, Wuhan, China ) |
| Anti-RREB1 antibody | 84048-1-RR (Proteintech, Wuhan, China ) |
| Anti-CD133 antibody | ab222782 (Abcam, Cambridge, USA) |
| Anti-CD44 antibody | ab254530 (Abcam, Cambridge, USA) |
| [Anti-Nanog antibody](https://www.abcam.cn/products/primary-antibodies/bnip3lnix-antibody-epr4033-ab109414.html) | ab109250 (Abcam, Cambridge, USA) |
| Anti-Nestin antibody | ab105389 (Abcam, Cambridge, USA) |
| Anti-GST antibody | RPT0001 (ABclonal, Wuhan, China ) |
| Anti-GAPDH antibody | #5174 (Cell Signaling Technology, Beverly, MA, USA) |
| Anti-β-actin antibody | 81115-1-RR (Proteintech, Wuhan, China ) |
| Anti-His antibody | AE086 (ABclonal, Wuhan, China ) |
| Anti-Lamin B1 antibody | ab194109 (Abcam, Cambridge, USA) |
| [Anti-ITGA6 antibody](https://www.abcam.cn/products/primary-antibodies/mitofusin-2-antibody-epr19796-ab205236.html) | ab181551 (Abcam, Cambridge, USA) |
| [Anti-LDHA antibody](https://www.abcam.cn/products/primary-antibodies/sod2mnsod-antibody-epr2560y-ab68155.html) | 19987-1-AP (Proteintech, Wuhan, China ) |
| [Anti-MYC antibody](https://www.abcam.cn/products/primary-antibodies/sod2mnsod-antibody-epr2560y-ab68155.html) | 60003-2-Ig (Proteintech, Wuhan, China ) |
| [Anti-Flag antibody](https://www.abcam.cn/products/primary-antibodies/cox-iv-antibody-epr9442abc-mitochondrial-loading-control-ab202554.html) | 66008-4-Ig (Proteintech, Wuhan, China ) |
| [Anti-TLR3 antibody](https://www.abcam.cn/products/primary-antibodies/cox-iv-antibody-epr9442abc-mitochondrial-loading-control-ab202554.html) | ab307442 (Abcam, Cambridge, USA) |
| [Anti-MyD88 antibody](https://www.abcam.cn/products/primary-antibodies/tomm20-antibody-epr15581-54-mitochondrial-marker-ab186735.html) | ab133739 (Abcam, Cambridge, USA) |
| [Anti-IRAK-1 antibody](https://www.abcam.cn/products/primary-antibodies/tomm20-antibody-epr15581-54-mitochondrial-marker-ab186735.html) | ab302554 (Abcam, Cambridge, USA) |
| Anti-P-p-65 antibody | 82335-1-RR (Proteintech, Wuhan, China ) |

**Supplementary Figure Legends**

**
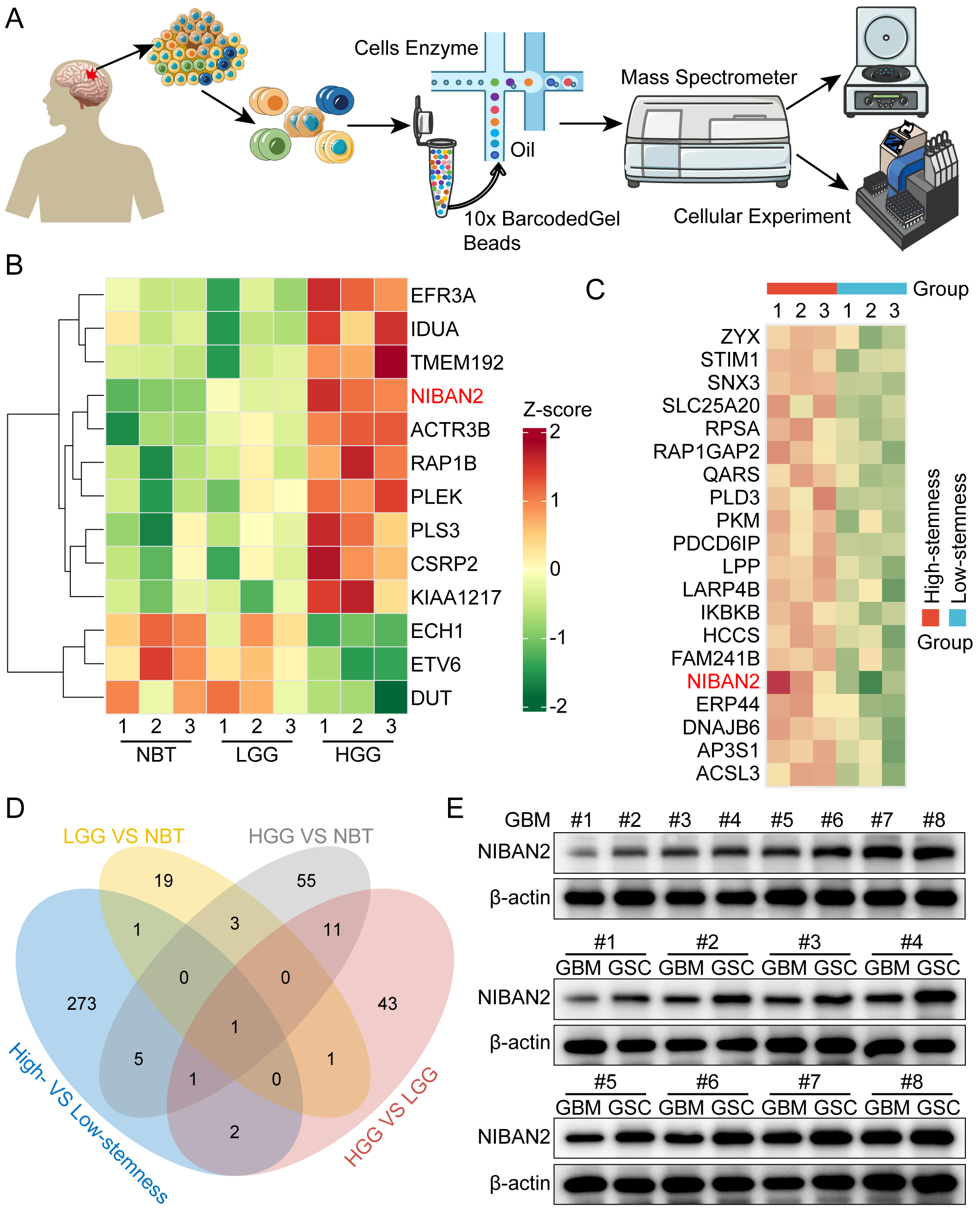
**

**Supplementary Fig. 1 Related to Fig. 1**

**(A-C)** Protein array profiling of human clinical samples across normal brain tissue (NBT), low-grade glioma (LGG), and high-grade glioma (HGG) tissues. Representative heatmaps and quantitative comparisons reveal elevated NIBAN2 protein levels in glioma samples. **(D)** Venn diagram showing the intersection of differentially expressed proteins across comparisons between normal, LGG, and HGG tissues, highlighting NIBAN2 as a candidate regulator. **(E)** Western blot analysis of NIBAN2 expression in eight primary GBM cell lines and their corresponding glioma stem-like cell (GSC) counterparts. GSCs exhibit markedly increased NIBAN2 levels compared to their parental tumor cells, particularly in cell lines #5-#8.

**
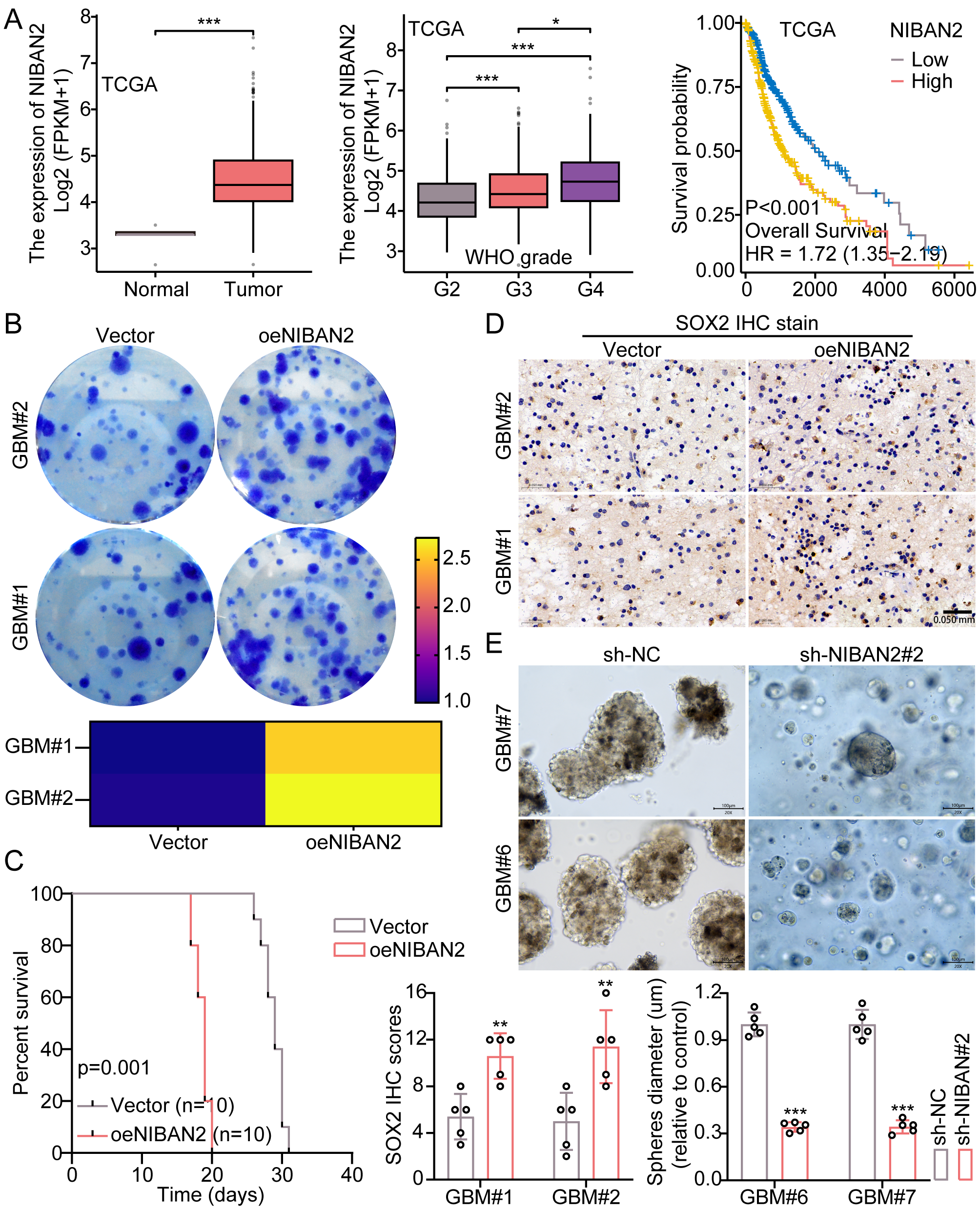
**

**Supplementary Fig. 2 Related to Fig. 2**

**(A)** Transcriptomic analysis of TCGA glioma cohorts showing significantly elevated NIBAN2 expression in glioma tissues compared to normal brain tissues. High NIBAN2 expression is associated with poor overall survival (log-rank test). **(B)** Soft agar colony formation assay showing that NIBAN2 overexpression promotes anchorage-independent growth of GSCs. Quantification reveals an approximately two-fold increase in both colony number and average diameter (P < 0.01; n = 3 biologically independent replicates; two-tailed unpaired Student’s t-test). **(C)** Kaplan-Meier survival analysis of immunodeficient mice intracranially injected with control or NIBAN2-overexpressing glioma stem-like cells (GSCs). NIBAN2-overexpressing GSCs significantly shorten overall survival compared to controls (n = 10 mice per group; log-rank test, P = 0.001). **(D)** Immunofluorescence staining for SOX2 in corresponding tumor sections from xenograft models confirms the upregulation of stemness and proliferation markers in the NIBAN2-overexpression group. DAPI was used for nuclear counterstaining. **(E)** Functional knockdown of NIBAN2 in GSCs results in impaired colony formation in vitro, supporting the role of NIBAN2 in maintaining GSC stemness and tumorigenic potential. Data were mean ± SD. Statistical significance was calculated by 2-tailed unpaired Student’s *t* tests for **A (left)** and **C (middle and right)**; 1-way ANOVA for **A (middle)**; Survival analysis of **A (right)** and **C (left)** was performed by the log-rank test. ***P* < 0.01, ****P* < 0.001, *****P* < 0.0001.


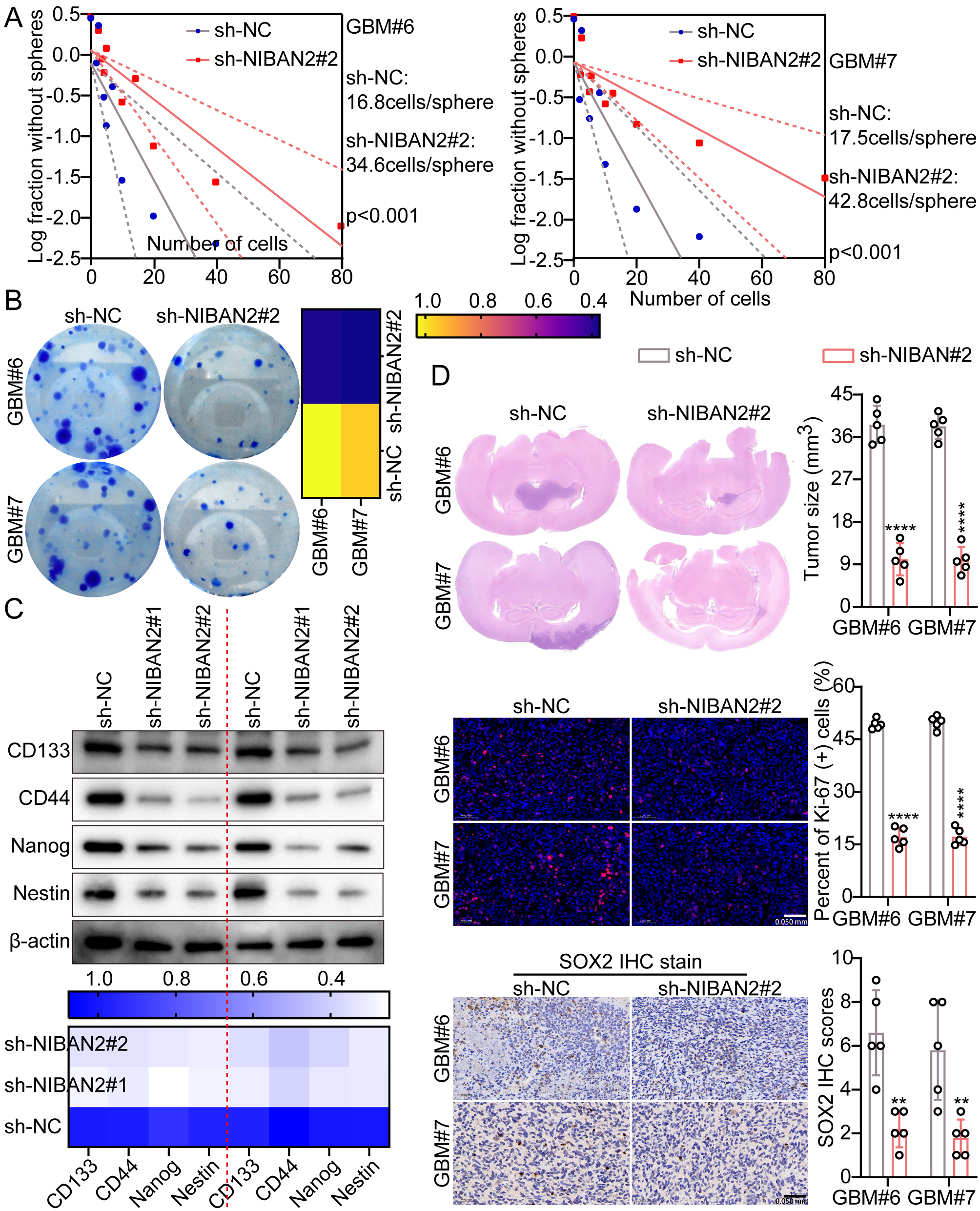


**Supplementary Fig. 3 Related to Fig. 2**

**(A)** Limiting dilution assay (LDA) assessing self-renewal capacity of GSCs with or without NIBAN2 knockdown. The frequency of stem-like cells was calculated using the ELDA algorithm. (n = 5 biologically independent experiments; mean ± s.d.; two-tailed unpaired Student’s t-test). **(B)** Soft agar colony formation assay demonstrating that silencing NIBAN2 significantly decreases both the number and diameter of colonies under anchorage-independent conditions (P < 0.01). **(C)** Western blot analysis of stemness-associated transcription factors (CD133, NANOG, CD44 and Nestin) in control and NIBAN2-knockdown GSCs. NIBAN2 silencing leads to downregulation of all four markers. β-actin was used as a loading control. **(D)** After intracranial implantation of control or NIBAN2-knockdown glioma stem-like cells (GSCs), the NIBAN2-deficient group exhibited a significantly reduced tumor volume; moreover, tumor volume was decreased and the expression levels of SOX2 and Ki-67 were lower in this group. (n = 5 per group; log-rank test, P < 0.01). Data were mean ± SD. Statistical significance was calculated by 2-tailed unpaired Student’s *t* tests for **A-C**. ***P* < 0.01, *****P* < 0.0001.

**
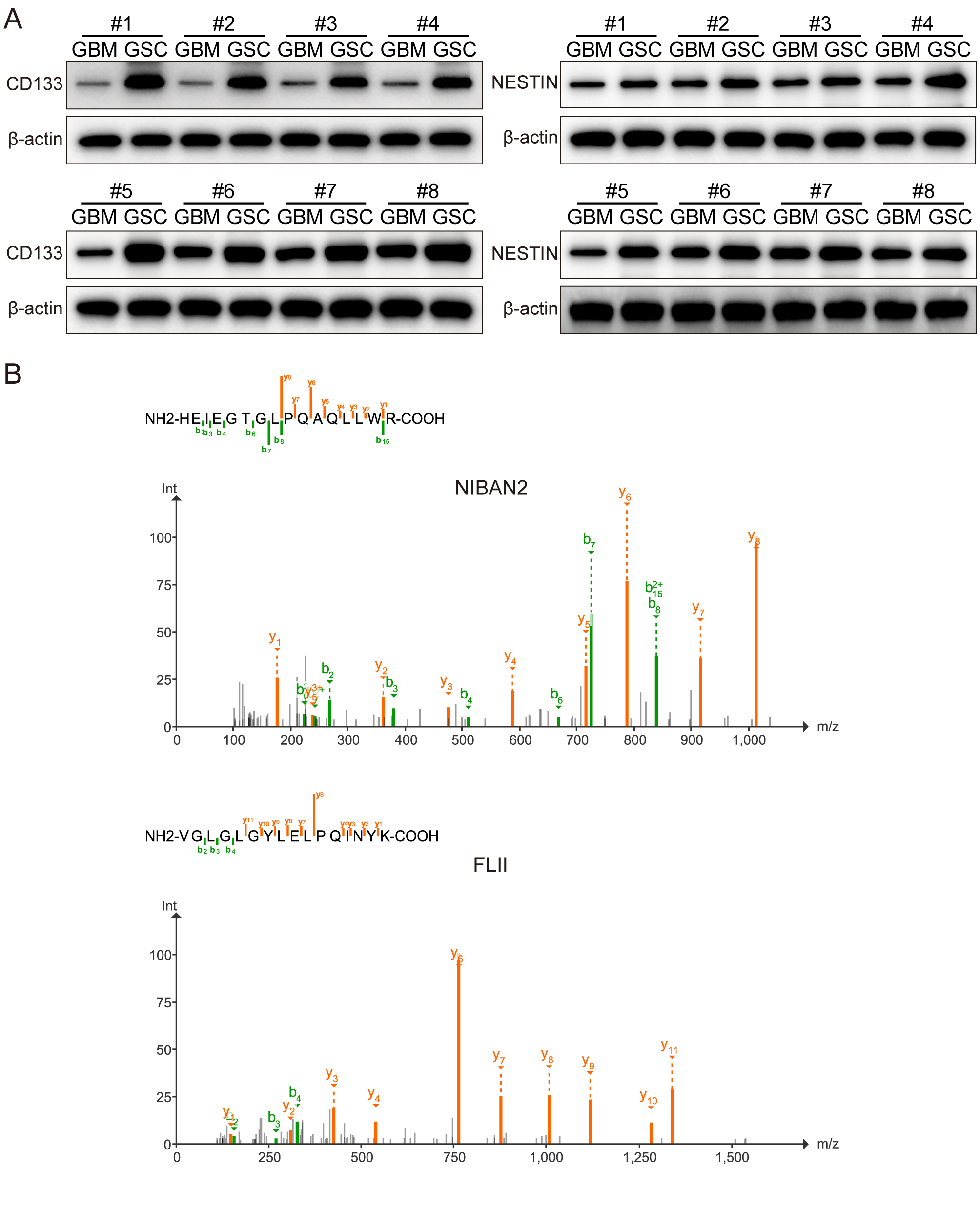
**

**Supplementary Fig. 4 Related to Fig. 3**

**(A)** Western blot analysis of CD133 and NESTIN expression in eight primary GBM cell lines and their corresponding glioma stem-like cell (GSC) counterparts. **(B)** MS/MS spectrum.

**
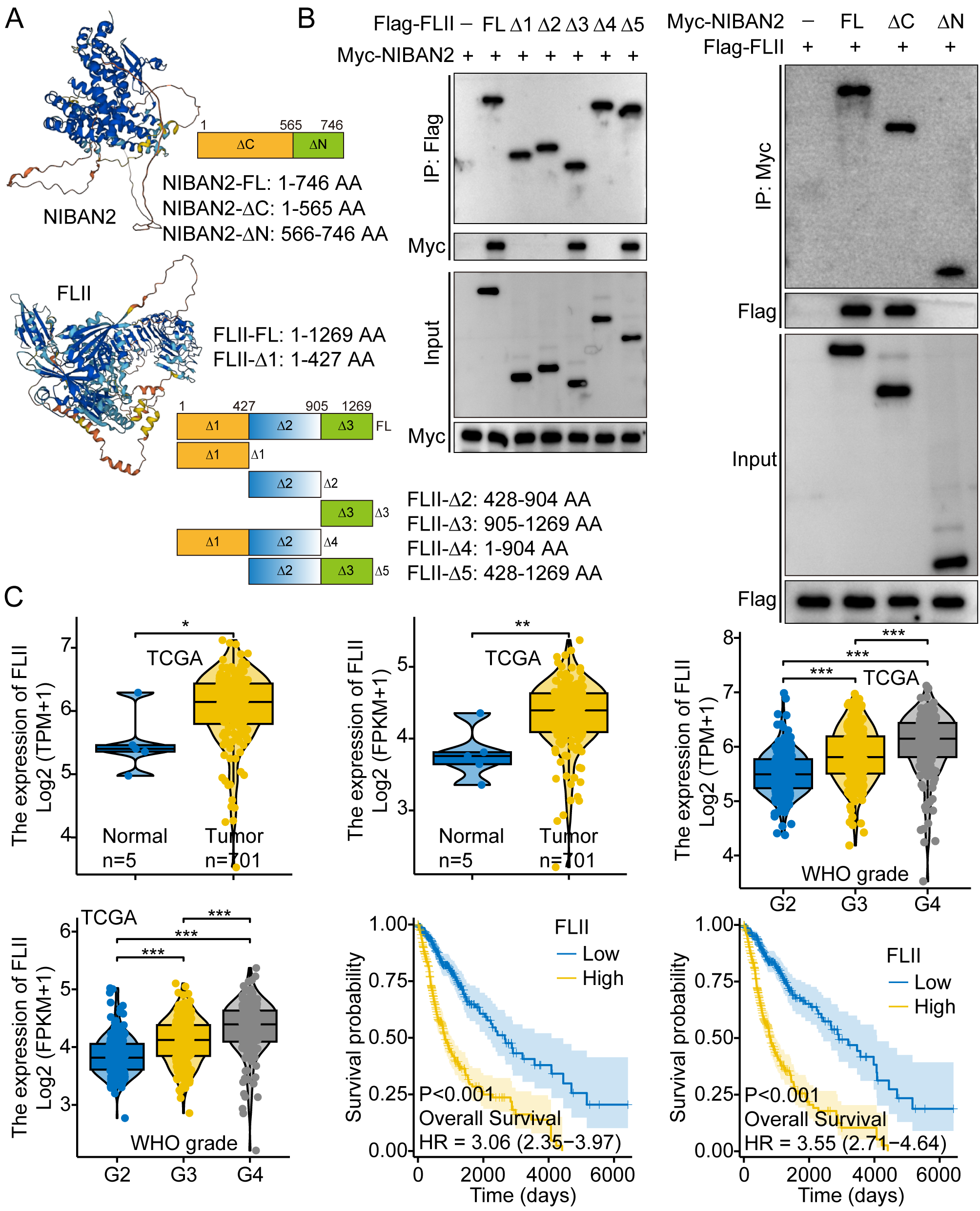
**

**Supplementary Fig. 5 Related to Fig. 3**

**(A-B)** Schematic representation of the domain architecture of full-length and truncated NIBAN2 constructs (Full, ΔN, ΔC) used to map the FLII-binding region. Co-immunoprecipitation assays revealed that deletion of the C-terminal region (aa566-746) abolished FLII binding, indicating its necessity for interaction. Domain mapping of FLII using a series of truncated mutants (Δ1-Δ5) co-expressed with NIBAN2. Co-immunoprecipitation showed that the region spanning aa428-904 of FLII is essential for binding to NIBAN2. **(C)** Kaplan-Meier survival analysis of glioma patients stratified by FLII expression in The Cancer Genome Atlas (TCGA) cohort. High FLII expression was significantly associated with poorer overall survival. Statistical significance was determined using the log-rank test. Data were mean ± SD. Statistical significance was calculated by 2-tailed unpaired Student’s *t* tests, 1-way ANOVA and the log-rank test for **C**. **P* < 0.05, ***P* < 0.01, ****P* < 0.001.

**
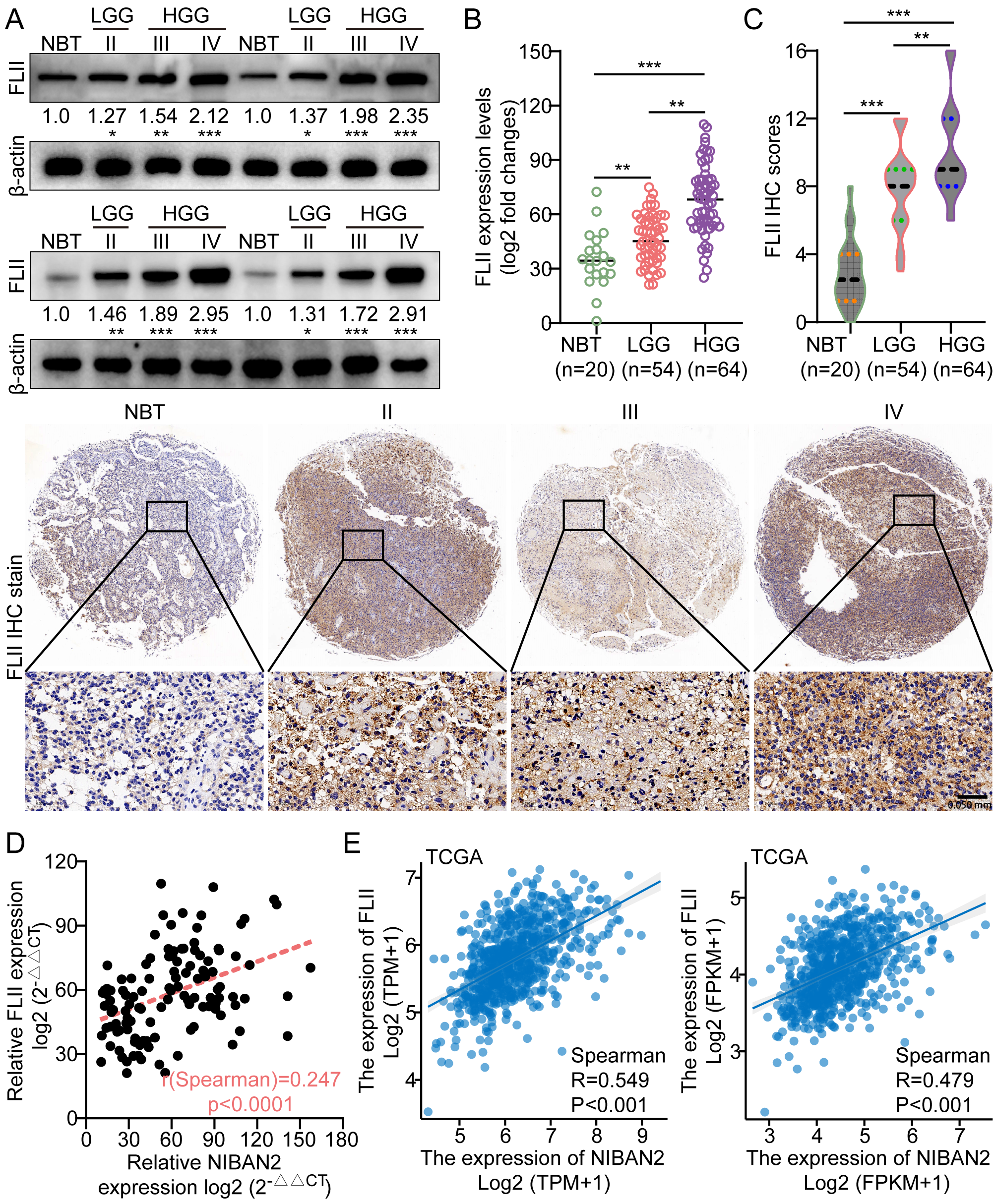
**

**Supplementary Fig. 6 Related to Fig. 3**

**(A)** Western blot analysis of FLII protein levels in normal brain tissue (NBT), low-grade glioma (LGG), and high-grade glioma (HGG) clinical samples. FLII expression progressively increased with tumor grade. β-actin served as the loading control. **(B)** Quantitative PCR (qPCR) analysis of FLII mRNA expression in NBT, LGG, and HGG tissues. FLII transcript levels were significantly elevated in glioma compared to NBT. **(C)** Representative immunohistochemistry (IHC) images showing FLII expression in NBT, LGG, and HGG tissues. Scale bars, 0.05 mm. **(D)** Correlation analysis between NIBAN2 and FLII mRNA levels in clinical glioma samples, as determined by densitometric quantification of PCR. Pearson’s correlation coefficient (r) is shown. **(E)** Correlation between NIBAN2 and FLII mRNA expression in glioma samples from the TCGA dataset. A strong positive correlation was observed. Statistical significance was assessed by Pearson’s correlation test. Data were mean ± SD. Statistical significance was calculated by 1-way ANOVA for **A**, **B** and **C**; Spearman’s rank correlation test for **D** and **E**. **P* < 0.05, ***P* < 0.01, ****P* < 0.001, *****P* < 0.0001.

**
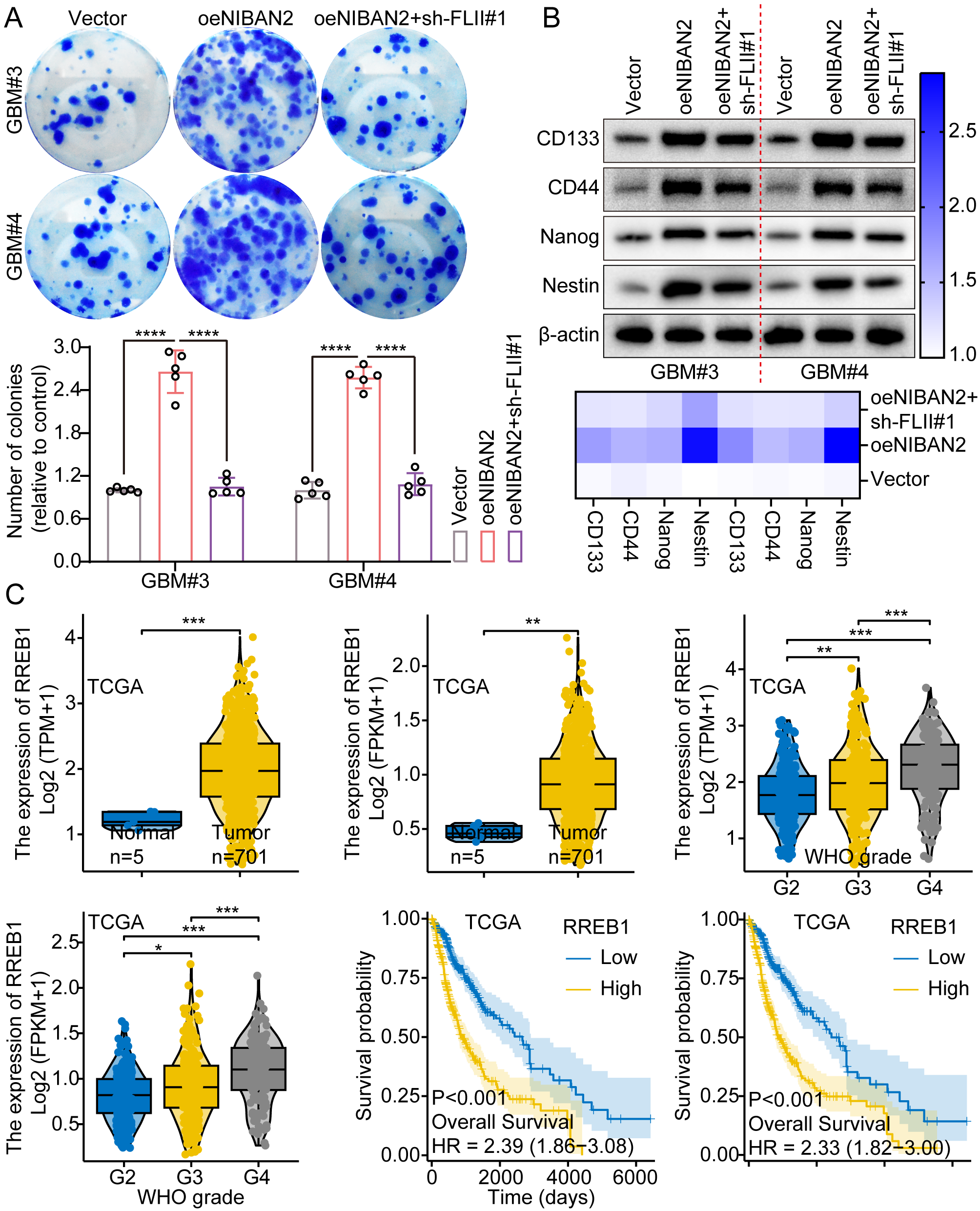
**

**Supplementary Fig. 7 Related to Fig. 4**

**(A)** Representative images and quantification of soft agar colony formation assays in GSCs with control, NIBAN2 overexpression (NIBAN2-OE), or NIBAN2-OE combined with FLII knockdown (NIBAN2-OE + shFLII). NIBAN2 overexpression significantly increased both colony number and diameter, while FLII knockdown attenuated this effect (~60% reduction in colony number, P < 0.01). **(B)** Western blot analysis of stemness markers (CD44, CD133, NANOG) in GSCs under the indicated conditions. FLII knockdown reversed the upregulation of stemness-associated proteins induced by NIBAN2 overexpression. β-actin served as the loading control. **(C)** Kaplan-Meier survival analysis of glioma patients stratified by RREB1 expression in The Cancer Genome Atlas (TCGA) cohort. High RREB1 expression was significantly associated with poorer overall survival. Statistical significance was determined using the log-rank test. Data were mean ± SD. Statistical significance was calculated by 1-way ANOVA for **A**; 2-tailed unpaired Student’s *t* tests, 1-way ANOVA and the log-rank test for **C**. **P* < 0.05, ***P* < 0.01, ****P* < 0.001, *****P* < 0.0001.

**
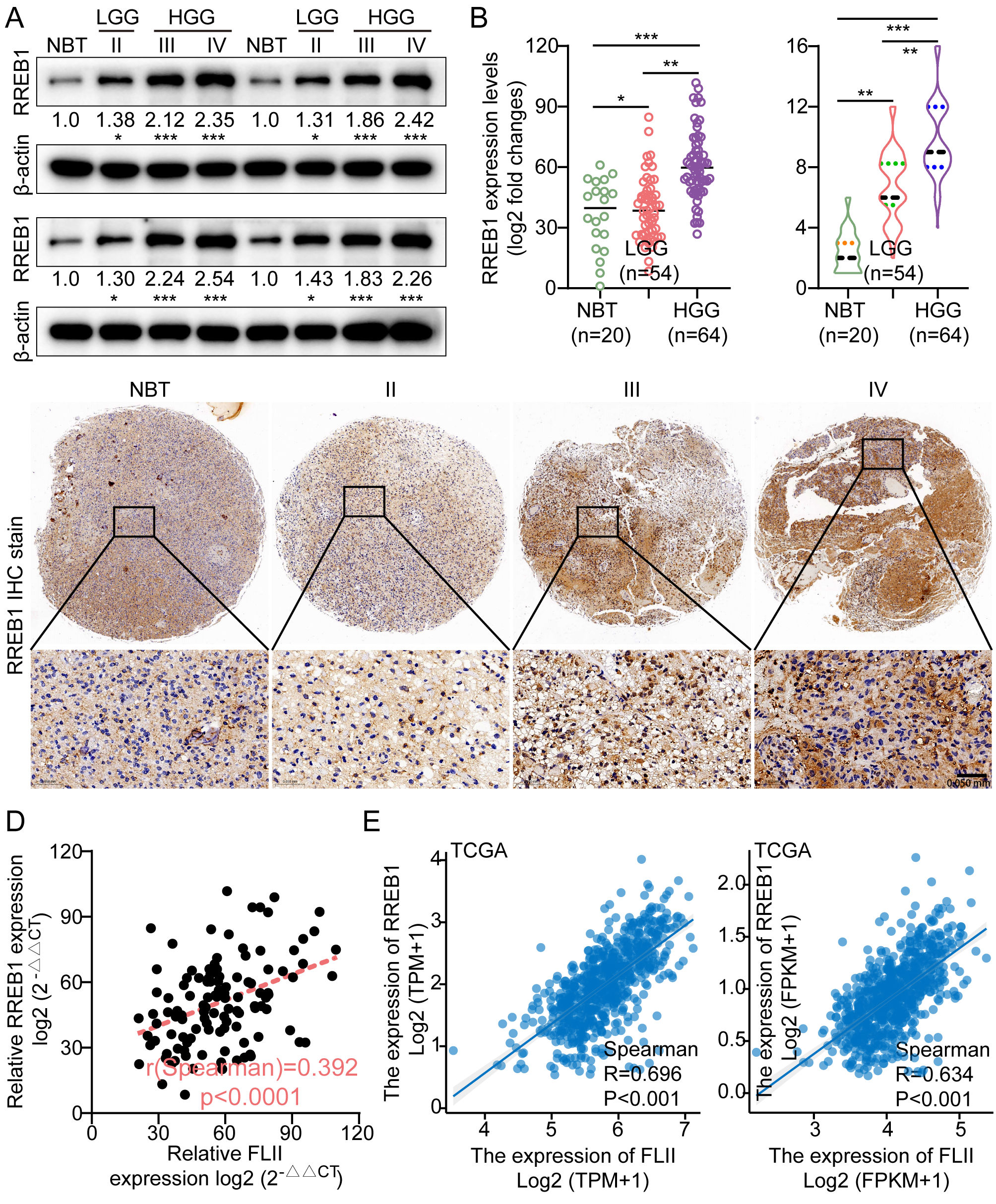
**

**Supplementary Fig. 8 Related to Fig. 5**

**(A)** Western blot analysis of RREB1 protein levels in normal brain tissue (NBT), low-grade glioma (LGG), and high-grade glioma (HGG) clinical samples. FLII expression progressively increased with tumor grade. β-actin served as the loading control. **(B)** Quantitative PCR (qPCR) analysis of RREB1 mRNA expression in NBT, LGG, and HGG tissues. FLII transcript levels were significantly elevated in glioma compared to NBT. **(C)** Representative immunohistochemistry (IHC) images showing RREB1 expression in NBT, LGG, and HGG tissues. Scale bars, 0.05 mm. **(D)** Correlation analysis between RREB1 and FLII mRNA levels in clinical glioma samples, as determined by densitometric quantification of PCR. Pearson’s correlation coefficient (r) is shown. **(E)** Correlation between RREB1 and FLII mRNA expression in glioma samples from the TCGA dataset. A strong positive correlation was observed. Statistical significance was assessed by Pearson’s correlation test. Data were mean ± SD. Statistical significance was calculated by 1-way ANOVA for **A**, **B** and **C**; Spearman’s rank correlation test for **D** and **E**. **P* < 0.05, ***P* < 0.01, ****P* < 0.001, *****P* < 0.0001.


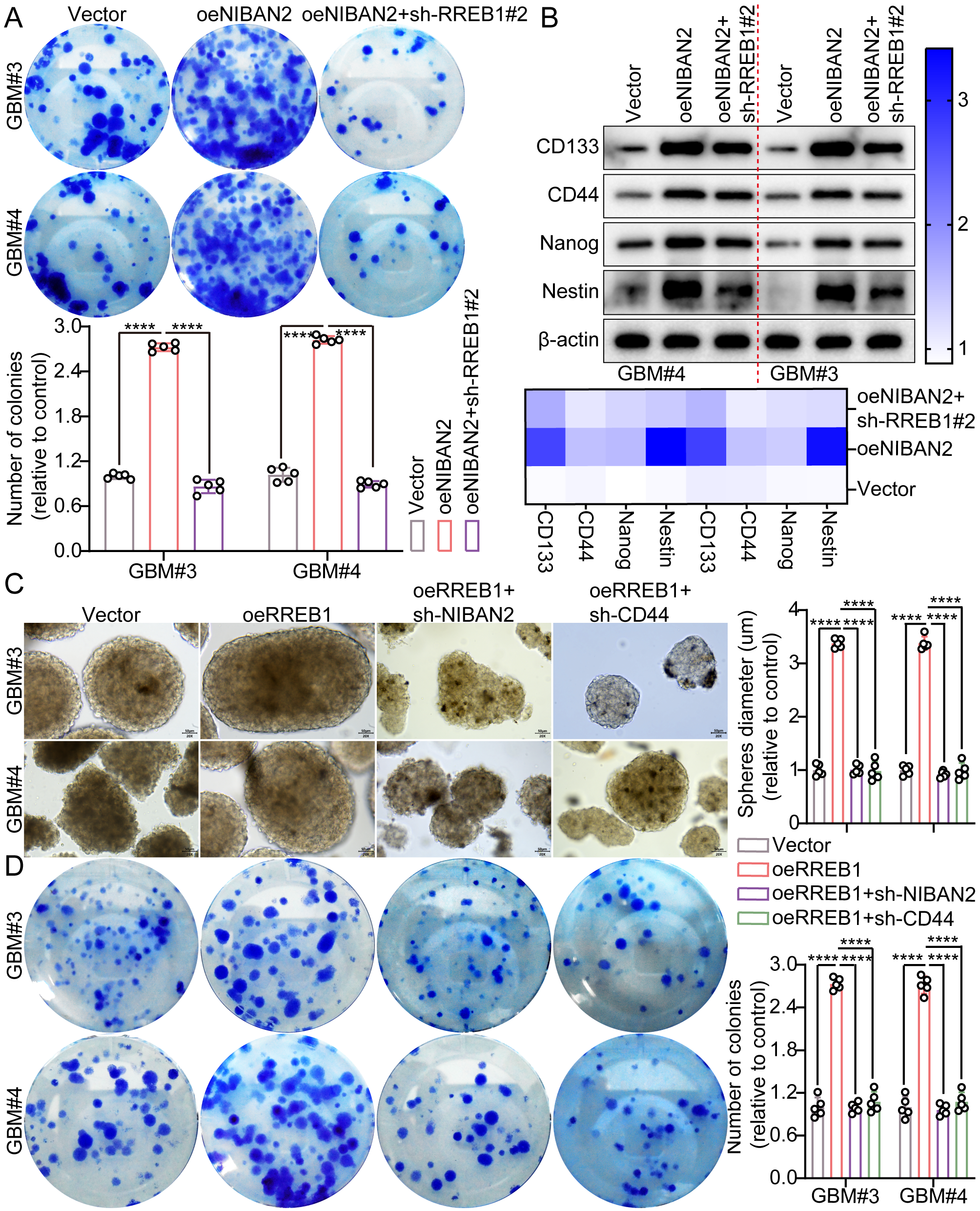


**Supplementary Fig. 9 Related to Fig. 6-7**

**(A)** Soft agar colony formation assay showing that RREB1 knockdown markedly impairs the anchorage-independent growth promoted by NIBAN2 overexpression in glioma stem-like cells (GSCs). Representative images and quantification of colony number and diameter are shown. Data represent mean ± s.d. from three independent experiments. P < 0.01, one-way ANOVA with Tukey’s post hoc test. **(B)** Western blot analysis demonstrating that RREB1 knockdown reverses the NIBAN2-induced upregulation of stemness-associated markers CD44, CD133, and NANOG in GSCs. β-actin serves as a loading control. Data are representative of at least three independent experiments. **(C)** Neurosphere formation assay showing that RREB1 overexpression significantly increases sphere-forming capacity in glioma stem-like cells (GSCs), while knockdown of NIBAN2 or CD44 partially abrogates these effects. Quantification of sphere number and representative immunoblot analysis are shown. Data represent mean ± s.d. from three independent experiments. P < 0.01, one-way ANOVA with Tukey’s post hoc test. **(D)** Soft agar colony formation assay demonstrating that the clonogenic advantage conferred by RREB1 overexpression is attenuated upon knockdown of NIBAN2 or CD44. Representative colony images and quantification of colony number and size are presented. Data are shown as mean ± s.d. (n = 5 independent experiments). P < 0.01, one-way ANOVA. Data were mean ± SD. Statistical significance was calculated by 1-way ANOVA for **A**; 2-way ANOVA for **C** and **D**. *****P* < 0.0001.


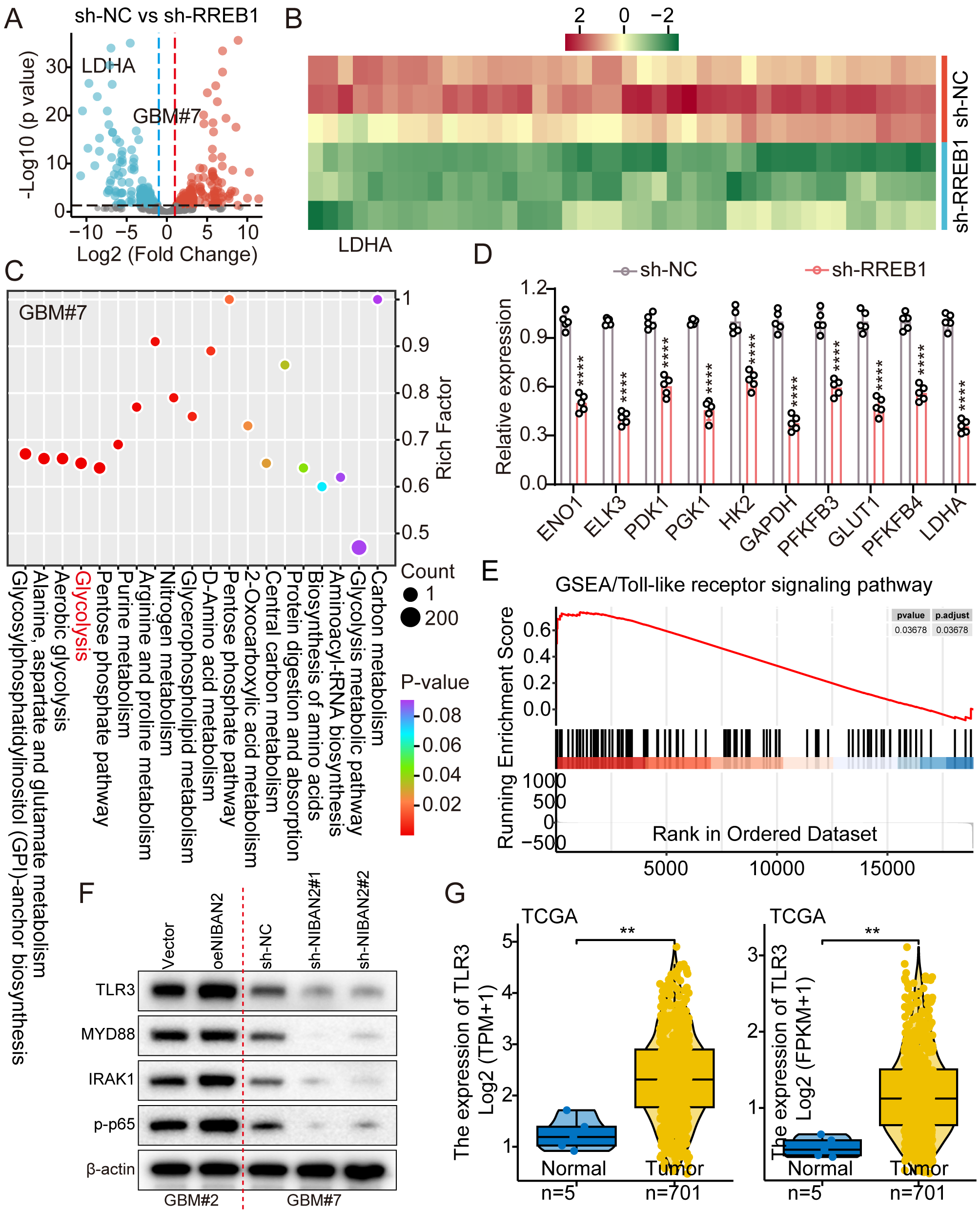


**Supplementary Fig. 10 Related to Fig. 8**

**(A)** Volcano plot illustrating differentially expressed genes upon RREB1 knockdown in GSCs, highlighting significant downregulation of glycolysis-related genes, with LDHA among the most suppressed targets (log₂FC = -1.7, adjusted P < 0.001). **(B)** Heatmap of selected glycolysis-associated genes from RNA-seq data showing marked downregulation in RREB1-deficient GSCs. Data are Z-score normalized across samples. **(C)** KEGG pathway enrichment analysis of downregulated genes upon RREB1 knockdown, with glycolysis/gluconeogenesis ranking among the top enriched pathways. **(D)** Expression levels of key glycolytic enzymes (e.g., HK2, PFKP, PGK1, ENO1) are significantly reduced at the mRNA level in RREB1-deficient cells, as determined by RNA-seq. **(E)** Gene Set Enrichment Analysis (GSEA) showing significant downregulation of the glycolysis hallmark gene set in RREB1-deficient GSCs (NES = -1.92, FDR < 0.001). **(F)** Western blot validation of Toll-like receptor (TLR) pathway activation by NIBAN2. Overexpression of NIBAN2 increases protein levels of TLR4, MYD88, TRAF6, and phospho-p65, whereas NIBAN2 knockdown reduces their expression. **(G)** Relative expression levels of TLR3 in glioma versus NBT based on TCGA RNA-seq data. Gene expression is significantly elevated in glioma samples (P < 0.001, unpaired two-tailed t-test). Data were mean ± SD. Statistical significance was calculated by 2-tailed unpaired Student’s *t* tests for **D** and **G**. ***P* < 0.01, *****P* < 0.0001.


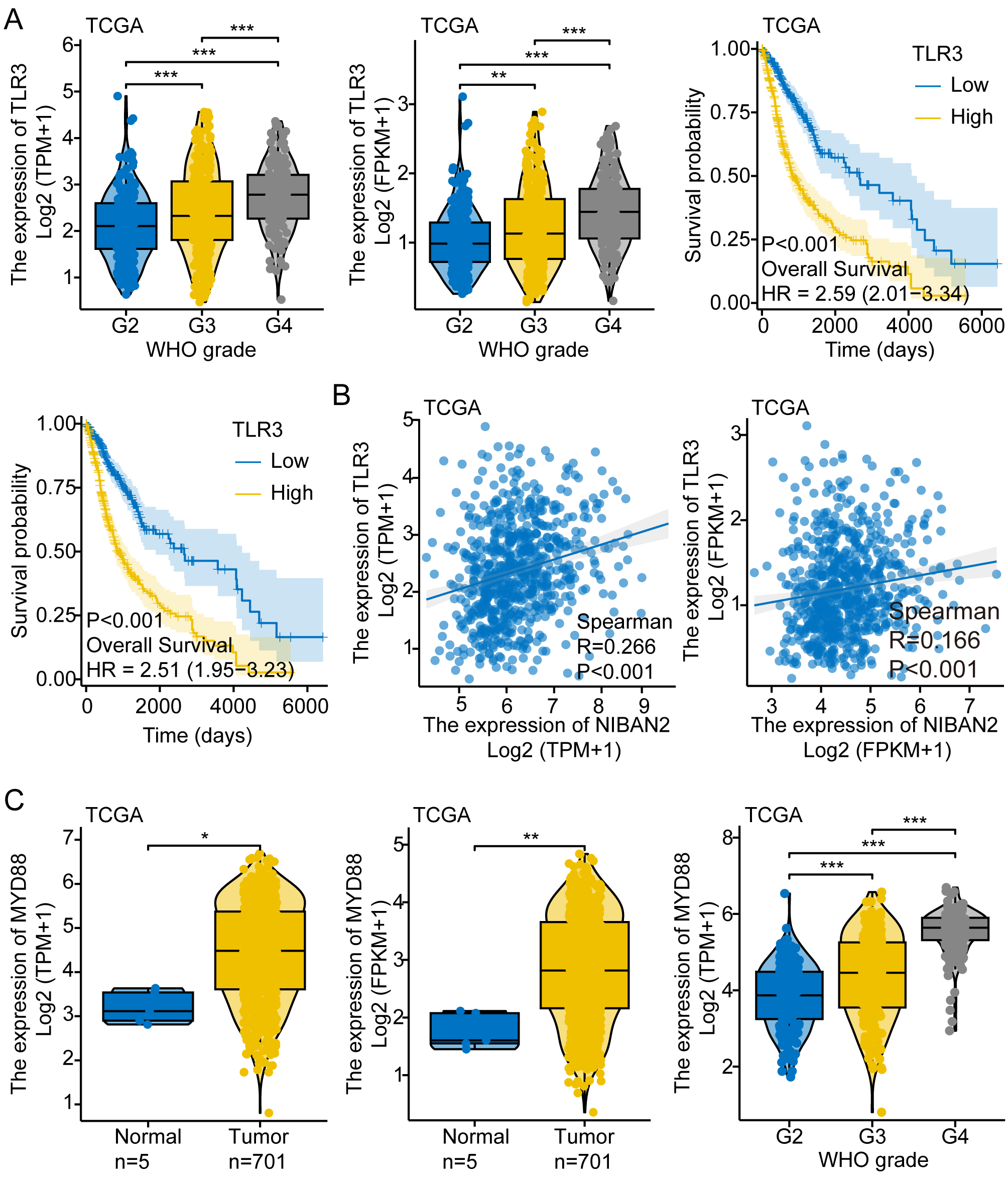


**Supplementary Fig. 11 Related to Fig. 8**

**(A)** Transcriptomic analysis of TCGA glioma cohorts showing significantly elevated TLR3 expression in glioma tissues compared to normal brain tissues. High TLR3 expression is associated with poor overall survival (log-rank test). **(B)** Correlation between NIBAN2 and TLR3 mRNA expression in glioma samples from the TCGA dataset. A strong positive correlation was observed. Statistical significance was assessed by Pearson’s correlation test. **(C)** Relative expression levels of MYD88 in glioma versus NBT based on TCGA RNA-seq data. Gene expression is significantly elevated in glioma samples (P < 0.001, unpaired two-tailed t-test). Data were mean ± SD. Statistical significance was calculated by 1-way ANOVA and the log-rank test for **A**; Spearman’s rank correlation test for **B**; 2-tailed unpaired Student’s *t* tests and 1-way ANOVA for **C**. **P* < 0.05, ***P* < 0.01, ****P* < 0.001.


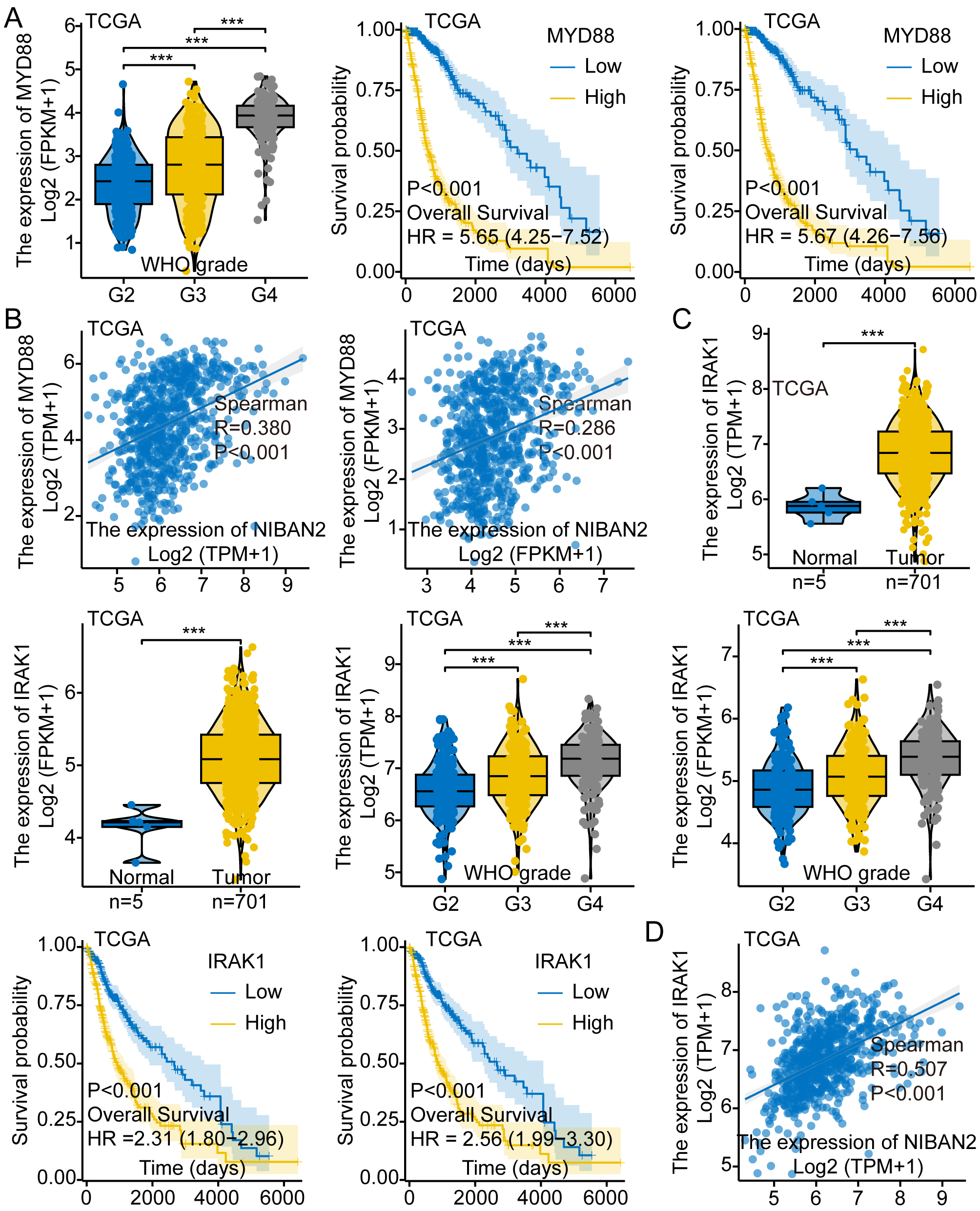


**Supplementary Fig. 12 Related to Fig. 8**

**(A)** Transcriptomic analysis of TCGA glioma cohorts showing significantly elevated MYD88 expression in glioma tissues compared to normal brain tissues. High MYD88 expression is associated with poor overall survival (log-rank test). **(B)** Correlation between NIBAN2 and MYD88 mRNA expression in glioma samples from the TCGA dataset. A strong positive correlation was observed. Statistical significance was assessed by Pearson’s correlation test. **(C)** Transcriptomic analysis of TCGA glioma cohorts showing significantly elevated IRAK1 expression in glioma tissues compared to normal brain tissues. High IRAK1 expression is associated with poor overall survival (log-rank test). **(D)** Correlation between NIBAN2 and IRAK1 mRNA expression in glioma samples from the TCGA dataset. A strong positive correlation was observed. Statistical significance was assessed by Pearson’s correlation test. Data were mean ± SD. Statistical significance was calculated by 1-way ANOVA and the log-rank test for **A**; Spearman’s rank correlation test for **B** and **D**; 2-tailed unpaired Student’s *t* tests and 1-way ANOVA for **C**. ****P* < 0.001.


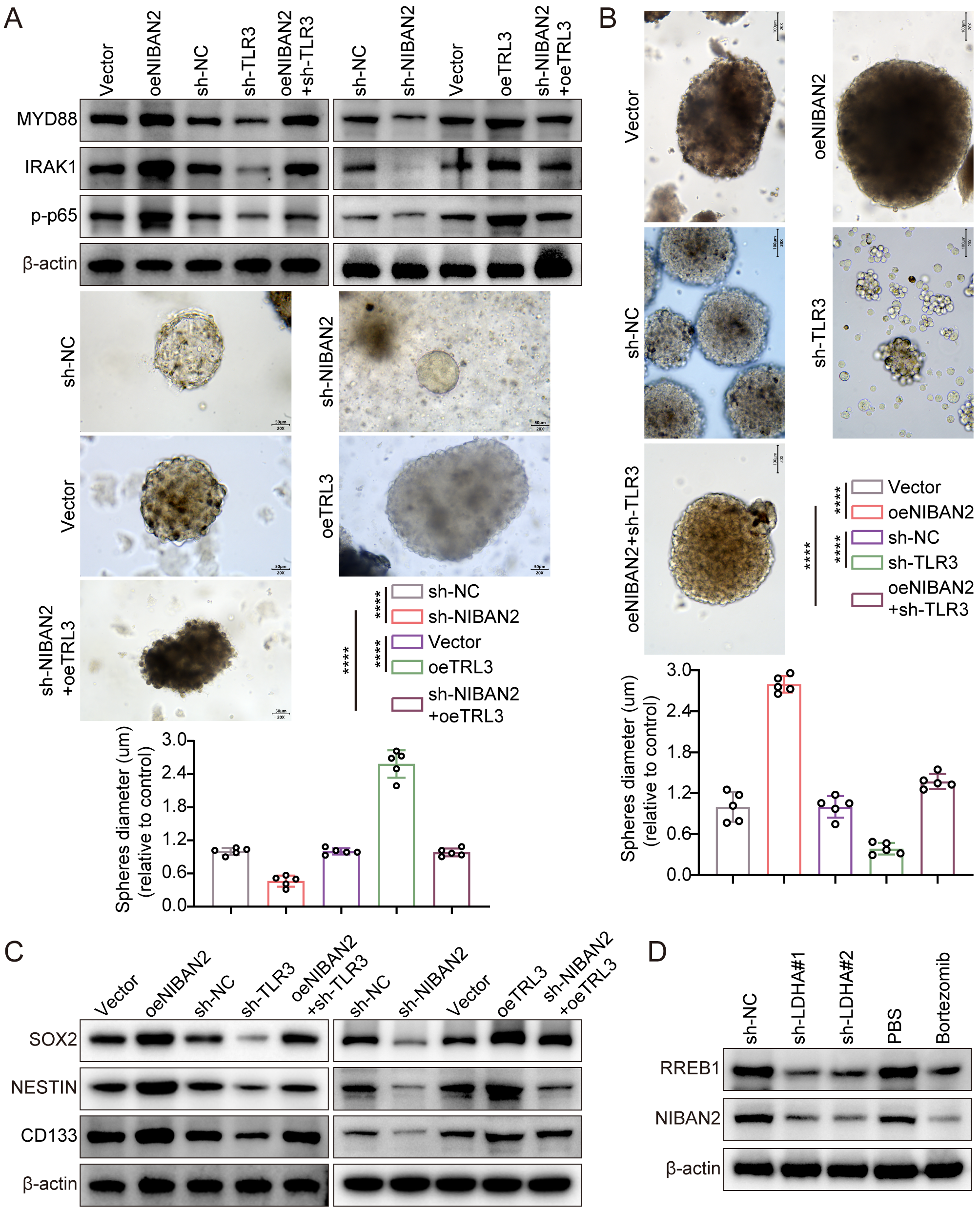


**Supplementary Fig. 13 Related to Fig. 8**

**(A)** WB analysis of MYD88/IRAK1/P-p65 in the different experimental groups. β-actin served as the loading control. **(B)** Representative images and quantification of tumor sphere formation in the different experimental groups GSCs cultured under serum-free, low-adhesion conditions. **(C)** WB analysis of SOX2/NESTIN/CD133 in the different experimental groups. β-actin served as the loading control. **(D)** WB analysis of RREB1/NIBAN2 in the different experimental groups. β-actin served as the loading control.

Data were mean ± SD. Statistical significance was calculated by 2-way ANOVA for **A** and **B**. *****P* < 0.0001.


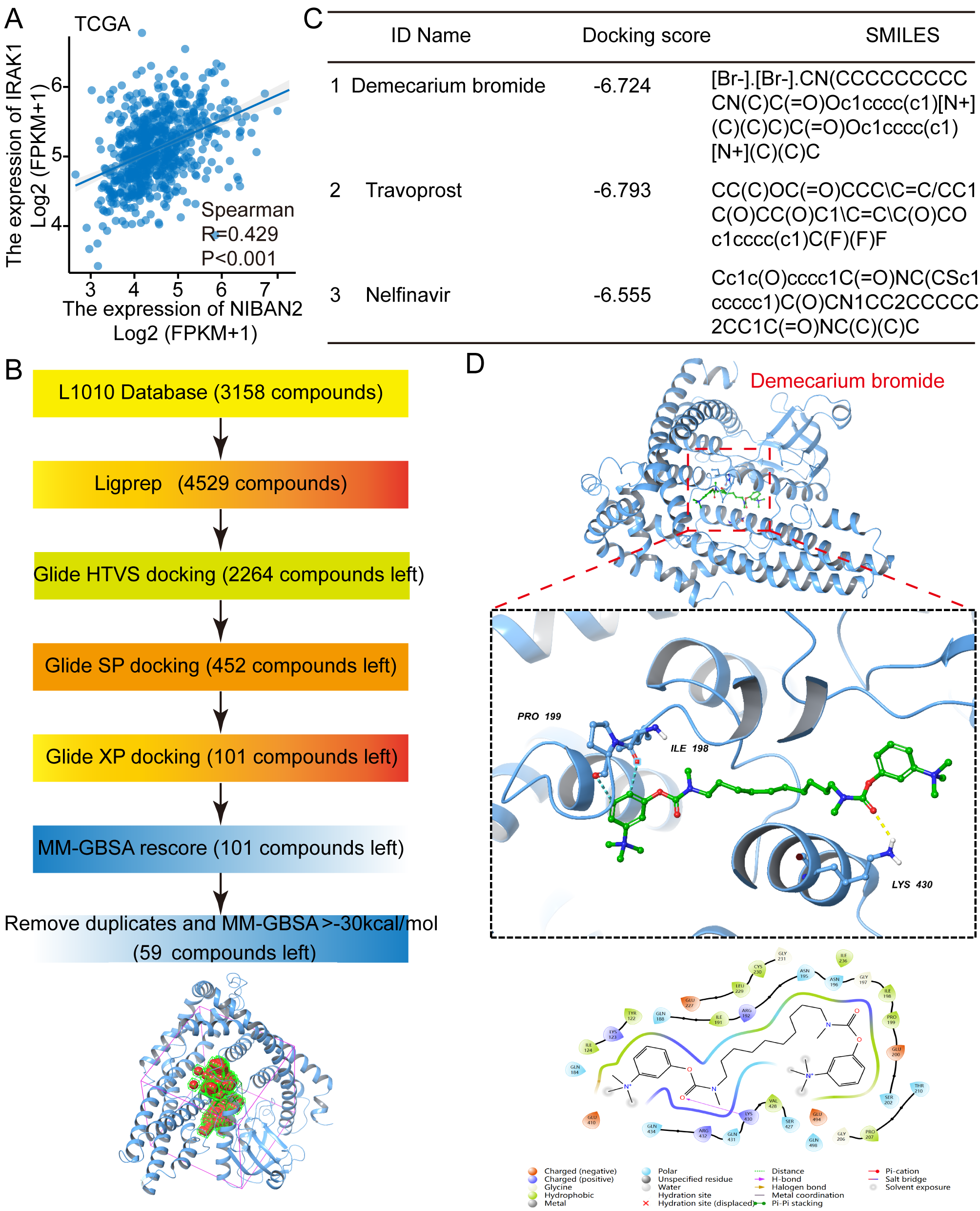


**Supplementary Fig. 14 Related to Fig. 9**

**(A)** Correlation between NIBAN2 and IRAK1 mRNA expression in glioma samples from the TCGA dataset. A strong positive correlation was observed. Statistical significance was assessed by Pearson’s correlation test. **(B)** Schematic diagram illustrating the stepwise virtual screening pipeline based on the NIBAN2 crystal structure (PDB: 7CTP), including protein preparation, binding site prediction (SiteMap), grid generation, high-throughput virtual screening (HTVS), standard precision (SP), extra precision (XP) docking, and MM-GBSA rescoring. The number of compounds retained at each stage is indicated. **(C)** Basic information of the top three candidate compounds targeting NIBAN2. **(D)** Schematic representation of the chemical structure of Demecarium bromide.


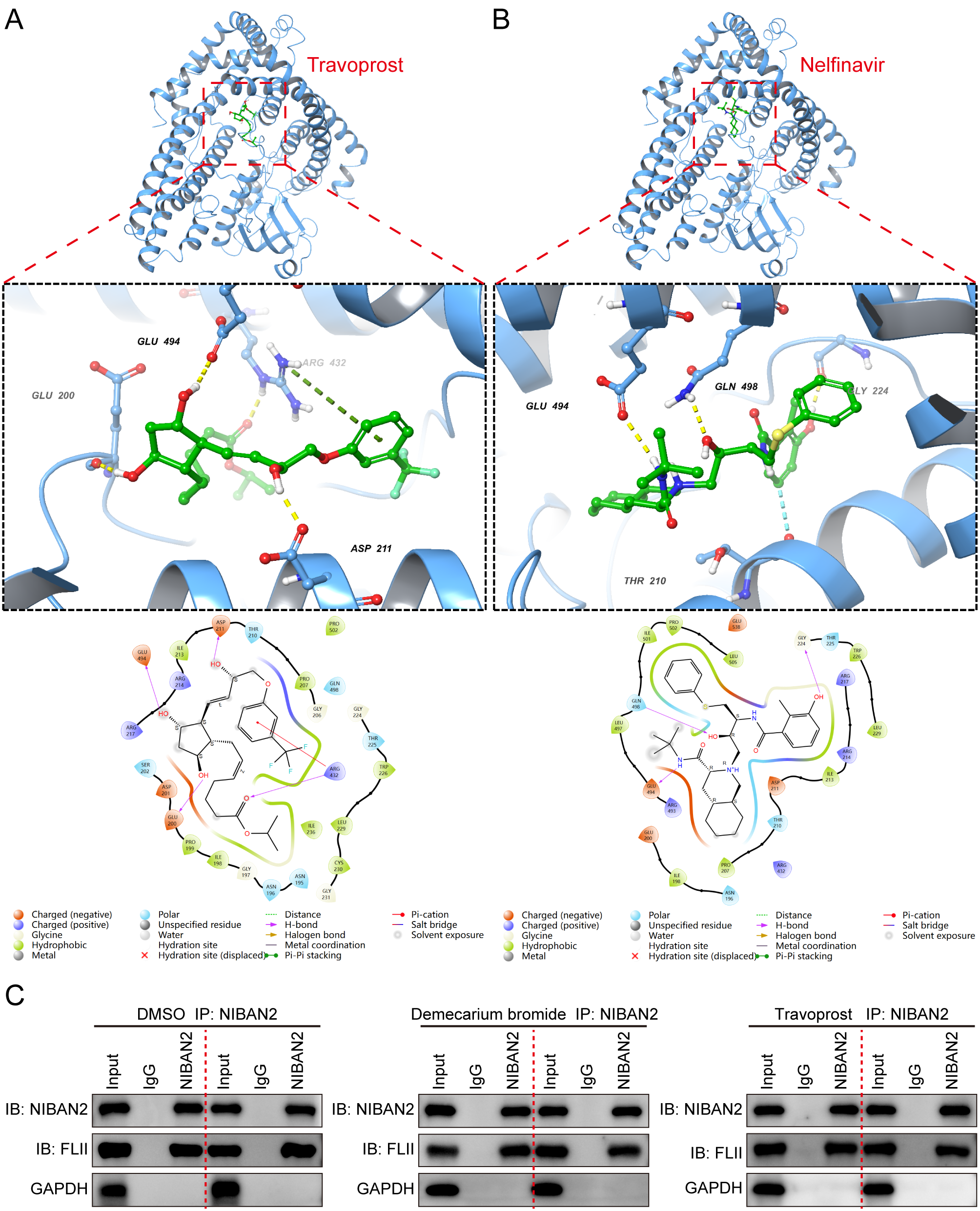


**Supplementary Fig. 15 Related to Fig. 9**

**(A-B)** Schematic representation of the chemical structure of Travoprost and Nelfinavir. **(C)** Co-immunoprecipitation of NIBAN2-FLII in GSCs treated with DMSO, Demecarium bromide, Travoprost, or Nelfinavir. Only Nelfinavir markedly reduced FLII binding.


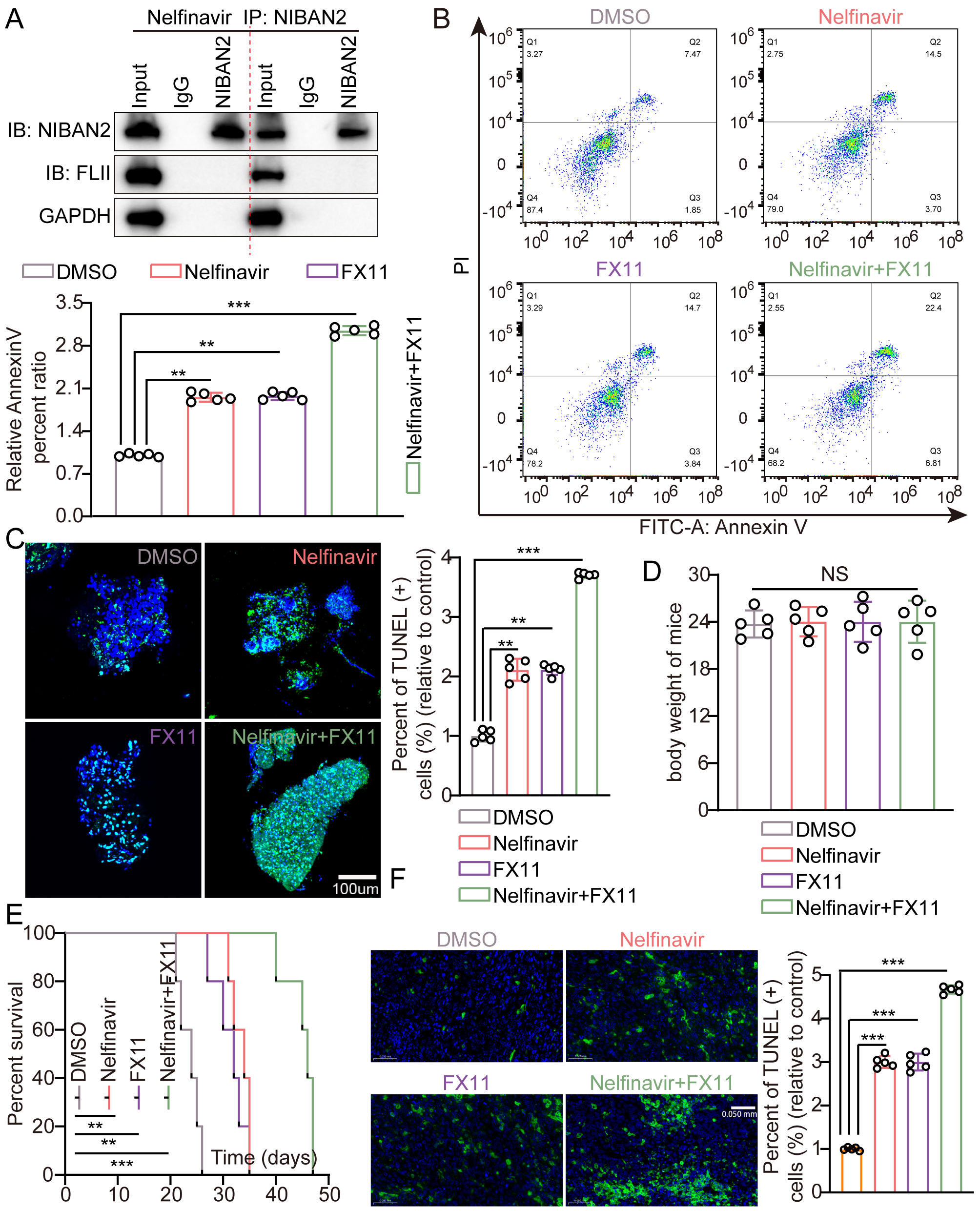


**Supplementary Fig. 16 Related to Fig. 9**

**(A)** Co-immunoprecipitation showing that only Nelfinavir disrupts NIBAN2-FLII binding among tested compounds. **(B)** Caspase-3/7 activity and Annexin V staining in PDOs treated with Nelfinavir, FX11, or their combination. **(C)** Immunofluorescence of PDOs showing increased TUNEL positivity upon combined treatment. **(D)** Body weight monitoring of PDX mice treated with vehicle, Nelfinavir, FX11, or the combination. **(E)** Kaplan-Meier survival curves demonstrating prolonged survival in the combination group. **(F)** IF analysis of PDX tumors showing increased TUNEL-positive cells after combined treatment. Data were mean ± SD. Statistical significance was calculated by 2-way ANOVA for **B**, **C**, **D** and **F**; Survival analysis of **E** was performed by the log-rank test. ***P* < 0.01, ****P* < 0.001.


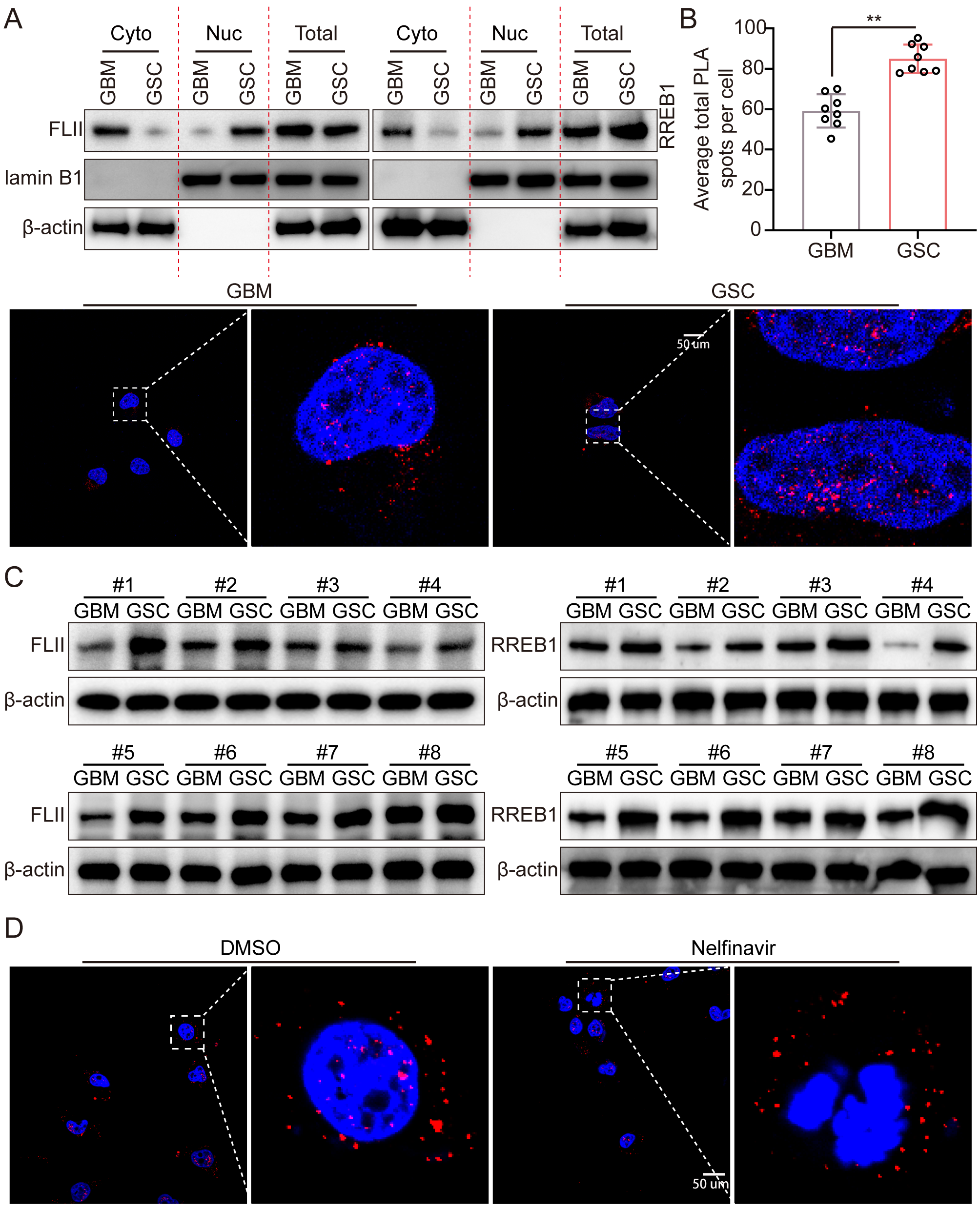


**Supplementary Fig. 17 Related to Fig. 9**

**(A)** Nuclear and cytoplasmic fractionation assays show that, compared with the parental GBM cells, GSCs exhibit enhanced nuclear localization of FLII and RREB1. Lamin B1 and β-actin served as markers for the nucleus and cytoplasm, respectively. **(B)** Representative PLA images of FLII and RREB1 in parental GBM cells and their corresponding GSCs. **(C)** Western blot analysis of FLII and RREB1 expression in eight primary GBM cell lines and their corresponding glioma stem-like cell (GSC) counterparts. **(D)** Representative PLA images of FLII and RREB1 under the different experimental groups. Data were mean ± SD. Statistical significance was calculated by 2-tailed unpaired Student’s *t* tests for **B**. ***P* < 0.01.


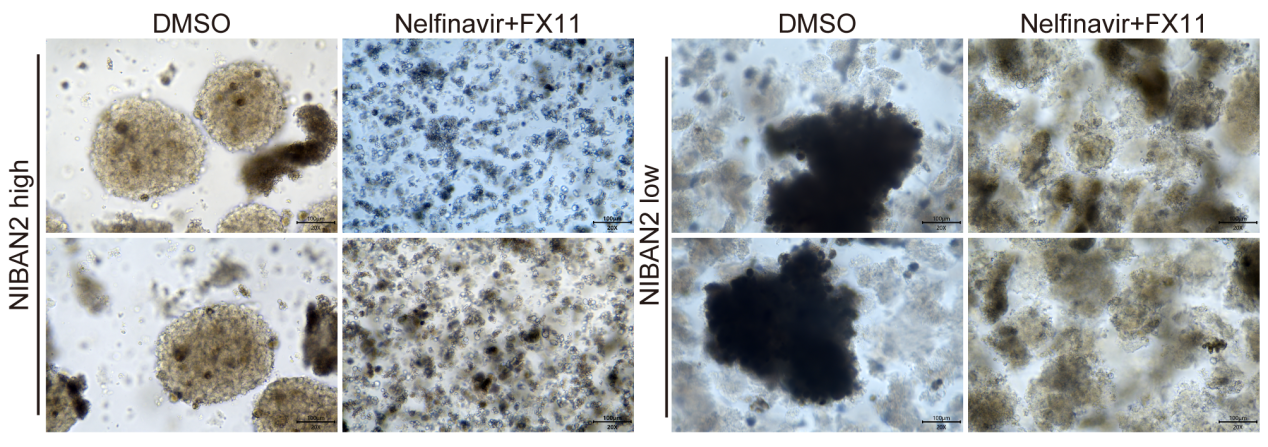


**Supplementary Fig. 18 Related to Fig. 9**

Representative images showing the responses of NIBAN2-high and NIBAN2-low groups to the different treatment conditions.


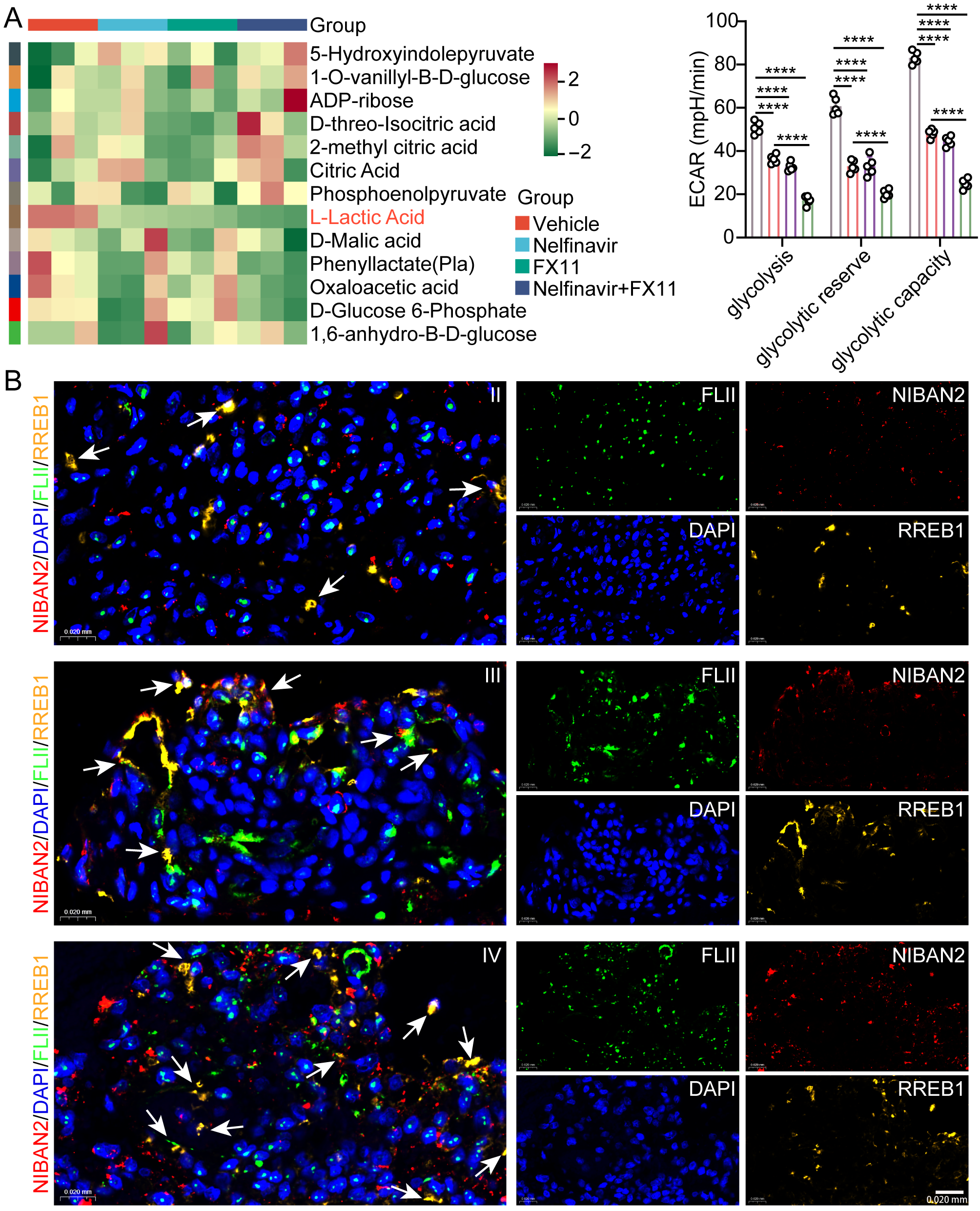


**Supplementary Fig. 19 Related to Fig. 10**

**(A)** Targeted metabolomics confirms glycolytic suppression upon combined treatment. Intracellular lactate levels and ECAR measurements showing reduced glycolytic flux in treated PDX tumors. **(B)** Positive correlation of NIBAN2/FLII/RREB1 co-expression with WHO grade. Proportion of tumors with high NIBAN2, FLII, and RREB1 expression across different WHO grades, showing increased frequency in GBM compared to LGG. Data were mean ± SD. Statistical significance was calculated by 2-way ANOVA for **A**. *****P* < 0.0001.


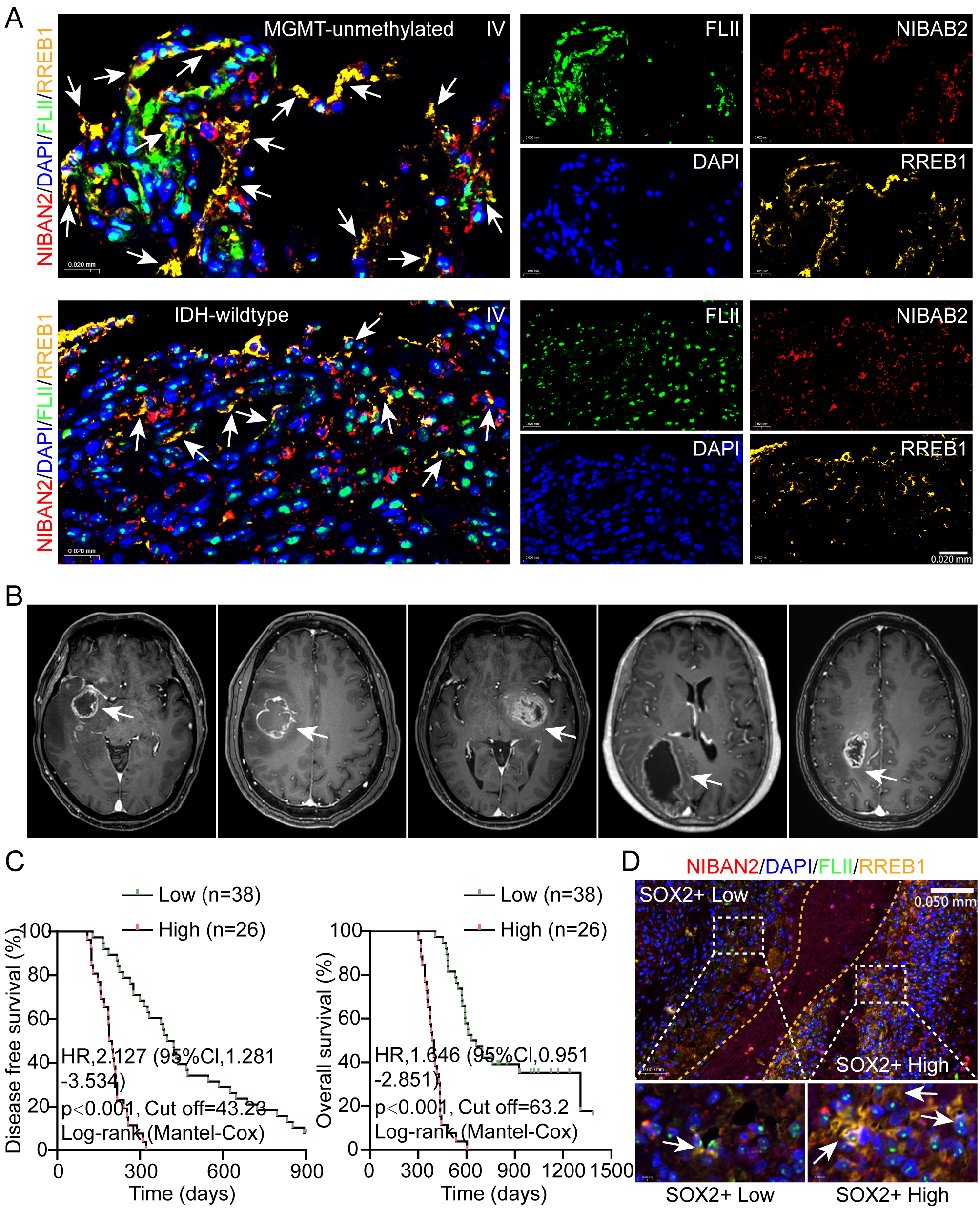


**Supplementary Fig. 20 Related to Fig. 10**

**(A)** NIBAN2/FLII/RREB1 expression stratified by MGMT promoter methylation and IDH status. Elevated expression levels of the feedback loop components in MGMT-unmethylated and IDH-wildtype glioma subgroups. **(B)** Typical MRI imaging features in patients with co-expression of the loop components include tumor necrosis, increased angiogenesis, and diffusely infiltrative tumor margins. **(C)** Prognostic impact of NIBAN2/FLII/RREB1 co-expression. Kaplan-Meier survival curves demonstrating that high co-expression correlates with shorter PFS and OS in glioma patients. **(D)** Spatial co-localization of the feedback loop in SOX2⁺ regions. Dual immunofluorescence revealing nuclear co-localization of NIBAN2, FLII, and RREB1 within SOX2-positive GSCs-enriched niches.


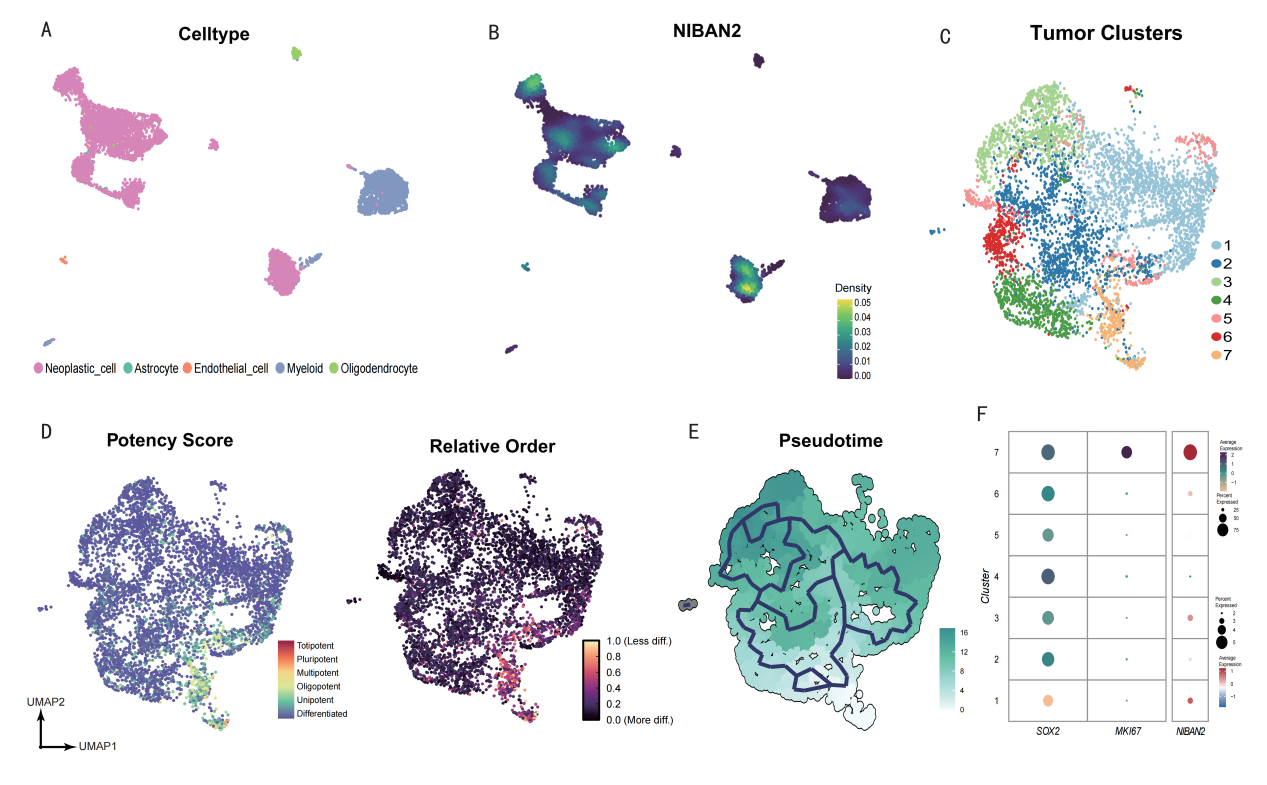


**Supplementary Fig. 21 Related to Fig. 10**

Relatively high NIBAN2 expression is associated with a tumor cell subpopulation exhibiting stem-like features and elevated differentiation potential in human glioma.

**(A)** UMAP visualization of integrated single-cell transcriptomes from four glioma specimens (GSM4119531–GSM4119534), showing global cellular heterogeneity. **(B)** Density estimation of NIBAN2 expression across all cells, suggesting possible enrichment in tumor regions. **(C)** UMAP embedding of malignant cells only, annotated by transcriptionally defined clusters (Clusters 1-7). **(D)** Differentiation potential of tumor cells inferred by CytoTRACE2; color gradient represents CytoTRACE scores (high = less differentiated). **(E)** Pseudotime trajectory analysis placing Cluster 7 at the origin of the inferred developmental path, consistent with its high potency. **(F)** Dot plot depicting average expression (color intensity) and percentage of expressing cells (dot size) for SOX2, MKI67 (Ki67), and NIBAN2 across tumor clusters. Cluster 7 shows the highest co-expression of NIBAN2, SOX2, and MKI67, indicative of a stem-like, aggressive phenotype.
